# Breast cancer risk factors and their effects on survival: a Mendelian randomisation study

**DOI:** 10.1186/s12916-020-01797-2

**Published:** 2020-11-17

**Authors:** Maria Escala-Garcia, Anna Morra, Sander Canisius, Jenny Chang-Claude, Siddhartha Kar, Wei Zheng, Stig E. Bojesen, Doug Easton, Paul D. P. Pharoah, Marjanka K. Schmidt

**Affiliations:** 1 Division of Molecular Pathology, The Netherlands Cancer Institute - Antoni van Leeuwenhoek Hospital, Amsterdam, The Netherlands; 2 Division of Molecular Carcinogenesis, The Netherlands Cancer Institute - Antoni van Leeuwenhoek Hospital, Amsterdam, The Netherlands; 3 Division of Cancer Epidemiology, German Cancer Research Center (DKFZ), Heidelberg, Germany; 4 University Medical Center Hamburg-Eppendorf, University Cancer Center Hamburg (UCCH), Cancer Epidemiology Group, Hamburg, Germany; 5 MRC Integrative Epidemiology Unit, University of Bristol, Bristol, UK; 6 Population Health Sciences, Bristol Medical School, University of Bristol, Bristol, UK; 7 Division of Epidemiology, Department of Medicine, Vanderbilt Epidemiology Center, Vanderbilt-Ingram Cancer Center, Vanderbilt University School of Medicine, Nashville, TN USA; 8 Copenhagen University Hospital, Copenhagen General Population Study, Herlev and Gentofte Hospital, Herlev, Denmark; 9 Copenhagen University Hospital, Department of Clinical Biochemistry, Herlev and Gentofte Hospital, Herlev, Denmark; 10 Faculty of Health and Medical Sciences, University of Copenhagen, Copenhagen, Denmark; 11 Department of Oncology, Centre for Cancer Genetic Epidemiology, University of Cambridge, Cambridge, UK; 12 Department of Public Health and Primary Care, Centre for Cancer Genetic Epidemiology, University of Cambridge, Cambridge, UK; 13 Division of Psychosocial Research and Epidemiology, The Netherlands Cancer Institute - Antoni van Leeuwenhoek Hospital, Amsterdam, The Netherlands

**Keywords:** Breast cancer survival, Breast cancer risk factors, Lifestyle, Mendelian randomisation, GWAS Catalog

## Abstract

**Background:**

Observational studies have investigated the association of risk factors with breast cancer prognosis. However, the results have been conflicting and it has been challenging to establish causality due to potential residual confounding. Using a Mendelian randomisation (MR) approach, we aimed to examine the potential causal association between breast cancer-specific survival and nine established risk factors for breast cancer: alcohol consumption, body mass index, height, physical activity, mammographic density, age at menarche or menopause, smoking, and type 2 diabetes mellitus (T2DM).

**Methods:**

We conducted a two-sample MR analysis on data from the Breast Cancer Association Consortium (BCAC) and risk factor summary estimates from the GWAS Catalog. The BCAC data included 86,627 female patients of European ancestry with 7054 breast cancer-specific deaths during 15 years of follow-up. Of these, 59,378 were estrogen receptor (ER)-positive and 13,692 were ER-negative breast cancer patients. For the significant association, we used sensitivity analyses and a multivariable MR model. All risk factor associations were also examined in a model adjusted by other prognostic factors.

**Results:**

Increased genetic liability to T2DM was significantly associated with worse breast cancer-specific survival (hazard ratio [HR] = 1.10, 95% confidence interval [CI] = 1.03–1.17, *P* value [*P*] = 0.003). There were no significant associations after multiple testing correction for any of the risk factors in the ER-status subtypes. For the reported significant association with T2DM, the sensitivity analyses did not show evidence for violation of the MR assumptions nor that the association was due to increased BMI. The association remained significant when adjusting by other prognostic factors.

**Conclusions:**

This extensive MR analysis suggests that T2DM may be causally associated with worse breast cancer-specific survival and therefore that treating T2DM may improve prognosis.

## Background

Breast cancer is a heterogeneous disease with a broad variation in prognosis [1]. Providing a precise prognostication for breast cancer patients is important in order to inform them accurately about the course of the disease and to allocate them to the right treatment [2]. To date, most commonly used prognostic factors relate to tumour characteristics and the extent of the disease at the time of diagnosis [2]. Many observational studies have evaluated the association of breast cancer risk and survival with other patient characteristics and lifestyle-related risk factors [3–5]. However, due to their observational nature, it is difficult for these studies to establish causation. Understanding whether or not the association between breast cancer survival and risk factors is causal might influence strategies to improve survival in breast cancer patients. In theory, randomised control trials (RCTs) provide a reliable method to evaluate the causal relationship between risk factors and survival [6, 7], but they are often not feasible as they can be prohibitively expensive, time-consuming, and even unethical. If an RCT cannot be performed to assess the causal effect between a risk factor and the outcome of interest, methods using instrumental variables may be an alternative.

Mendelian randomisation (MR) is a popular analytical method that uses genetic variants as instrumental variables (i.e. genetic instruments). This methodology uses a genetic predictor for the risk factor. Because of the natural randomisation of alleles during meiosis, this genetic predictor will be independently distributed across a population. Theoretically, therefore, this genetic instrument is not affected by potential environmental confounding factors or by disease status. MR rests on three basic assumptions: (1) genetic variants are associated with the risk factor (relevance assumption), (2) those genetic variants are not associated with any known or unknown confounders (independence assumption), and (3) the genetic variants affect the outcome only through the risk factor (exclusion restriction assumption) [8]. Using a genetic score that combines multiple variants explaining a large *R*-squared of the risk factor can help reducing the probability of violating the first MR assumption and providing more powerful MR analyses. The third assumption is also known as independence from horizontal pleiotropy, which occurs when the genetic variants influence the outcome by means of other pathways independently of the risk factor [8]. Several methods and sensitivity tests exist to assess these assumptions [9].

In this study, we used MR analysis to evaluate the causal relationships between breast cancer-specific survival and nine established risk factors for breast cancer: alcohol consumption, body mass index (BMI), height, mammographic density, menarche (age at onset), menopause (age at onset), physical activity, smoking, and type 2 diabetes mellitus (T2DM). Observational studies have provided evidence for the potential association of these risk factors and breast cancer survival, sometimes with conflicting results.

A population-based prospective study found that smoking before or after breast cancer diagnosis is associated with worse breast cancer survival [10]. Another meta-analysis of cohort studies concluded that current smoking is associated with worse breast cancer-specific survival compared to never smoking in breast cancer patients [11]. Obesity (BMI of ≥ 30.0) has been associated with worse breast cancer survival in a meta-analysis and systematic review [12]. In another review, obesity was associated with worse breast cancer prognosis for women of all ages [13]. For T2DM, a retrospective study of breast cancer patients found that diabetes was independently associated with poorer breast cancer prognosis [14]. In a population-based study, breast cancer-specific mortality was higher among women with diabetes compared to non-diabetic patients [15]. In relation to menstrual risk factors, a population-based study showed that early age at menarche was significantly associated with poorer survival but age at menopause did not have a significant impact [16]. The relationship between mammographic density and breast cancer survival has been studied in several cohort studies, but results have been inconclusive [17–19]. For other factors such as physical activity, the evidence is also not clear: in an RCT with an 8-year follow-up, no significant difference in disease-free survival was found between an exercise group and a usual care group [20]. To date, there is no evidence for an association between height or post-diagnosis alcohol consumption and breast cancer survival [21].

Our hypothesis was that some of these risk factors, for which there is evidence of an association with breast cancer survival based on observational data, might have a causal association with breast cancer-specific survival. We also aimed to investigate whether we could observe—or refute—an effect for the risk factors for which the association is not clear. We therefore performed a two-sample MR analysis using genetic variants and risk factor association summary estimates from the GWAS Catalog [22] and breast cancer survival summary estimates from the Breast Cancer Association Consortium (BCAC) cohort [23].

## Methods

### Selection of risk factors

We first considered the full list of breast cancer risk factors provided on the Cancer Research UK site [33] as of January 2020 (Additional file 1: Table S1). From this list of 25 factors, we identified nine factors for which genome-wide association study (GWAS) data were available. Only GWASs that could be directly downloaded from GWAS Catalog [22] into TwoSampleMR [34] R package were considered. If there were multiple GWAS for one risk factor, we selected the study with the largest sample size from those that were predominantly of European ancestry (Table 1). We considered only genome-wide significant variants (*P* < 5 × 10^−8^) to ensure that the association with the risk factor was robust (first MR assumption). Only single-nucleotide polymorphisms (SNPs) were considered as the reference panel did not include other types of variants. Variants correlated with the most significant SNPs were removed so that only uncorrelated variants remained in the analysis (*r*^2^ < 0.001). We calculated a priori power to detect an association at a significant level of 0.05 for each risk factor using the tool (https://sb452.shinyapps.io/power) [35]. We used the number of events (*n* = 7054) as sample size.

**Table 1:** Description of the nine risk factors with available genetic data from GWAS

Risk factor (units)	Number* of SNPs	GWAS Catalog accession number	Study reference	Sample size	Population
Alcohol consumption (drinks per week)^†^	98	GCST007472	Liu et al. [24]	1,039,210	100% European
Body mass index (adult, kg/m^2^)	100	GCST006368	Hoffmann et al. [25]	334,487	81% European
Height (adult, m)	112	GCST002647	Wood et al. [26]	253,288	100% European
Mammographic density (dense vs non-dense area)	5	GCST002667	Lindström et al. [27]	16,015**	100% European
Menarche (age at onset)	82	GCST002541	Perry et al. [28]	182,413**	100% European
Menopause (age at onset)	34	GCST005312	Day et al. [29]	69,626**	100% European
Physical activity (overall physical activity time)	3	GCST006912	Doherty et al. [30]	91,105	100% European
Smoking behaviour (ever vs never)^‡^	119	GCST007327	Karlsson Linnér et al. [31]	518,633	100% European
Type 2 diabetes mellitus (yes vs no)	95	GCST006867	Xue et al. [32]	659,316 (62,892 cases and 596,424 controls)	99% European

*The number of SNPs here may be lower than in the GWAS due to filtering (see the “Methods” section)

**Partial overlap with samples from BCAC

^†^Defined as the average number of drinks a participant reported drinking each week, aggregated across all types of alcohol

^‡^This is a binary phenotype. Participants who reported ever being a regular smoker in their life (current or former) were coded “2”, while participants who reported never being a regular smoker in their life were coded “1”

### Breast cancer survival and genetic data

The breast cancer survival data was obtained from the Breast Cancer Association Consortium (BCAC). We analysed clinic-pathological data (database version 12) and genotype data from the OncoArray [36] and iCOGS arrays [37]. The analysis included 86,627 female patients of European ancestry diagnosed at age > 18 years with invasive breast cancer of any stage. The dataset included 7054 breast cancer-specific deaths. A total of 59,378 patients (4246 deaths) had ER-positive disease, and 13,692 (1733 deaths) had ER-negative disease. Genotypes for variants not present on the arrays were imputed using the Haplotype Reference Consortium [38] as reference panel. Details about the genotyping, sample quality control, and imputation procedure have been described previously [36, 39]. Our analyses were based on SNPs that were imputed with imputation *r*^2^ > 0.7 and had minor allele frequency > 0.01 in at least one of the two datasets (iCOGS or OncoArray).

### Breast cancer survival estimates

We took the SNPs referred to in Table 1 as genetic instruments for each of the nine risk factors. For every SNP, we performed survival analyses to obtain survival estimates as described previously [23]. The analyses included the full OncoArray and iCOGS datasets. Time at risk was calculated from the date of diagnosis with left truncation for prevalent cases. Follow-up was right censored on the date of death, last date known alive if death did not occur, or at 15 years after diagnosis, whichever came first [39]. We estimated the association between the genetic instruments and breast cancer-specific survival using Cox proportional hazards regression [40]. The models were stratified by study and included the first two ancestry informative principal components, based on the genotyping array data as previously described, to adjust for population structure [36, 37]. We analysed the OncoArray and iCOGS datasets separately and then combined the estimates using fixed-effect meta-analyses [39]. Analyses were carried out for all invasive breast cancer and for estrogen receptor (ER)-positive and ER-negative disease separately. Additional file 2: Tables S1-S9 provides the full list of SNPs used and the corresponding estimates for the per-allele risk factor effect sizes and the per-allele survival log (hazard ratios).

### MR statistical analyses and sensitivity diagnostics

We used the TwoSampleMR [34] R package to perform the two-sample MR analyses. We obtained the genetic instruments for the risk factors (MR-Base NHGRI-EBI GWAS Catalog [22], 29 August 2019 update), harmonised the SNP effects so they corresponded to the same allele for the risk factor and survival associations, and performed the sensitivity tests. We estimated the causal relationships between each of the sets of SNPs for the nine risk factors and breast cancer-specific survival using the inverse-variance weighted (IVW) method. We performed the analyses for all invasive breast cancer, ER-positive, and ER-negative separately. The association of BMI with breast cancer-specific survival was previously evaluated in an earlier, smaller version of the BCAC dataset (*n* = 36,210) [41]. In this analysis, we included more patients, updated follow-up, and a larger BMI GWAS genetic instrument. It has been suggested that the potential negative effect of BMI on survival is especially relevant in postmenopausal women [12]. Therefore, we also tested whether the BMI associations differed between pre- (age at diagnosis under 50 years, *n* = 27,009 with 2680 breast cancer-specific deaths) and postmenopausal women (age at diagnosis 50 years or older, *n* = 59,617 with 4374 breast cancer-specific deaths). Inclusion of even a small percentage of a different ethnic group can affect the interpretation and validity of the causal estimates [42]. Because the genetic instrument that we used for BMI had 19% of non-European participants, we performed an additional analysis using the BMI European-specific summary estimates from the same GWAS available at the author’s supplementary material [25] (61 SNPs after filtering, Additional file 2: Table S10).

IVW assumes that none of the variants exhibit horizontal pleiotropy, which may not be true in practice. Therefore, we also used the MR-Egger regression method that allows variants to demonstrate unbalanced pleiotropic associations. That is, MR-Egger regression relaxes the requirement of no horizontal pleiotropy provided that the pleiotropic effects are not proportional to the effects of the variants on the risk factors of interest [8, 9]. In comparison to the IWM, the MR-Egger method’s intercept is not constrained to zero and provides a statistical test of the extent to which this intercept differs from zero as a measure of unbalanced pleiotropic effects.

For the risk factors with a significant association based on the IVW method (false discovery rate [FDR] < 0.05), we ran the following sensitivity analyses: heterogeneity tests, funnel plots, and leave-one-out tests. To assess the robustness of the results of the IVW method, we applied other MR methods (simple mode, weighted median, and weighted mode). We also tested all associations by performing the analysis using a multivariable model. In the multivariable model, we used imputed phenotypes [43] and adjusted for the following known prognostic factors: age of the patients at diagnosis; tumour size; node status; distant metastasis status; grade; ER-, progesterone receptor, and HER2-status; and (neo) adjuvant chemotherapy, adjuvant anti-hormone therapy, and adjuvant trastuzumab. Because breast cancer survival can differ on the short or longer term, we also assessed whether or not the associations would hold for the 5-year horizon, which is typically used in breast cancer prognostication [44]. For this analysis, we reduced the follow-up time from 15 to 10 years (*n* = 85,470 with 6147 breast cancer-specific deaths) and 5 years (*n* = 79,183 with 3573 breast cancer-specific deaths). Both in the multivariable model and the shorter follow-up analyses, we performed the MR analyses separately for OncoArray and iCOGS datasets and meta-analysed the results.

### Relationships between BMI, T2DM, and breast cancer survival

To ensure that the effects of BMI and T2DM were independent, we identified SNPs that overlapped between the genetic instruments for these risk factors. Two SNPs, rs7144011 and rs7903146, were present in both the BMI and T2DM instrumental variables, and 12 (six pairs) SNPs were in linkage disequilibrium (LD): rs2972144, rs4072096, rs1801282, rs1899951, rs2112347, rs2307111, rs4715210, rs72892910, rs244415, rs889398, rs6059662, and rs6142096. We removed those 14 SNPs from the analyses to reduce the likelihood of horizontal pleiotropy. To further isolate the association of T2DM alone, we performed a multivariable MR model [45] by additionally including the genetically predicted BMI score as a covariate in the analyses of T2DM.

## Results

**Fig. 1:**
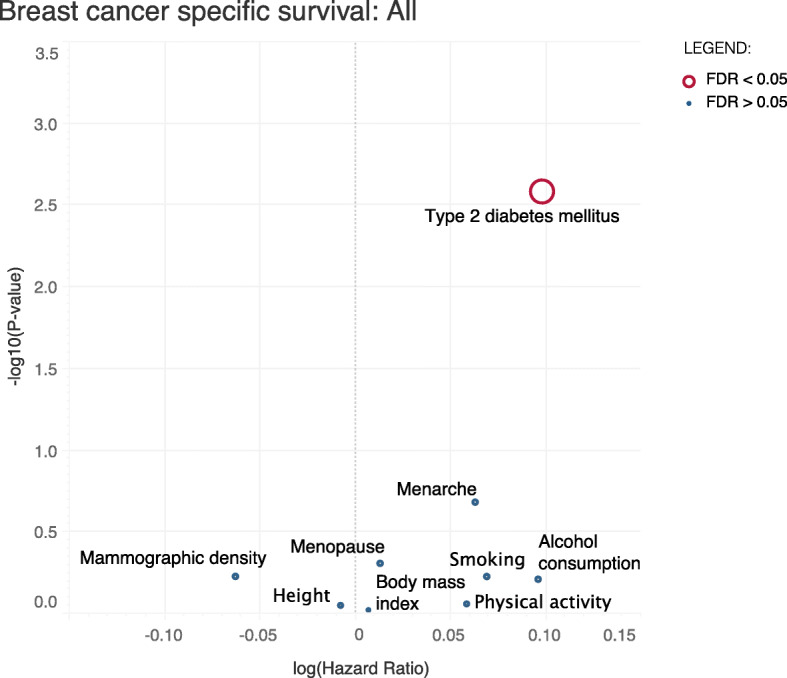
Effect of the nine breast cancer risk factors on breast cancer-specific survival in all breast cancers. The *y*-axis shows the −log_10_(*P* value) effect for the association. The *x*-axis corresponds to log (hazard ratio) effect for each of the traits on breast cancer survival. The risk factors with false discovery rate (FDR) < 0.05 are coloured in red; the size of the circle is proportional to the −log_10_(FDR)

We found a significant association between genetic liability to T2DM and breast cancer-specific survival (*P* < 0.05, Table 2). For all breast cancers, T2DM was associated with worse breast cancer-specific survival (hazard ratio [HR] = 1.10, 95% confidence interval [CI] = 1.04–1.18, *P* value [*P*] = 0.003, FDR = 0.023) (Fig. 1 and Table 2). T2DM was also associated with worse breast cancer-specific survival when restricting to ER-positive cases. The effect in the ER-positive subtype was consistent (HR = 1.09, CI = 1.01–1.18, *P* = 0.036, FDR = 0.324) with the effect in all breast cancers. We did not observe associations at FDR < 0.05 (Table 2) between survival, for all breast cancer or by ER-subtype, and any of the other risk factors: alcohol consumption, BMI, height, mammographic density, menarche, menopause, physical activity, and smoking. The estimates we obtained from the models adjusted by other known prognostic factors (Additional file 1: Table S2) were comparable to the initial unadjusted analyses for all risk factors. Under the current sample size of our study (*n* = 86,627 and 7054 events), the power to detect a causal association varied considerably between risk factors (Additional file 1: Table S3). The estimated power was the largest for age at menopause and lowest for physical activity.

**Table 2:** Effect of nine breast cancer risk factors on breast cancer-specific survival for all breast cancers, estrogen receptor (ER)-positive and ER-negative breast cancers. *HR* hazard ratio, *CI* 95% confidence interval, *FDR* false discovery rate

Risk factor	All				ER-positive				ER-negative			
	HR	CI	*P* value	FDR	HR	CI	*P* value	FDR	HR	CI	*P* value	FDR
Alcohol consumption (drinks per week)	1.10	0.76–1.60	0.62	0.92	1.12	0.69–1.81	0.65	0.65	0.91	0.42–1.97	0.82	0.90
Body mass index (adult, kg/m^2^)	1.01	0.81–1.25	0.95	0.95	1.10	0.83–1.45	0.52	0.62	1.14	0.72–1.82	0.57	0.88
Height (adult, m)	0.99	0.89–1.11	0.89	0.95	1.04	0.91–1.21	0.55	0.62	1.08	0.82–1.27	0.88	0.90
Mammographic density (dense vs non-dense area)	0.94	0.75–1.18	0.59	0.92	0.90	0.75–1.08	0.24	0.59	1.12	0.79–1.60	0.52	0.88
Menarche (age at onset)	1.07	0.97–1.18	0.21	0.92	1.06	0.94–1.21	0.34	0.61	1.06	0.86–1.29	0.59	0.88
Menopause (age at onset)	1.01	0.98–1.05	0.49	0.92	1.03	0.98–1.08	0.21	0.59	1.01	0.93–1.08	0.90	0.90
Physical activity (overall physical activity time)	1.06	0.52–2.16	0.87	0.95	1.66	0.48–5.71	0.43	0.62	0.36	0.09–1.54	0.17	0.77
Smoking behaviour (ever vs never)	1.07	0.83–1.38	0.59	0.92	0.82	0.58–1.16	0.26	0.59	1.53	0.92–2.54	0.10	0.77
Type 2 diabetes mellitus (yes vs no)	1.10	1.04–1.18	0.01	0.02	1.09	1.01–1.18	0.04	0.32	1.09	0.97–1.24	0.15	0.88

### Genetic association between BMI by menopausal status and breast cancer-specific survival

We found no association between BMI and breast cancer-specific survival in any of the analysed subtypes, nor by menopausal status (*P* > 0.05): premenopausal (HR = 1.06, CI = 0.78–1.44, *P* = 0.710) or postmenopausal women (HR = 1.02, CI = 0.80–1.30, *P* = 0.899). The estimate using the European-specific BMI genetic instrument (HR = 1.14, CI = 0.94–1.38, *P* = 0.174) was also not significant.

### Genetic association between T2DM and breast cancer-specific survival

The HR estimate for T2DM and survival among all invasive breast cancers (HR = 1.10) was higher than that for either ER-subtype individually (ER-positive: HR = 1.09; ER-negative: HR = 1.09). This reflected the fact that the patients without ER-status information (*n* = 13,557) had a larger risk estimate (HR = 1.19, CI = 1.02–1.39, *P* = 0.023).

To further validate the association between T2DM and breast cancer-specific survival, we performed the analysis using a shorter follow-up. The results were significant and similar to the main analysis both for 10-year (HR = 1.12, CI = 1.05–1.19, *P* = 0.0006) and for 5-year follow-up (HR = 1.13, CI = 1.04–1.23, *P* = 0.005). We also tested the association in a model adjusted by other known prognostic factors. The association of T2DM with breast cancer-specific survival in the adjusted model was still significant (HR = 1.10, CI = 1.02–1.18, *P* = 0.013), and the effect size remained similar to the main T2DM analysis (HR = 1.10, CI = 1.04–1.18, *P* = 0.003). Finally, we tried to replicate the result using another large and well-powered GWAS, i.e. the T2DM summary estimates from the DIAGRAM GWAS which is a large meta-analysis of 32 studies comprising data for 898,130 individuals (74,124 T2DM cases and 824,006 controls) of European ancestry [46]. The genetic instrument for this dataset included 152 SNPs (12 SNPs overlapping with the T2DM genetic instrument we initially used, Additional file 2: Table S11). The association of T2DM with breast cancer-specific survival using the replication dataset was significant (HR = 1.18, CI = 1.04–1.33, *P* = 0.009) and similar to the initial result (HR = 1.10).

### Association between T2DM and breast cancer-specific survival with BMI adjustment

To explore the potential confounding effect of BMI with T2DM, we performed an analysis adjusting for genetically predicted BMI. The effect of BMI in this analysis was not significant (HR = 1.02, CI = 0.85–1.24, *P* = 0.809), and the effect of T2DM on survival was similar (HR = 1.10, CI = 1.04–1.17, *P* = 0.002) to the main T2DM analysis (HR = 1.10, CI = 1.04–1.18, *P* = 0.003).

### Causal association between T2DM and breast cancer-specific survival

**Fig. 2:**
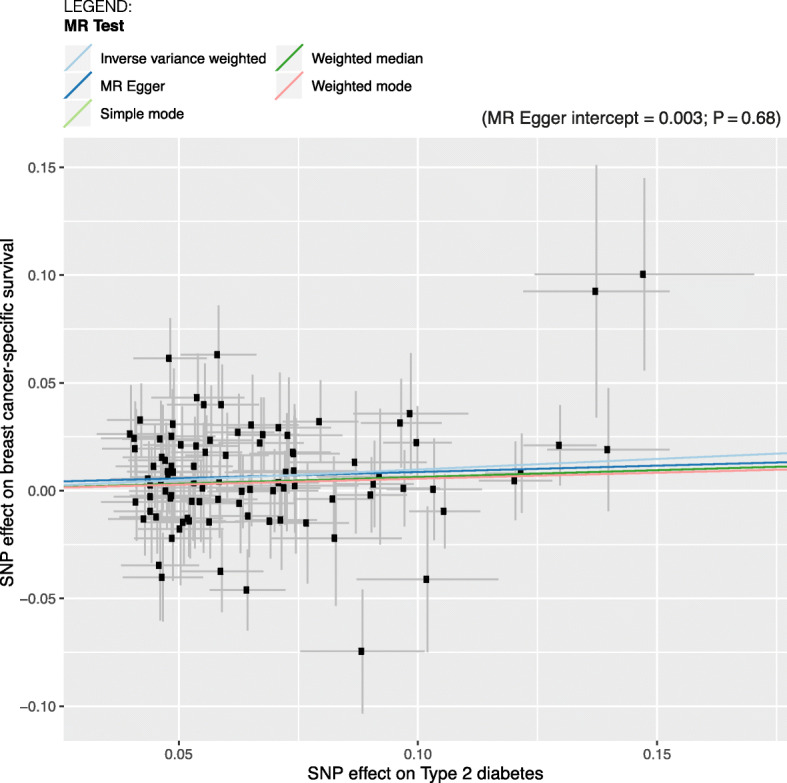
Plot showing the effect sizes of the SNP effects on breast cancer-specific survival for all breast cancers (*y*-axes) and the SNP effects on T2DM (*x*-axes) with 95% confidence intervals. Each dot represents one of the 95 SNPs used in the T2DM genetic instrument. The slopes indicate the estimate for each of the five different MR tests

We used different variations of the MR method to assess possible violations of the MR assumptions. Figure 2 shows that the range of MR methods used (simple mode, weighted median, and weighted mode) to assess the sensitivity of the findings all gave similar effect size estimates. Additionally, there was no evidence of pleiotropy based on the MR-Egger intercept test (MR-Egger intercept = 0.003, *P* = 0.68, Fig. 2). In analyses using funnel plot (Additional file 1: Figure S1) and a leave-one-out test (Additional file 1: Figure S2), there was no indication for violation of the assumptions, nor that the association was driven by any particular SNP.

## Discussion

We performed a Mendelian randomisation analysis to explore the potential causal effects on breast cancer-specific survival of nine established risk factors for breast cancer: alcohol consumption, BMI, height, mammographic density, menarche, menopause, physical activity, smoking, and T2DM. We used survival estimates from 86,627 European breast cancer patients with invasive breast cancer (by far the largest such dataset) and summary data from the GWAS Catalog for the nine risk factors. We used the IVW method to estimate causal effects and performed a wide range of sensitivity analyses to test the robustness of our findings.

Our analysis showed an association between genetic liability to T2DM and worse breast cancer-specific survival. The IVW method result was consistent with the results of other complementary MR-methods, and the performed sensitivity analyses did not give any statistical indication for violations of the MR assumptions. Additionally, the T2DM GWAS used was reasonably powered, with an estimated heritability of ~ 20% [32], supporting the relevance assumption. There was no evidence that the SNPs were associated with breast cancer survival (exclusion restriction). Finally, the association remained significant when adjusting for other known prognostic factors and when shortening the follow-up time to 10 and 5 years.

Because obesity and T2DM share some biological features such as elevated insulin levels, hypertension, and chronic inflammation [47] and since higher BMI has been associated with increased incidence of T2DM [48], we explored a possible interaction between the two risk factors. First, we ensured that there were no common SNPs between the T2DM and BMI genetic instruments or SNPs in LD that could be driving the association. Second, we performed BMI-adjusted analyses which also showed that the association was being driven by T2DM and not by BMI.

Earlier literature suggests an association between diabetes and worse breast cancer-specific survival [49–51]. There is no clear evidence linking diabetes to any particular ER-status specific breast cancer subtype [52] that could explain the poorer survival in women with T2DM. The increased mortality in patients with T2DM might be explained by the effect of insulin resistance or hyperinsulinemia, since breast cancer cells might have a selective growth advantage because of insulin receptor overexpression [53, 54]. However, to our knowledge, no functional studies to evaluate this have yet been carried out. An important point to consider when interpreting the results is that, when using a binary risk factors such as T2DM, the genetic instrument estimate will only represent the average causal effect of the exposure in a fraction of the studied population (named “genetic compliers”). Additionally, the latter would only be true assuming that the monocity assumption is plausible, which means that increasing number of alleles for an individual would increase (or maintain constant) the risk of having T2DM [55].

All the other risk factors gave null results. Some of these may reflect the fact that there is no true association, but others may be underpowered since the fraction of variation of the risk factor explained by the genetic instrument was too small. The heritability explained by identified SNPs, and hence the power of the genetic instruments, varies substantially between risk factors, e.g. ~ 20% for T2DM [32] versus only 1% for the mammographic density GWAS [27]. In addition, we only kept genome-wide significant SNPs and dropped all SNPs in LD or with low imputation quality in the BCAC dataset, so the explained variation that we could utilise was smaller. As GWAS become larger and more powerful genetic instruments are available, it may be possible to find associations that could not be identified here. However, for those risk factors with a predicted small genetic component (e.g. physical activity), their association with breast cancer survival might not be assessable using an MR framework [8]. A potential limitation of our study is that some patients in the breast cancer survival dataset were also included in the GWASs for the risk factors, mammographic density (~ 2.5% overlap) and age at menarche (~ 27%) and menopause (~ 21%). However, because the genetic instruments of age at menarche and menopause were relatively strong and there was little overlap for mammographic density, we may expect the bias caused by patient overlap to be small [56]. Finally, another potential reason for which we did not observe association for some risk factors might be due to selection bias. This type of collider bias can lead to an under- or overidentification of genetic risk factors for breast cancer survival due to a relationship between the genetic risk factor concerned and breast cancer incidence [57]. This could be the case for BMI, age at menopause and menarche, or height, which have been causally associated with breast cancer risk [58]. For other risk factors such as T2DM or smoking, MR studies of incidence could not provide evidence for a causal association [59, 60], which makes these genetic instruments less likely to be affected by selection bias.

To further explore the link between BMI and breast cancer survival, we also tested separately for pre- and postmenopausal status, but there was no indication for an association in any of the menopausal groups. Despite the evidence for an association between BMI and breast cancer survival from observational studies [12, 13], our analysis on BMI and breast cancer-specific survival did not confirm this. A possible explanation is that obesity is associated with other comorbid conditions [48] that lead to poorer overall, but no breast cancer-specific survival. Additionally, it has been suggested that obese patients might receive suboptimal chemotherapy treatment compared to regular weight women [61] and tumours are usually detected at a later stage in obese patients [62]. This would, if insufficiently corrected for, lead to an association between high BMI and worse breast cancer-specific survival in observational, but not in MR, studies. The different observations of the relationship between BMI and survival from MR versus observational studies resemble those of genetic BMI and breast cancer risk [63], which were also deviant from epidemiological studies. To date, there is not a clear answer as to whether and how high BMI directly influences the biology of cancer [64].

From a clinical point of view, our analysis suggests that genetic liability to T2DM may contribute to variation in breast cancer outcomes in women of European ancestry. Such a genetic predictor might be included in prognostication models aimed at identifying women most likely to benefit from specific interventions. Furthermore, even though T2DM has a genetic component, it is also influenced by environmental and lifestyle factors and is potentially preventable [65]. Although our study does not address this directly, it seems sensible to recommend intensified management of T2DM, including lifestyle changes, in breast cancer patients.

The main strength of our study is the use of the biggest breast cancer dataset available so far and the use of SNPs as genetic instruments to reduce potential confounding. Despite including more than 7000 breast cancer-specific deaths in the analyses, our study was not well powered especially for the analysis within the subset of ER-negative tumours (as indicated by the broad confidence intervals). Additional findings might be possible when there are larger sample sizes available and a more complete follow-up. We also lacked power to detect associations for certain risk factors that had only a handful of SNPs in their genetic instruments such as mammographic density and physical activity. Finally, our results are applicable to women of European ancestry only. In order to be able to generalise these findings to other ancestry groups, larger breast cancer datasets are needed for the other ethnicities.

## Conclusion

This two-sample MR analysis suggests that genetic liability to T2DM might be a cause of reduced breast cancer-specific survival. Our study provides further evidence for the importance of promoting a healthier lifestyle to improve survival in breast cancer patients.

## Supplementary information

**Additional file 1: Table S1.** List of breast cancer risk factors as indicated by Cancer Research UK. Information in this table was taken directly from: https://www.cancerresearchuk.org/about-cancer/breast-cancer/risks-causes/risk-factors (January 2020). **Table S2.** Comparison of the effect of nine breast cancer risk factors on breast cancer-specific survival for all breast cancers in the unadjusted model (left) and in the adjusted model (right). The model was adjusted for the known prognostic factors: age of the patients at diagnosis, tumour size, node status, distant metastasis status, grade, ER-, progesterone receptor and HER2-status and (neo) adjuvant chemotherapy, adjuvant anti-hormone therapy and adjuvant trastuzumab. HR = Hazard Ratio. CI = 95% Confidence Interval. **Table S3.** Power (%) estimation by a range of Hazard Ratios (HR) for the analysis of MR associations between nine breast cancer risk factors and breast cancer-specific survival in all breast cancers. **Figure S1.** Funnel plot for T2DM and breast cancer-specific survival. The plot shows the effect estimate (b) of a particular SNP against the SNP expected precision (1/Standard Error (SE)). Asymmetry in the funnel plot is an indication of horizontal pleiotropy. The dark and light blue lines represent the MR-Egger and Inverse variance weighted slopes respectively. **Figure S2.** Leave-one-out plot for T2DM and breast cancer specific-survival showing the estimate effect by sequentially dropping one SNP at a time. Each black dot in the forest plot represents the MR results (IVW method) excluding that particular SNP. The result including all SNPs is shown in red at the bottom of the plot.

**Additional file 2: **SNPs used in the analyses for the nine risk factors. The risk factor estimates (beta and standard error (SE)) and breast cancer-specific survival estimates for each SNP are included. **Table S1.** Alcohol consumption. **Table S2.** Body mass index. **Table S3.** Height. **Table S4.** Mammographic density. **Table S5.** Menarche. **Table S6.** Menopause. **Table S7.** Physical activity. **Table S8.** Smoking behaviour. **Table S9.** Type 2 diabetes mellitus. **Table S10.** Body mass index European-specific. **Table S11.** Type 2 diabetes mellitus replicate.

## Data Availability

Not applicable.

## Abbreviations

BMI: Body mass index
BCAC: Breast Cancer Association Consortium
CI: Confidence interval
ER: Estrogen receptor
FDR: False discovery rate
GWAS: Genome-wide association study
HR: Hazard ratio
IVW: Inverse-variance weighted
LD: Linkage disequilibrium
MR: Mendelian randomisation
*P*: *P* value
RCT: Randomised control trial
SNP: Single-nucleotide polymorphism
T2DM: Type 2 diabetes mellitus
