# Supplementary material for: Breast cancer risk factors and their effects on survival: a Mendelian randomisation study
Source: BMC Med. 2020 Nov 17;18:327. doi: 10.1186/s12916-020-01797-2 (PMC7670589; doi:10.1186/s12916-020-01797-2)
Supplement: Supplementary file 1 — Additional file 1: Table S1. List of breast cancer risk factors as indicated by Cancer Research UK. Information in this table was taken directly from: https://www.cancerresearchuk.org/about-cancer/breast-cancer/risks-causes/risk-factors (January 2020). Table S2. Comparison of the effect of nine breast cancer risk factors on breast cancer-specific survival for all breast cancers in the unadjusted model (left) and in the adjusted model (right). The model was adjusted for the known prognostic factors: age of the patients at diagnosis, tumour size, node status, distant metastasis status, grade, ER-, progesterone receptor and HER2-status and (neo) adjuvant chemotherapy, adjuvant anti-hormone therapy and adjuvant trastuzumab. HR = Hazard Ratio. CI = 95% Confidence Interval. Table S3. Power (%) estimation by a range of Hazard Ratios (HR) for the analysis of MR associations between nine breast cancer risk factors and breast cancer-specific survival in all breast cancers. Figure S1. Funnel plot for T2DM and breast cancer-specific survival. The plot shows the effect estimate (b) of a particular SNP against the SNP expected precision (1/Standard Error (SE)). Asymmetry in the funnel plot is an indication of horizontal pleiotropy. The dark and light blue lines represent the MR-Egger and Inverse variance weighted slopes respectively. Figure S2. Leave-one-out plot for T2DM and breast cancer specific-survival showing the estimate effect by sequentially dropping one SNP at a time. Each black dot in the forest plot represents the MR results (IVW method) excluding that particular SNP. The result including all SNPs is shown in red at the bottom of the plot. [file 12916_2020_1797_MOESM1_ESM.pdf]

Additional file 1

Table S1. List of breast cancer risk factors as indicated by Cancer Research UK. Information in this table was taken directly from: https://www.cancerresearchuk.org/about-cancer/breast-cancer/risks-causes/risk-factors (January 2020).

**Factors that increase the risk of breast cancer**	
**Risk factors**	**Description**
Being overweight or obese	Women who are overweight after their menopause have a higher risk of breast cancer than women who are not overweight. Men also have an increased risk of breast cancer if they are overweight or obese. Being overweight means having a body mass index (BMI) of between 25 and 30. Obesity means being very overweight with a BMI of 30 or higher. Try to keep a healthy weight by being physically active and eating a healthy, balanced diet.
Alcohol	Drinking alcohol increases the risk of breast cancer. The risk increases with each extra unit of alcohol per day. One unit is a half pint of beer, a small glass of wine, or a single measure of spirits. Latest UK government guidelines advise drinking no more than 14 units of alcohol a week.
Contraceptive pill	There is a very small increased risk of breast cancer when you take the contraceptive pill. This increase in risk goes back to normal 10 years after you stop taking it. Remember that breast cancer is rare in young women. Most women who take the pill are in their late teens, twenties and early thirties. So a small increase in this risk during the time women take the pill means very few extra cases of breast cancer.
Hormone replacement therapy (HRT)	Many women take hormone replacement therapy (HRT) to reduce menopausal symptoms. There are 2 main types of HRT: combined HRT (oestrogen and progesterone) and oestrogen only HRT. HRT increases the risk of breast cancer while women take it and for up to 5 years afterwards. Combined HRT (oestrogen and progesterone) is more likely to cause breast cancer than oestrogen only HRT. HRT can have some health benefits and so if you are worried about taking it, talk to your doctor about the benefits and risks in your individual situation.
Being inactive	There is a small increase risk of breast cancer if you're inactive, this means doing less than 150 minutes of moderate exercise a week. It can be easier than you think to increase even if you don't do much at the moment. Activity doesn't just mean sport and exercise. Anything that makes you a bit warmer and slightly out of breath counts as moderate activity.
**Risks that you can't change**	
Getting older	Most breast cancers occur in women over 50 and it is extremely rare in women under 40.

Family history and inherited genes	Some people have a higher risk of developing breast cancer than the general population because other members of their family have had particular cancers. This is called a family history of cancer. Having a mother, sister or daughter diagnosed with breast cancer approximately doubles the risk of breast cancer. This risk is higher when more close relatives have breast cancer, or if a relative developed breast cancer under the age of 50. But most women who have a close relative with breast cancer will never develop it. Some people have an increased risk of breast cancer because they have an inherited gene fault. We know about several gene faults that can increase breast cancer risk and there are tests for some of them. Having one of these faulty genes means that you are more likely to get breast cancer than someone who doesn't. But it is not a certainty. Remember that most breast cancers happen by chance. Only about 2 out of every hundred (2%) are related to a change in the BRCA1 or BRCA2 gene.
X–rays and radiotherapy	X-rays: Exposure to radiation is known to increase the risk of many types of cancer. Most of us are never exposed to enough radiation to make much difference to our risk. Nowadays, doctors keep medical exposure to radiation as low as possible. They don't do x-rays or CT scans unless they really need to. And the amount of radiation used is very small. Many women worry about having mammograms as part of breast screening because it exposes them to x-rays. But the amount of radiation you have with a mammogram is very small. Radiotherapy: Radiotherapy treatment for breast cancer increases the risk of getting breast cancer in the other breast by a small amount. But this small risk is balanced by the need to treat the original breast cancer. If you had radiotherapy to your chest area to treat another type of cancer your risk of developing breast cancer is higher than someone who hasn't had radiotherapy. This is especially so for women who have had chest radiotherapy for Hodgkin lymphoma in the past. If you need radiotherapy for Hodgkin lymphoma or any other type of cancer your doctors should tell you about this risk. They will offer you breast screening if it is appropriate. Talk to your doctor if you are unsure if you should have screening. It is important to remember that second cancers are usually found early when they can be successfully treated. Also, radiotherapy treatments are now more focused than in the past.
Other medical conditions	Women with **diabetes** have a small increase in their risk of breast cancer, although we are not sure why.
Dense breast tissue	Breast cancer risk is higher in women with the most dense breast tissue compared to less dense tissue. Women with dense breast tissue have less fat and more breast cells and connective tissue in their breasts. Our genetic make up affects breast density.
Benign breast disease	Benign breast disease means non cancerous breast conditions.
DCIS or LCIS	DCIS and LCIS are changes within the breast tissue that might develop into breast cancer in some women. DCIS stands for ductal carcinoma in situ. LCIS stands for lobular carcinoma in situ. Women with a diagnosis of DCIS or LCIS have double the usual risk of invasive breast cancer in the same or other breast. But it is important to remember that most women with LCIS or DCIS will not develop invasive cancer.
Age when periods started and stopped	You have an increased risk of breast cancer if your periods started early (before the age of 12). If you have a late menopause (after the age of 55) this increases your breast cancer risk compared to women who have an earlier menopause. This might be linked to hormone levels.

Sex hormones and other hormones	Levels of the female sex hormone, oestrogen, and the male hormone, testosterone, can affect the risk of breast cancer. Women have small amounts of the male hormone testosterone in their bodies. After the menopause, women with higher levels of oestrogen and testosterone in their blood have a higher risk of breast cancer compared to women with the lowest levels. Women with higher levels of testosterone in their blood before menopause have a higher risk of breast cancer. There is an increased risk of breast cancer in women with higher levels of a hormone called insulin like growth factor 1 (IGF-1). It is not clear what controls levels of IGF-1 in the bloodstream. It is probably related to our genes, body weight, and how much exercise we do.
Ethnicity	A large report found that the risk of breast cancer is higher in white women than any other ethnic group. This is at least partly due to lifestyle factors.
Previous cancer	Having had breast cancer increases your risk of getting another breast cancer. It might occur in the same breast or in the other breast.
Height	Women who are taller than average have a slightly increased risk of breast cancer after the menopause. This could be due to different hormone levels in taller women.
Not having children or having them later in life	Whether you can have children or when you have them may not be something you can control. Women who have children have a slightly lower risk of breast cancer than women who don't have children. The risk reduces further the more children you have.
**Factors that reduce the risk of breast cancer**	
Being physically active	Various studies have shown that physical activity can reduce the risk of breast cancer.An analysis of 31 studies found that women who did the most activity had a 12% lower risk of developing breast cancer compared with the least active women.
Breastfeeding	Breastfeeding lowers the risk of developing breast cancer, particularly if you have your children when you are younger. The longer you breastfeed the more the risk is reduced. It is not completely clear why this is. But the reduced risk might be because the ovaries don't produce eggs so often during breastfeeding. Or it might be because breastfeeding changes the cells in the breast so they might be more resistant to changes that lead to cancer.
Aspirin and anti inflammatory drugs	Research has shown that women who regularly take aspirin or other non steroidal anti inflammatory medicines have a slightly lower risk of developing breast cancer.
Medicines for women at higher risk	Some women who have family members with breast cancer have a higher risk of developing it than other women. Some medicines can help to prevent breast cancer in women at high risk. Taking a drug called anastrozole for 5 years can reduce the risk of breast cancer.
Diet	A healthy diet can help to prevent breast cancer.
**Where there isn't clear evidence**	
Diet and breast cancer risk	There has been a lot of research into whether diet increases the risk of breast cancer. So far most findings have been inconclusive and inconsistent.

Smoking	Smoking tobacco might increase your risk of getting breast cancer. It is never too late to stop smoking but the sooner you stop the better.
Night shift work	Some research has shown that working night shifts could possibly slightly increase breast cancer risk. But more recent research suggests that working night shifts is not likely to cause cancer, even if you have worked night shifts for a long time

Table S2. Comparison of the effect of nine breast cancer risk factors on breast cancer-specific survival for all breast cancers in the unadjusted model (left) and in the adjusted model (right). The model was adjusted for the known prognostic factors: age of the patients at diagnosis, tumour size, node status, distant metastasis status, grade, ER-, progesterone receptor and HER2-status and (neo)adjuvant chemotherapy, adjuvant anti-hormone therapy and adjuvant trastuzumab. HR = Hazard Ratio. CI = 95% Confidence Interval.

**Risk factor**	Unadjusted model			Adjusted model		
	HR	CI	P value	HR	CI	P value
Alcohol consumption (drinks per week)	1.1	0.76-1.60	0.62	1.21	0.82-1.77	0.34
Body mass index (adult, kg/m2)	1.01	0.81-1.25	0.95	1.03	0.85-1.25	0.79
Height (adult, m)	0.99	0.89-1.11	0.89	0.99	0.89-1.09	0.79
Mammographic density (dense vs non-dense area)	0.94	0.75-1.18	0.59	0.94	0.84-1.06	0.31
Menarche (age at onset)	1.07	0.97-1.18	0.21	1.05	0.95-1.60	0.33
Menopause (age at onset)	1.01	0.98-1.05	0.49	1.01	0.97-1.06	0.56
Physical activity (overall physical activity time)	1.06	0.52-2.16	0.87	1.02	0.58-2.50	0.62
Smoking behaviour (ever vs never)	1.07	0.83-1.38	0.59	1.16	0.90-1.50	0.24
Type 2 diabetes mellitus (yes vs no)	1.1	1.04-1.18	0.01	1.09	1.03-1.16	0.01

Table S3. Power (%) estimation by a range of Hazard Ratios (HR) for the analysis of MR associations between nine breast cancer risk factors and breast cancer-specific survival in all breast cancers.

**Risk factor**	**Estimated R^2**	**HR**		
		**0.9**	**1.1**	**1.3**
Alcohol consumption (drinks per week)	0.007	11.1	9.8	45.4
Body mass index (adult, kg/m^2)	0.012	16.1	13.9	67.5
Height (adult, m)	0.021	24.9	21.2	89.1
Mammographic density (dense vs non-dense area)	0.009	13.1	11.5	55.2
Menarche (age at onset)	0.017	21	18	81.9
Menopause (age at onset)	0.027	30.6	26	95.2
[illegible]	0.001	4.6	4.4	10.3
Smoking behavior	0.005	9.1	8.2	34.4
Type 2 diabetes mellitus (yes vs no)	0.020	23.9	20.4	87.6

Figure S1. Funnel plot for T2DM and breast cancer-specific survival. The plot shows the effect estimate (β) of a particular SNP against the SNP expected precision (1/Standard Error (SE)). Asymmetry in the funnel plot is an indication of horizontal pleiotropy. The dark and light blue lines represent the MR-Egger and Inverse variance weighted slopes respectively.

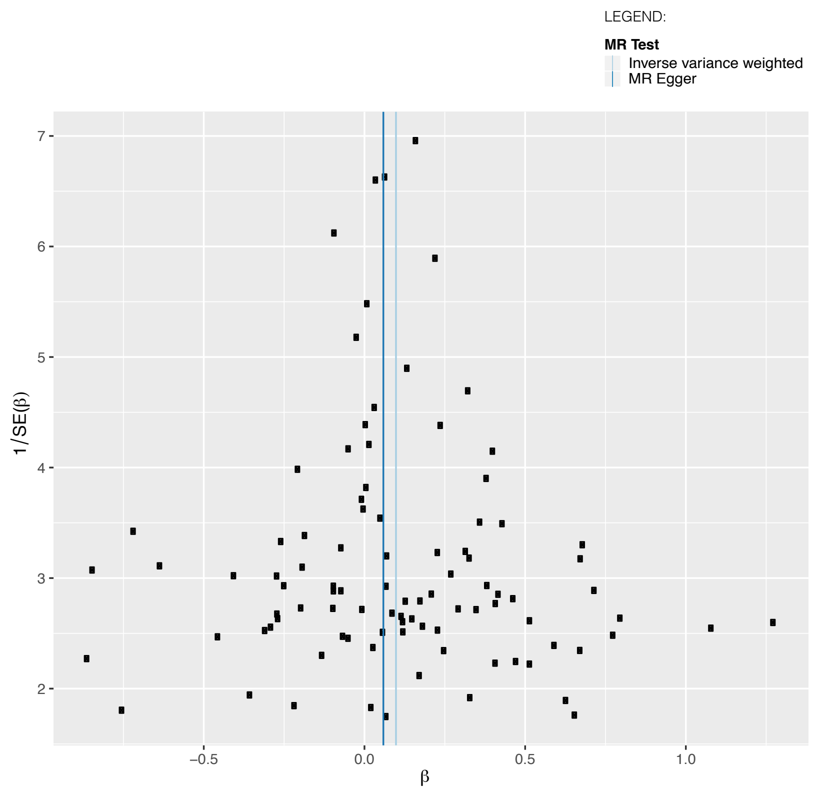

Figure S2. Leave-one-out plot for T2DM and breast cancer specific-survival showing the estimate effect by sequentially dropping one SNP at a time. Each black dot in the forest plot represents the MR results (IVW method) excluding that particular SNP. The result including all SNPs is shown in red at the bottom of the plot.

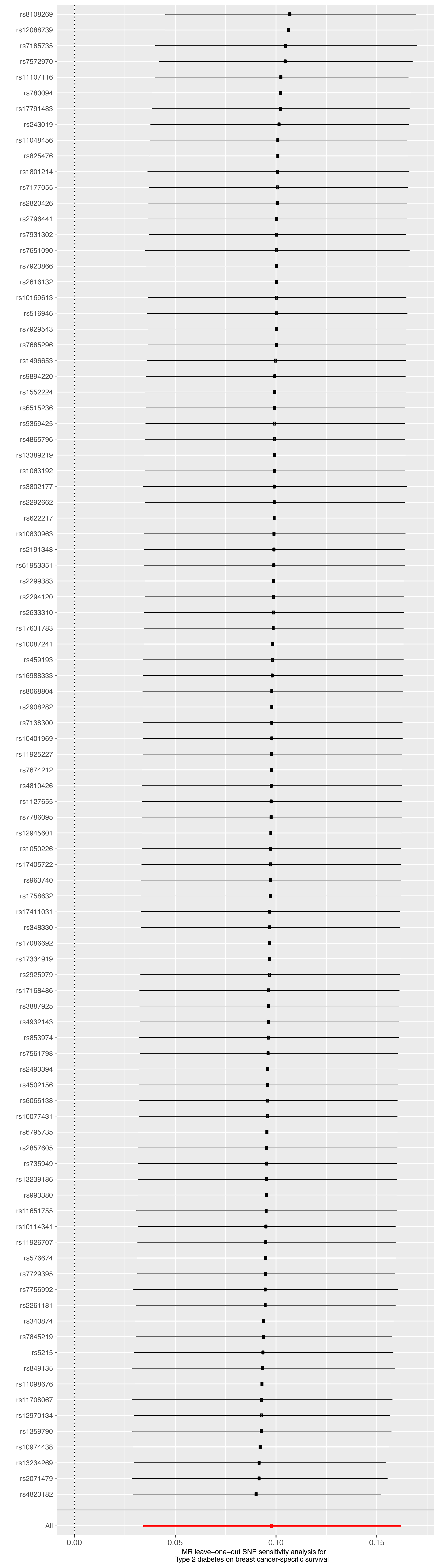

rs8108269
rs12088739
rs7185735
rs7572970
rs11107116
rs780094
rs17791483
rs243019
rs11048456
rs825476
rs1801214
rs7177055
rs2820426
rs2796441
rs7931302
rs7651090
rs7923866
rs2616132
rs10169613
rs516946
rs7929543
rs7685296
rs1496653
rs9894220
rs1552224
rs6515236
rs9369425
rs4865796
rs13389219
rs1063192
rs3802177
rs2292662
rs622217
rs10830963
rs2191348
rs61953351
rs2299383
rs2294120
rs2633310
rs17631783
rs10087241
rs459193
rs16988333
rs8068804
rs2908282
rs7138300
rs10401969
rs11925227
rs7674212
rs4810426
rs1127655
rs7786095
rs12945601
rs1050226
rs17405722
rs963740
rs1758632
rs17411031
rs348330
rs17086692
rs17334919
rs2925979
rs17168486
rs3887925
rs4932143
rs853974
rs7561798
rs2493394
rs4502156
rs6066138
rs10077431
rs6795735
rs2857605
rs735949
rs13239186
rs993380
rs11651755
rs10114341
rs11926707
rs576674
rs7729395
rs7756992
rs2261181
rs340874
rs7845219
rs5215
rs849135
rs11098676
rs11708067
rs12970134
rs1359790
rs10974438
rs13234269
rs2071479
rs4823182
All
0.00
0.05
0.10
0.15
MR leave−one−out SNP sensitivity analysis for
Type 2 diabetes on breast cancer-specific survival
