# Supplementary material for: Breast cancer risk factors and their effects on survival: a Mendelian randomisation study
Source: BMC Med. 2020 Nov 17;18:327. doi: 10.1186/s12916-020-01797-2 (PMC7670589; doi:10.1186/s12916-020-01797-2)
Supplement: Supplementary file 2 — Additional file 2: SNPs used in the analyses for the nine risk factors. The risk factor estimates (beta and standard error (SE)) and breast cancer-specific survival estimates for each SNP are included. Table S1. Alcohol consumption. Table S2. Body mass index. Table S3. Height. Table S4. Mammographic density. Table S5. Menarche. Table S6. Menopause. Table S7. Physical activity. Table S8. Smoking behaviour. Table S9. Type 2 diabetes mellitus. Table S10. Body mass index European-specific. Table S11. Type 2 diabetes mellitus replicate. [file 12916_2020_1797_MOESM2_ESM.pdf]

Additional file 2

SNPs used in the analyses for the nine risk factors. The risk factor estimates (beta and standard error (SE)) and breast cancer-specific survival estimates for each SNP are included.

Table S1. Alcohol consumption

						Alcohol GWAS			Breast cancer-specific: All			Breast cancer-specific: ER-positive			Breast cancer-specific: ER-negative		
SNP*	Chr	Pos	Effect allele	Other allele	EAF	Beta	SE	P-value	Beta	SE	P-value	Beta	SE	P-value	Beta	SE	P-value
rs141973904	4	100262242	T	C	0.017	-0.134	0.005	3.00E-139	0.045	0.005	4.89E-01	0.045	0.082	8.20E-02	0.152	0.130	2.43E-01
rs62250685	3	85457240	G	A	0.614	-0.016	0.002	1.00E-31	-0.006	0.002	7.53E-01	-0.020	0.023	2.28E-02	-0.011	0.036	7.51E-01
rs6951574	7	153489744	C	T	0.458	0.014	0.001	9.00E-23	0.012	0.001	5.19E-01	0.050	0.024	2.37E-02	-0.105	0.037	4.70E-03
rs4699680	4	99759132	A	G	0.957	0.031	0.003	6.00E-22	0.004	0.003	9.19E-01	-0.023	0.053	5.30E-02	-0.019	0.084	8.18E-01
rs281379	19	49214274	A	G	0.508	0.013	0.001	4.00E-21	-0.008	0.001	6.62E-01	0.007	0.022	2.21E-02	-0.019	0.035	5.91E-01
rs2668680	17	43659975	C	T	0.177	-0.017	0.002	4.00E-19	0.004	0.002	8.88E-01	0.033	0.032	3.22E-02	-0.087	0.051	8.70E-02
rs11604680	11	47457539	G	A	0.321	-0.012	0.001	4.00E-16	0.036	0.001	5.15E-02	0.020	0.024	2.41E-02	0.069	0.038	6.40E-02
rs17177078	16	24810681	T	C	0.063	-0.022	0.003	6.00E-16	-0.068	0.003	6.09E-02	-0.036	0.046	4.64E-02	-0.127	0.074	8.57E-02
rs13024996	2	144225215	A	C	0.364	-0.011	0.001	2.00E-15	-0.002	0.001	9.08E-01	-0.009	0.024	2.38E-02	-0.003	0.037	9.43E-01
rs11692435	2	98275354	A	G	0.085	0.019	0.003	3.00E-15	0.011	0.003	7.15E-01	0.019	0.037	3.73E-02	-0.004	0.059	9.39E-01
rs13094887	3	70968431	T	A	0.301	-0.011	0.001	3.00E-15	-0.021	0.001	2.51E-01	-0.023	0.024	2.41E-02	-0.016	0.038	6.80E-01
rs6982152	8	64779013	C	T	0.763	0.012	0.002	4.00E-15	-0.010	0.002	7.26E-01	-0.020	0.035	3.51E-02	-0.043	0.053	4.25E-01
rs7594223	2	225472985	C	T	0.382	-0.010	0.001	2.00E-14	0.025	0.001	1.66E-01	0.031	0.023	2.34E-02	0.022	0.037	5.44E-01
rs1940728	11	112852195	T	G	0.403	0.010	0.001	3.00E-14	-0.006	0.001	7.53E-01	-0.031	0.023	2.32E-02	0.045	0.037	2.24E-01
rs74980679	16	71779310	T	G	0.144	0.014	0.002	1.00E-13	0.050	0.002	3.97E-02	0.091	0.031	3.13E-02	-0.015	0.050	7.68E-01
rs11030084	11	27643725	T	C	0.184	-0.012	0.002	2.00E-13	-0.003	0.002	8.89E-01	-0.020	0.028	2.77E-02	0.037	0.043	3.91E-01
rs13032049	2	63581507	G	A	0.283	0.011	0.001	2.00E-13	0.029	0.001	1.25E-01	0.022	0.024	2.40E-02	0.039	0.038	3.13E-01
rs10236149	7	98977515	G	A	0.123	-0.015	0.002	4.00E-13	0.001	0.002	9.67E-01	0.051	0.032	3.23E-02	-0.128	0.053	1.59E-02
rs28680958	1	173848808	A	G	0.217	-0.011	0.002	7.00E-13	-0.027	0.002	1.97E-01	-0.026	0.027	2.70E-02	-0.018	0.042	6.70E-01
rs828867	2	74334462	A	G	0.545	0.010	0.001	8.00E-13	-0.015	0.001	5.30E-01	0.003	0.030	3.05E-02	-0.026	0.047	5.83E-01
rs2997084	1	66393732	G	C	0.433	-0.010	0.001	9.00E-13	0.022	0.001	2.27E-01	0.035	0.023	2.33E-02	0.033	0.037	3.73E-01
rs10978550	9	109345993	C	T	0.206	-0.012	0.002	2.00E-12	0.002	0.002	9.40E-01	-0.006	0.027	2.70E-02	-0.002	0.042	9.56E-01
rs58107686	1	33837334	A	C	0.328	-0.010	0.001	2.00E-12	0.002	0.001	8.95E-01	0.023	0.024	2.37E-02	-0.033	0.038	3.74E-01
rs66904764	1	71493639	C	T	0.278	0.010	0.001	3.00E-12	-0.003	0.001	8.62E-01	-0.009	0.025	2.52E-02	0.011	0.039	7.77E-01
rs10085696	7	69783020	G	A	0.186	-0.011	0.002	1.00E-11	0.015	0.002	5.98E-01	0.030	0.037	3.69E-02	0.033	0.057	5.61E-01
rs1123285	14	57274519	G	C	0.335	-0.009	0.001	2.00E-11	-0.018	0.001	3.12E-01	-0.019	0.023	2.34E-02	-0.032	0.037	3.87E-01
rs12466127	2	162815262	A	C	0.376	0.009	0.001	2.00E-11	0.001	0.001	9.66E-01	0.018	0.023	2.28E-02	-0.020	0.036	5.84E-01
rs823114	1	205719532	A	G	0.553	0.009	0.001	2.00E-11	-0.009	0.001	5.98E-01	0.000	0.022	2.19E-02	-0.013	0.034	7.04E-01
rs968050	6	98574560	T	C	0.483	0.009	0.001	2.00E-11	0.012	0.001	4.90E-01	-0.012	0.023	2.30E-02	0.038	0.036	2.89E-01
rs1814032	8	93005447	A	C	0.224	-0.011	0.002	3.00E-11	-0.004	0.002	8.50E-01	0.008	0.026	2.58E-02	0.013	0.040	7.54E-01
rs2872041	18	53064491	A	G	0.455	-0.009	0.001	4.00E-11	-0.011	0.001	5.18E-01	-0.018	0.022	2.23E-02	0.025	0.035	4.69E-01
rs72859280	2	147956293	T	G	0.036	0.024	0.004	4.00E-11	0.080	0.004	1.97E-01	0.085	0.081	8.12E-02	0.134	0.119	2.59E-01
rs17542254	11	113655696	G	A	0.275	0.010	0.001	5.00E-11	0.011	0.001	5.86E-01	0.026	0.025	2.52E-02	0.017	0.039	6.66E-01
rs2525565	17	29666588	C	T	0.619	0.009	0.001	6.00E-11	-0.009	0.001	6.25E-01	-0.005	0.022	2.25E-02	0.014	0.036	6.91E-01
rs10506274	12	81601464	T	G	0.484	-0.009	0.001	8.00E-11	-0.004	0.001	8.32E-01	0.001	0.022	2.23E-02	-0.013	0.035	7.11E-01
rs12907323	15	86796012	G	A	0.411	0.009	0.001	9.00E-11	-0.005	0.001	7.92E-01	0.011	0.022	2.23E-02	-0.041	0.035	2.45E-01
rs2472297	15	75027880	T	C	0.249	0.010	0.002	9.00E-11	0.011	0.002	5.95E-01	-0.008	0.027	2.74E-02	0.053	0.043	2.10E-01
rs1025143	15	47685059	C	T	0.357	0.009	0.001	1.00E-10	0.027	0.001	1.27E-01	0.034	0.023	2.29E-02	0.000	0.035	9.96E-01
rs4815364	20	25035711	A	G	0.616	0.009	0.001	2.00E-10	0.015	0.001	4.01E-01	0.030	0.023	2.34E-02	0.013	0.037	7.19E-01
rs4842786	12	92170791	A	G	0.584	-0.009	0.001	2.00E-10	-0.005	0.001	7.86E-01	-0.008	0.022	2.23E-02	0.019	0.035	5.85E-01
rs7956306	12	51834008	A	G	0.459	-0.008	0.001	2.00E-10	0.008	0.001	6.34E-01	0.008	0.022	2.19E-02	0.021	0.035	5.52E-01
rs28601761	8	126500031	G	C	0.420	0.008	0.001	3.00E-10	-0.010	0.001	5.94E-01	-0.004	0.023	2.33E-02	-0.041	0.036	2.50E-01
rs9607814	22	41946519	A	C	0.200	-0.011	0.002	3.00E-10	-0.020	0.002	3.54E-01	-0.035	0.028	2.79E-02	-0.001	0.044	9.74E-01
rs1714524	3	158273096	T	C	0.551	-0.008	0.001	6.00E-10	-0.010	0.001	5.67E-01	-0.002	0.022	2.19E-02	-0.017	0.034	6.21E-01
rs17665139	10	125093880	T	C	0.149	-0.011	0.002	7.00E-10	-0.020	0.002	3.96E-01	-0.022	0.031	3.07E-02	-0.002	0.048	9.73E-01
rs2132489	11	133647594	T	C	0.567	0.008	0.001	7.00E-10	-0.007	0.001	7.14E-01	-0.027	0.023	2.34E-02	0.083	0.037	2.41E-02
rs59367532	16	64532377	G	A	0.254	-0.009	0.002	8.00E-10	0.031	0.002	1.10E-01	0.038	0.025	2.53E-02	-0.017	0.041	6.70E-01
rs11125166	2	48462924	C	T	0.731	-0.009	0.002	9.00E-10	-0.018	0.002	3.70E-01	-0.020	0.025	2.52E-02	-0.014	0.040	7.26E-01
rs12572486	10	110545082	C	G	0.259	-0.009	0.002	9.00E-10	-0.015	0.002	4.57E-01	0.006	0.025	2.54E-02	0.001	0.040	9.79E-01
rs1937529	13	68107861	T	C	0.521	-0.008	0.001	1.00E-09	-0.014	0.001	4.20E-01	-0.009	0.022	2.25E-02	-0.024	0.036	4.93E-01
rs9893690	17	17753846	T	A	0.643	0.008	0.001	1.00E-09	0.003	0.001	8.76E-01	0.005	0.023	2.26E-02	0.026	0.035	4.57E-01
rs13068318	3	81321114	T	C	0.207	0.010	0.002	2.00E-09	0.007	0.002	8.07E-01	-0.021	0.036	3.65E-02	0.057	0.054	2.93E-01
rs1378835	3	117653416	G	T	0.546	0.008	0.001	2.00E-09	-0.015	0.001	3.71E-01	-0.016	0.022	2.19E-02	-0.023	0.035	5.18E-01
rs2299409	7	103812171	A	G	0.529	-0.008	0.001	2.00E-09	-0.048	0.001	4.90E-03	-0.044	0.022	2.20E-02	-0.075	0.034	2.71E-02
rs4092465	18	55080437	G	A	0.635	-0.008	0.001	2.00E-09	0.012	0.001	6.28E-01	0.005	0.031	3.10E-02	0.003	0.047	9.51E-01
rs6451575	5	19959057	T	A	0.573	-0.008	0.001	2.00E-09	-0.015	0.001	3.64E-01	-0.032	0.022	2.19E-02	0.010	0.034	7.59E-01
rs7581524	2	113234316	T	C	0.279	0.009	0.001	2.00E-09	-0.003	0.001	8.56E-01	-0.030	0.024	2.43E-02	0.018	0.038	6.34E-01
rs8097672	18	1839601	T	A	0.147	0.011	0.002	2.00E-09	0.007	0.002	7.73E-01	-0.007	0.031	3.06E-02	0.033	0.048	4.87E-01
rs10438820	17	78524597	T	C	0.702	0.009	0.001	3.00E-09	0.026	0.001	1.75E-01	0.020	0.025	2.50E-02	0.016	0.039	6.86E-01
rs13357015	5	80263403	A	G	0.645	0.008	0.001	3.00E-09	-0.002	0.001	8.99E-01	0.023	0.023	2.26E-02	-0.026	0.036	4.64E-01
rs4421172	6	92201719	T	C	0.627	-0.008	0.001	4.00E-09	-0.001	0.001	9.72E-01	0.009	0.023	2.27E-02	-0.028	0.036	4.44E-01
rs6671130	1	73851687	G	T	0.601	-0.008	0.001	4.00E-09	-0.035	0.001	4.43E-02	-0.045	0.022	2.23E-02	-0.020	0.035	5.74E-01
rs9838144	3	131576287	C	G	0.209	-0.010	0.002	4.00E-09	-0.001	0.002	9.68E-01	0.015	0.028	2.82E-02	0.028	0.044	5.26E-01
rs1286127	14	91390341	T	C	0.551	-0.008	0.001	5.00E-09	0.034	0.001	4.95E-02	0.040	0.022	2.20E-02	0.036	0.035	3.09E-01
rs55932213	9	108755622	G	A	0.736	0.009	0.002	5.00E-09	0.019	0.002	3.49E-01	0.015	0.026	2.56E-02	0.015	0.040	7.02E-01
rs10753661	1	165119792	A	G	0.684	-0.008	0.001	6.00E-09	0.016	0.001	4.13E-01	0.020	0.024	2.45E-02	-0.026	0.038	4.97E-01
rs13250583	8	20949917	T	C	0.213	-0.009	0.002	6.00E-09	0.015	0.002	4.84E-01	0.006	0.027	2.68E-02	0.059	0.042	1.64E-01
rs1426371	12	108629780	A	G	0.259	-0.009	0.002	6.00E-09	0.021	0.002	4.64E-01	0.004	0.037	3.73E-02	0.013	0.058	8.18E-01
rs2080870	5	60388313	T	A	0.747	-0.009	0.002	6.00E-09	0.040	0.002	4.35E-02	0.027	0.025	2.54E-02	0.046	0.040	2.47E-01
rs883323	5	166987744	T	C	0.624	-0.008	0.001	6.00E-09	-0.045	0.001	5.59E-02	-0.039	0.030	3.00E-02	-0.052	0.046	2.56E-01
rs3117334	6	29217514	T	G	0.501	0.008	0.001	7.00E-09	-0.023	0.001	1.74E-01	-0.065	0.022	2.19E-02	0.067	0.034	5.17E-02
rs55872084	5	155902003	T	G	0.235	0.009	0.002	9.00E-09	-0.006	0.002	7.57E-01	0.015	0.026	2.63E-02	0.000	0.042	9.93E-01
rs2279829	3	147106319	T	C	0.215	-0.009	0.002	1.00E-08	0.001	0.002	9.57E-01	0.001	0.028	2.81E-02	0.056	0.044	2.02E-01
rs8106871	19	42651600	T	C	0.118	0.012	0.002	1.00E-08	-0.024	0.002	3.90E-01	-0.001	0.036	3.56E-02	-0.034	0.056	5.44E-01
rs9325144	12	39018673	T	C	0.348	-0.008	0.001	1.00E-08	0.027	0.001	2.95E-01	0.037	0.033	3.28E-02	-0.023	0.051	6.43E-01
rs111908763	14	79932823	T	C	0.193	-0.009	0.002	2.00E-08	0.009	0.002	6.80E-01	-0.015	0.029	2.87E-02	0.017	0.044	6.97E-01
rs12651313	4	171086393	G	C	0.443	-0.007	0.001	2.00E-08	0.001	0.001	9.81E-01	-0.001	0.029	2.93E-02	0.021	0.044	6.35E-01
rs12655091	5	144412335	A	G	0.530	-0.007	0.001	2.00E-08	-0.037	0.001	3.31E-02	-0.035	0.023	2.26E-02	-0.069	0.035	5.03E-02
rs13148010	4	29694698	A	G	0.421	-0.008	0.001	2.00E-08	0.005	0.001	7.63E-01	0.020	0.023	2.33E-02	-0.011	0.036	7.62E-01
rs2297605	9	127255448	A	G	0.459	0.008	0.001	2.00E-08	0.000	0.001	9.95E-01	-0.018	0.022	2.22E-02	0.024	0.035	4.86E-01
rs2771046	9	23810888	A	C	0.188	0.009	0.002	2.00E-08	-0.017	0.002	5.78E-01	0.005	0.039	3.88E-02	-0.154	0.061	1.11E-02
rs339095	7	128406047	G	C	0.497	-0.007	0.001	2.00E-08	0.011	0.001	6.15E-01	-0.011	0.029	2.95E-02	-0.005	0.044	9.14E-01
rs6845321	4	113595869	T	C	0.306	0.008	0.001	2.00E-08	-0.013	0.001	6.08E-01	0.007	0.032	3.18E-02	-0.076	0.049	1.22E-01
rs7146019	14	99734984	G	A	0.578	0.008	0.001	2.00E-08	-0.026	0.001	2.78E-01	-0.008	0.031	3.08E-02	-0.114	0.046	1.43E-02
rs72630922	8	33662401	A	G	0.419	-0.008	0.001	2.00E-08	0.056	0.001	1.49E-02	0.065	0.029	2.94E-02	0.020	0.045	6.54E-01
rs2764771	16	20013793	A	G	0.307	0.008	0.001	3.00E-08	0.005	0.001	7.89E-01	-0.009	0.024	2.39E-02	0.040	0.037	2.84E-01
rs3761422	22	24826672	C	T	0.607	0.008	0.001	3.00E-08	0.020	0.001	3.81E-01	0.018	0.030	3.00E-02	0.013	0.045	7.71E-01
rs6777420	3	142002869	T	C	0.131	0.011	0.002	3.00E-08	-0.053	0.002	4.51E-02	-0.039	0.034	3.41E-02	-0.086	0.055	1.21E-01
rs9277636	6	33082512	G	A	0.153	0.011	0.002	3.00E-08	0.000	0.002	9.96E-01	0.020	0.030	2.99E-02	-0.049	0.047	3.00E-01
rs9613472	22	27972479	G	A	0.540	0.007	0.001	3.00E-08	0.015	0.001	3.90E-01	0.024	0.023	2.29E-02	0.000	0.036	9.90E-01
rs10166377	2	44312697	A	G	0.165	-0.010	0.002	4.00E-08	-0.028	0.002	2.50E-01	-0.051	0.032	3.16E-02	0.011	0.049	8.26E-01
rs10948615	6	51339421	C	A	0.306	-0.008	0.001	4.00E-08	0.002	0.001	8.99E-01	-0.005	0.024	2.40E-02	-0.030	0.038	4.29E-01
rs12185569	2	202875552	A	G	0.249	-0.008	0.002	4.00E-08	0.037	0.002	6.19E-02	0.041	0.025	2.54E-02	0.059	0.040	1.37E-01
rs13002088	2	178171918	A	G	0.784	0.009	0.002	4.00E-08	0.015	0.002	4.67E-01	0.009	0.027	2.68E-02	0.022	0.042	6.08E-01
rs147537311	2	206891281	T	A	0.089	-0.013	0.002	4.00E-08	0.034	0.002	2.80E-01	0.035	0.041	4.11E-02	0.057	0.063	3.69E-01
rs2300669	3	38541318	A	C	0.386	-0.007	0.001	4.00E-08	0.013	0.001	5.85E-01	0.001	0.030	3.02E-02	0.054	0.045	2.36E-01
rs61861298	10	125418287	C	A	0.233	-0.009	0.002	4.00E-08	-0.010	0.002	6.06E-01	0.005	0.026	2.60E-02	-0.023	0.042	5.86E-01
rs7618629	3	184023061	G	A	0.754	0.008	0.002	5.00E-08	0.007	0.002	7.22E-01	0.008	0.025	2.55E-02	0.009	0.040	8.17E-01

* Total number of SNPs after harmonization and filtering from originally 155 SNPs

SNP: Single Nucleotide Polymorphism
Chr: Chromosome
Pos: Position
EAF: Effect Allele Frequency
SE: Standard Error

Table S2. Body mass index

						BMI GWAS			Breast cancer-specific: All			Breast cancer-specific: ER-positive			Breast cancer-specific: ER-negative		
SNP*	Chr	Pos	Effect allele	Other allele	EAF	Beta	SE	P-value	Beta	SE	P-value	Beta	SE	P-value	Beta	SE	P-value
rs543874	1	177889480	A	G	0.813	-0.048	0.003	4.00E-52	-0.031	0.021	1.52E-01	-0.042	0.027	1.30E-01	0.028	0.044	5.22E-01
rs4715210	6	50897251	T	C	0.178	0.04	0.003	2.00E-34	0.012	0.023	5.99E-01	0.016	0.029	5.85E-01	0.027	0.045	5.48E-01
rs7647305	3	185834290	T	C	0.210	-0.036	0.003	7.00E-31	0.004	0.021	8.67E-01	0.004	0.027	8.76E-01	-0.012	0.043	7.73E-01
rs9563576	13	58670147	C	T	0.819	-0.023	0.002	6.00E-28	-0.001	0.022	9.76E-01	0.012	0.029	6.68E-01	0.003	0.045	9.47E-01
rs2112347	5	75015242	T	G	0.633	0.027	0.003	3.00E-26	0.018	0.018	2.96E-01	0.035	0.023	1.21E-01	-0.031	0.035	3.73E-01
rs7161194	14	101529005	G	A	0.674	0.018	0.002	1.00E-22	0.000	0.024	9.88E-01	-0.027	0.031	3.78E-01	0.005	0.047	9.13E-01
rs13107325	4	103188709	T	C	0.070	0.051	0.005	3.00E-21	-0.047	0.036	1.85E-01	-0.041	0.046	3.76E-01	-0.023	0.074	7.56E-01
rs10019997	4	137048599	T	C	0.408	0.015	0.002	7.00E-21	-0.002	0.018	8.92E-01	-0.020	0.023	3.82E-01	0.087	0.035	1.32E-02
rs10968576	9	28414339	A	G	0.688	-0.025	0.003	7.00E-21	-0.010	0.018	5.89E-01	-0.011	0.024	6.28E-01	0.029	0.037	4.38E-01
rs7144011	14	79940383	G	T	0.782	-0.029	0.003	3.00E-20	-0.005	0.021	8.11E-01	0.016	0.027	5.65E-01	-0.024	0.042	5.71E-01
rs7124681	11	47529947	C	A	0.593	-0.023	0.003	1.00E-19	-0.032	0.018	7.75E-02	-0.020	0.023	3.85E-01	-0.038	0.036	2.94E-01
rs12429545	13	54102206	G	A	0.874	-0.032	0.004	5.00E-18	-0.036	0.033	2.88E-01	0.005	0.043	9.08E-01	-0.077	0.064	2.30E-01
rs1436351	3	104617973	T	G	0.753	0.016	0.002	2.00E-17	-0.001	0.020	9.48E-01	-0.001	0.026	9.65E-01	-0.034	0.041	4.07E-01
rs4735692	8	76615663	A	G	0.579	0.024	0.003	7.00E-17	0.033	0.017	5.40E-02	0.044	0.022	4.88E-02	0.012	0.035	7.39E-01
rs4130548	1	78463868	T	C	0.645	-0.022	0.003	2.00E-16	0.000	0.019	9.82E-01	0.016	0.024	4.99E-01	-0.065	0.038	8.36E-02
rs6142096	20	32686658	A	G	0.536	0.014	0.002	2.00E-16	0.011	0.017	5.30E-01	-0.007	0.022	7.51E-01	0.031	0.035	3.70E-01
rs657452	1	49589847	A	G	0.389	0.021	0.003	5.00E-16	-0.015	0.018	4.05E-01	-0.009	0.023	6.94E-01	-0.031	0.036	3.97E-01
rs10742752	11	45438374	C	T	0.611	0.013	0.002	6.00E-16	-0.026	0.018	1.47E-01	-0.039	0.023	9.40E-02	0.032	0.037	3.81E-01
rs4833079	4	38654681	T	C	0.604	-0.013	0.002	8.00E-16	0.006	0.018	7.32E-01	-0.020	0.023	3.89E-01	0.061	0.036	9.15E-02
rs11081818	18	31251088	A	G	0.443	0.014	0.002	1.00E-15	0.002	0.017	9.28E-01	-0.033	0.022	1.39E-01	0.089	0.035	1.04E-02
rs2365389	3	61236462	C	T	0.590	0.02	0.003	3.00E-15	-0.021	0.017	2.21E-01	-0.003	0.022	8.98E-01	-0.039	0.035	2.64E-01
rs10132280	14	25928179	C	A	0.694	0.022	0.003	6.00E-15	0.012	0.019	5.23E-01	0.028	0.024	2.42E-01	-0.012	0.038	7.54E-01
rs1819844	12	68205604	A	G	0.179	-0.016	0.002	2.00E-14	0.011	0.022	6.12E-01	0.052	0.028	6.43E-02	-0.116	0.046	1.15E-02
rs7903146	10	114758349	T	C	0.289	-0.021	0.003	3.00E-14	0.012	0.018	5.21E-01	-0.009	0.024	7.06E-01	0.002	0.037	9.56E-01
rs7599312	2	213413231	G	A	0.715	0.021	0.003	4.00E-14	0.022	0.019	2.63E-01	0.017	0.025	4.97E-01	0.040	0.039	3.11E-01
rs9400239	6	108977663	T	C	0.298	-0.02	0.003	5.00E-14	0.010	0.019	5.95E-01	0.042	0.024	7.94E-02	-0.016	0.038	6.64E-01
rs7613875	3	49971514	A	C	0.529	0.02	0.003	7.00E-14	0.005	0.017	7.63E-01	0.026	0.022	2.45E-01	0.004	0.034	8.98E-01
rs118067556	10	63136165	C	T	0.971	-0.035	0.005	1.00E-13	0.034	0.055	5.34E-01	0.091	0.072	2.06E-01	-0.140	0.111	2.06E-01
rs2075650	19	45395619	A	G	0.856	0.027	0.004	1.00E-13	-0.021	0.025	4.18E-01	0.018	0.033	5.87E-01	-0.116	0.050	1.94E-02
rs887912	2	59302877	C	T	0.722	-0.021	0.003	1.00E-13	-0.002	0.019	9.19E-01	0.003	0.025	8.90E-01	-0.034	0.039	3.80E-01
rs11165643	1	96924097	C	T	0.422	-0.019	0.003	3.00E-13	0.026	0.023	2.58E-01	0.018	0.029	5.30E-01	-0.001	0.045	9.73E-01
rs3026101	17	5280440	T	C	0.696	-0.02	0.003	3.00E-13	-0.009	0.018	6.16E-01	-0.010	0.024	6.73E-01	-0.013	0.037	7.22E-01
rs12352785	9	6956850	A	C	0.316	-0.013	0.002	6.00E-13	-0.021	0.018	2.57E-01	-0.010	0.023	6.62E-01	-0.024	0.038	5.26E-01
rs1396141	2	41673745	T	C	0.660	0.013	0.002	7.00E-13	-0.002	0.018	8.92E-01	0.014	0.023	5.49E-01	-0.027	0.037	4.57E-01
rs10733682	9	129460914	G	A	0.522	-0.019	0.003	8.00E-13	-0.020	0.017	2.42E-01	-0.009	0.022	6.94E-01	-0.046	0.035	1.84E-01
rs11170468	12	39430048	A	C	0.756	-0.014	0.002	8.00E-13	-0.036	0.027	1.79E-01	-0.015	0.034	6.70E-01	-0.097	0.051	5.97E-02
rs2481665	1	62594677	T	C	0.552	0.018	0.003	1.00E-12	0.010	0.017	5.60E-01	0.021	0.022	3.30E-01	0.010	0.034	7.73E-01
rs1441264	13	79580919	G	A	0.398	-0.018	0.003	2.00E-12	-0.016	0.023	4.84E-01	-0.004	0.030	8.85E-01	-0.031	0.046	4.99E-01
rs16996700	20	50981945	T	C	0.741	0.02	0.003	2.00E-12	-0.026	0.026	3.16E-01	-0.045	0.033	1.68E-01	0.001	0.051	9.87E-01
rs2650492	16	28333411	G	A	0.688	-0.021	0.003	2.00E-12	-0.016	0.025	5.26E-01	-0.016	0.032	6.26E-01	-0.030	0.048	5.34E-01
rs815610	5	153517178	C	G	0.437	0.02	0.003	2.00E-12	0.010	0.017	5.64E-01	0.004	0.022	8.72E-01	0.021	0.035	5.53E-01
rs1167827	7	75163169	A	G	0.429	-0.019	0.003	3.00E-12	0.014	0.017	4.17E-01	0.000	0.022	9.85E-01	0.054	0.035	1.17E-01
rs28573110	17	34914365	G	A	0.458	-0.019	0.003	3.00E-12	-0.008	0.023	7.22E-01	-0.030	0.029	3.13E-01	0.070	0.044	1.12E-01
rs6569648	6	130349119	C	T	0.233	-0.013	0.002	5.00E-12	0.025	0.020	2.14E-01	0.017	0.026	5.05E-01	0.058	0.041	1.61E-01
rs6804842	3	25106437	A	G	0.417	-0.017	0.002	6.00E-12	-0.063	0.023	6.12E-03	-0.083	0.030	5.12E-03	-0.033	0.045	4.67E-01
rs6870983	5	87697533	C	T	0.781	0.02	0.003	9.00E-12	-0.007	0.020	7.22E-01	-0.013	0.026	6.19E-01	0.012	0.041	7.69E-01
rs11679338	2	181606895	C	T	0.317	-0.02	0.003	2.00E-11	0.008	0.018	6.72E-01	0.024	0.023	3.12E-01	-0.008	0.037	8.25E-01
rs977747	1	47684677	G	T	0.609	-0.017	0.003	2.00E-11	-0.012	0.018	5.04E-01	-0.026	0.023	2.41E-01	-0.033	0.036	3.56E-01
rs12939549	17	78611724	A	G	0.556	0.017	0.003	3.00E-11	-0.016	0.023	4.74E-01	0.006	0.030	8.42E-01	-0.019	0.045	6.80E-01
rs7899106	10	87410904	A	G	0.953	-0.039	0.006	3.00E-11	0.025	0.040	5.36E-01	-0.020	0.051	6.99E-01	0.130	0.081	1.08E-01
rs9581854	13	28017782	C	T	0.809	-0.024	0.004	3.00E-11	0.054	0.022	1.32E-02	0.068	0.028	1.61E-02	0.050	0.045	2.61E-01
rs11126666	2	26928811	G	A	0.716	-0.019	0.003	5.00E-11	0.018	0.021	3.85E-01	0.006	0.027	8.13E-01	-0.036	0.041	3.82E-01
rs2759315	15	81009646	A	C	0.438	0.018	0.003	5.00E-11	-0.009	0.017	5.95E-01	-0.024	0.022	2.71E-01	0.030	0.035	3.94E-01
rs2145272	20	6626218	A	G	0.660	0.018	0.003	6.00E-11	0.012	0.018	5.26E-01	0.024	0.024	3.18E-01	-0.016	0.037	6.73E-01
rs7204797	16	29968015	C	T	0.560	-0.019	0.003	6.00E-11	-0.023	0.017	1.84E-01	-0.013	0.022	5.62E-01	-0.053	0.035	1.29E-01
rs10920678	1	190239907	A	G	0.425	0.017	0.003	7.00E-11	0.028	0.023	2.18E-01	0.026	0.029	3.83E-01	0.031	0.045	4.91E-01
rs2836754	21	40291740	T	C	0.382	-0.017	0.003	7.00E-11	-0.015	0.018	4.06E-01	-0.002	0.023	9.44E-01	-0.048	0.036	1.76E-01
rs7143963	14	103304425	T	C	0.172	0.021	0.003	8.00E-11	0.013	0.022	5.59E-01	0.004	0.028	8.93E-01	0.015	0.045	7.39E-01
rs10460960	3	42308735	A	G	0.883	0.025	0.004	1.00E-10	-0.030	0.036	4.08E-01	-0.048	0.046	3.00E-01	0.008	0.071	9.12E-01
rs1928295	9	120378483	T	C	0.555	0.016	0.002	1.00E-10	-0.007	0.017	6.87E-01	0.038	0.022	9.00E-02	-0.120	0.035	5.63E-04
rs4755726	11	43642130	T	G	0.297	0.018	0.003	1.00E-10	-0.005	0.018	7.71E-01	0.003	0.024	9.07E-01	0.021	0.037	5.79E-01
rs1927790	13	96922191	T	C	0.581	-0.016	0.003	2.00E-10	-0.012	0.018	5.15E-01	-0.026	0.024	2.67E-01	0.021	0.037	5.67E-01
rs215607	7	32338337	G	A	0.219	0.024	0.004	2.00E-10	-0.014	0.021	5.27E-01	-0.030	0.028	2.80E-01	0.003	0.043	9.50E-01
rs6477694	9	111932342	C	T	0.363	0.016	0.003	2.00E-10	0.012	0.018	4.95E-01	0.037	0.023	1.04E-01	-0.009	0.036	8.03E-01
rs889398	16	69556715	C	T	0.569	0.016	0.003	2.00E-10	0.006	0.017	7.30E-01	0.012	0.022	5.88E-01	-0.009	0.035	7.99E-01
rs9364687	6	163817911	G	T	0.574	-0.01	0.002	2.00E-10	-0.028	0.023	2.16E-01	-0.023	0.030	4.44E-01	-0.071	0.044	1.07E-01
rs972540	2	207244783	A	G	0.735	-0.018	0.003	2.00E-10	0.007	0.020	7.50E-01	0.006	0.026	8.14E-01	-0.049	0.042	2.37E-01
rs11057405	12	122781897	A	G	0.104	-0.029	0.005	3.00E-10	0.006	0.039	8.82E-01	-0.061	0.052	2.36E-01	0.090	0.074	2.27E-01
rs2044148	2	24737833	G	A	0.773	0.02	0.003	3.00E-10	-0.012	0.022	5.77E-01	0.012	0.029	6.73E-01	0.009	0.044	8.46E-01
rs4820408	22	40604945	G	T	0.604	-0.016	0.003	3.00E-10	0.003	0.017	8.49E-01	0.007	0.022	7.37E-01	-0.007	0.035	8.37E-01
rs1816537	11	112968651	A	C	0.451	-0.018	0.003	4.00E-10	0.003	0.017	8.48E-01	0.005	0.022	8.11E-01	-0.008	0.036	8.15E-01
rs206936	6	34302869	A	G	0.801	-0.019	0.003	4.00E-10	0.012	0.021	5.65E-01	-0.002	0.027	9.45E-01	0.028	0.043	5.15E-01
rs2890652	2	142959931	T	C	0.812	-0.024	0.004	4.00E-10	0.007	0.023	7.66E-01	0.018	0.030	5.52E-01	-0.034	0.046	4.54E-01
rs4740619	9	15634326	C	T	0.440	-0.016	0.003	4.00E-10	-0.020	0.017	2.54E-01	-0.051	0.022	2.23E-02	0.026	0.035	4.53E-01
rs17016673	2	79497779	C	G	0.895	-0.028	0.005	5.00E-10	-0.064	0.027	1.74E-02	-0.049	0.035	1.59E-01	-0.079	0.055	1.47E-01
rs7580766	2	42939351	G	A	0.558	0.011	0.002	6.00E-10	-0.013	0.017	4.51E-01	-0.036	0.022	1.02E-01	0.029	0.034	3.96E-01
rs1561589	10	126695673	G	A	0.644	-0.017	0.003	7.00E-10	-0.012	0.018	5.11E-01	-0.035	0.023	1.20E-01	0.028	0.036	4.37E-01
rs17759796	22	22190163	A	C	0.132	0.014	0.002	7.00E-10	-0.061	0.024	1.18E-02	-0.075	0.031	1.71E-02	-0.033	0.049	5.01E-01
rs7239883	18	40147671	A	G	0.608	-0.016	0.003	7.00E-10	-0.024	0.018	1.68E-01	-0.014	0.023	5.36E-01	-0.056	0.036	1.18E-01
rs7164727	15	73093991	T	C	0.692	0.016	0.003	9.00E-10	-0.007	0.019	7.09E-01	0.020	0.024	4.02E-01	-0.029	0.038	4.45E-01
rs17203016	2	208255518	A	G	0.795	-0.02	0.003	1.00E-09	-0.024	0.022	2.57E-01	-0.046	0.028	1.02E-01	0.053	0.044	2.33E-01
rs11181001	12	41948196	G	A	0.532	-0.015	0.003	2.00E-09	0.010	0.017	5.67E-01	0.024	0.022	2.80E-01	0.003	0.035	9.27E-01
rs1801282	3	12393125	C	G	0.874	-0.023	0.004	2.00E-09	-0.020	0.026	4.54E-01	0.027	0.034	4.36E-01	-0.074	0.052	1.55E-01
rs34811474	4	25408838	A	G	0.220	-0.02	0.003	2.00E-09	-0.011	0.032	7.26E-01	0.000	0.041	9.97E-01	-0.014	0.064	8.27E-01
rs12961799	18	12904399	C	T	0.284	0.011	0.002	3.00E-09	-0.042	0.019	2.76E-02	-0.030	0.025	2.17E-01	-0.059	0.039	1.27E-01
rs4072096	2	227036603	A	C	0.502	-0.015	0.003	3.00E-09	-0.001	0.018	9.72E-01	-0.001	0.023	9.78E-01	0.002	0.036	9.66E-01
rs2245368	7	76608143	C	T	0.179	0.025	0.004	5.00E-09	-0.016	0.029	5.90E-01	-0.022	0.038	5.54E-01	-0.005	0.058	9.26E-01
rs2270204	9	131042734	T	G	0.760	-0.018	0.003	5.00E-09	0.048	0.026	6.67E-02	0.041	0.034	2.28E-01	0.030	0.052	5.68E-01
rs4238331	15	59012133	T	G	0.299	-0.011	0.002	5.00E-09	0.055	0.028	4.73E-02	0.019	0.036	5.95E-01	0.086	0.054	1.14E-01
rs12770228	10	21783634	G	A	0.663	-0.018	0.003	6.00E-09	-0.005	0.018	7.71E-01	-0.006	0.023	8.05E-01	0.015	0.036	6.77E-01
rs1808192	17	45794706	A	G	0.342	0.016	0.003	6.00E-09	-0.023	0.018	2.17E-01	-0.047	0.024	4.86E-02	0.068	0.036	6.12E-02
rs3184504	12	111884608	C	T	0.512	0.015	0.003	6.00E-09	0.009	0.017	6.04E-01	0.020	0.022	3.57E-01	0.050	0.034	1.47E-01
rs7181659	15	95267483	A	G	0.522	0.015	0.003	6.00E-09	-0.022	0.017	1.99E-01	-0.010	0.022	6.37E-01	-0.015	0.034	6.63E-01
rs2080454	16	49062590	C	A	0.389	0.015	0.003	8.00E-09	-0.001	0.018	9.77E-01	-0.010	0.023	6.73E-01	0.041	0.037	2.66E-01
rs513357	6	69558698	G	A	0.885	0.015	0.003	8.00E-09	0.036	0.027	1.89E-01	0.037	0.035	2.95E-01	-0.045	0.055	4.21E-01
rs1720825	3	138108083	G	A	0.811	-0.018	0.003	9.00E-09	0.028	0.029	3.31E-01	0.034	0.037	3.64E-01	-0.074	0.056	1.86E-01
rs2075171	2	58688907	G	A	0.766	0.017	0.003	1.00E-08	-0.001	0.021	9.52E-01	0.031	0.027	2.59E-01	-0.047	0.042	2.66E-01
rs3783890	14	93790276	T	C	0.813	0.018	0.003	1.00E-08	-0.033	0.022	1.22E-01	-0.060	0.028	2.86E-02	0.004	0.044	9.24E-01
rs6504108	17	46292923	C	T	0.282	0.016	0.003	1.00E-08	0.038	0.019	4.01E-02	0.029	0.024	2.22E-01	0.032	0.038	4.00E-01
rs10929925	2	6155557	A	C	0.454	-0.014	0.002	2.00E-08	0.026	0.018	1.62E-01	0.020	0.024	4.02E-01	0.041	0.036	2.56E-01
rs7191155	16	19800213	T	C	0.830	0.023	0.004	2.00E-08	-0.010	0.023	6.48E-01	0.014	0.030	6.35E-01	-0.077	0.045	8.59E-02
rs756717	16	72996162	A	G	0.399	-0.015	0.003	2.00E-08	0.007	0.018	7.00E-01	0.017	0.023	4.56E-01	0.019	0.037	6.09E-01
rs7570971	2	135837906	C	A	0.613	0.015	0.003	2.00E-08	0.026	0.033	4.28E-01	0.038	0.042	3.73E-01	0.018	0.069	7.91E-01
rs4986044	17	21261560	C	T	0.532	0.015	0.003	3.00E-08	-0.029	0.017	9.14E-02	-0.020	0.022	3.69E-01	-0.030	0.034	3.85E-01
rs876424	3	131637676	T	G	0.657	-0.017	0.003	3.00E-08	0.010	0.019	5.79E-01	0.010	0.024	6.79E-01	-0.040	0.038	2.96E-01
rs1460676	2	164567689	T	C	0.826	-0.018	0.003	4.00E-08	-0.010	0.023	6.75E-01	-0.029	0.029	3.13E-01	0.035	0.046	4.45E-01
rs492400	2	219349752	C	T	0.420	0.014	0.003	4.00E-08	-0.010	0.018	5.85E-01	-0.014	0.023	5.42E-01	0.025	0.036	4.98E-01
rs2185027	6	153381622	A	C	0.701	-0.015	0.003	5.00E-08	0.009	0.018	6.31E-01	-0.007	0.024	7.68E-01	0.067	0.038	7.45E-02

* Total number of SNPs after harmonization and filtering from originally 287 SNPs

(yellow)	SNPs removed. Overlapping between BMI and T2DM
(green)	SNPs removed. In linkage desequilibrium with SNPs in T2DM

SNP: Single Nucleotide Polymorphism
Chr: Chromosome
Pos: Position
EAF: Effect Allele Frequency
SE: Standard Error

Table S3. Height

						Height GWAS			Breast cancer-specific: All			Breast cancer-specific: ER-positive			Breast cancer-specific: ER-negative		
**SNP***	**Chr**	**Pos**	**Effect allele**	**Other allele**	**EAF**	**Beta**	**SE**	**P-value**	**Beta**	**SE**	**P-value**	**Beta**	**SE**	**P-value**	**Beta**	**SE**	**P-value**
rs724016	3	141105570	A	G	0.558	-0.078	0.003	3.00E-158	-0.003	0.017	8.38E-01	0.022	0.022	3.23E-01	-0.075	0.034	2.85E-02
rs143384	20	34025756	A	G	0.564	-0.075	0.030	1.00E-121	0.029	0.018	1.02E-01	0.029	0.023	2.05E-01	0.003	0.036	9.43E-01
rs42039	7	92244422	T	C	0.246	0.068	0.003	4.00E-88	-0.010	0.019	5.99E-01	-0.026	0.025	3.02E-01	-0.027	0.039	4.93E-01
rs1812175	4	145574844	A	G	0.162	-0.079	0.004	2.00E-86	-0.021	0.023	3.62E-01	-0.033	0.029	2.64E-01	-0.021	0.046	6.51E-01
rs7692995	4	17936634	T	C	0.844	0.074	0.004	1.00E-71	-0.014	0.023	5.37E-01	-0.004	0.030	8.95E-01	-0.001	0.046	9.88E-01
rs798497	7	2795957	A	G	0.701	0.057	0.003	2.00E-71	-0.005	0.018	7.66E-01	0.003	0.024	8.88E-01	-0.012	0.038	7.52E-01
rs3791679	2	56096892	A	G	0.763	0.06	0.004	2.00E-67	-0.007	0.020	7.18E-01	-0.012	0.026	6.53E-01	0.027	0.040	4.99E-01
rs4896582	6	142703877	A	G	0.304	-0.051	0.003	3.00E-55	0.020	0.018	2.68E-01	0.004	0.024	8.51E-01	0.059	0.037	1.10E-01
rs4369779	18	20735408	C	T	0.791	0.056	0.004	2.00E-53	-0.006	0.021	7.63E-01	0.019	0.027	4.83E-01	-0.043	0.043	3.11E-01
rs3814333	1	184007119	T	C	0.310	0.049	0.003	5.00E-51	0.006	0.018	7.56E-01	-0.007	0.024	7.63E-01	0.037	0.037	3.11E-01
rs12214804	6	34188866	T	C	0.917	-0.084	0.006	2.00E-49	-0.022	0.030	4.76E-01	-0.070	0.038	6.73E-02	0.037	0.063	5.53E-01
rs3825199	12	93976954	G	A	0.224	0.051	0.004	4.00E-49	-0.041	0.021	4.76E-02	-0.027	0.026	3.12E-01	-0.032	0.041	4.37E-01
rs17556750	4	82155568	A	C	0.304	0.046	0.003	8.00E-48	0.004	0.019	8.36E-01	-0.031	0.024	2.00E-01	0.077	0.038	4.11E-02
rs2257011	15	84266145	T	G	0.500	0.044	0.003	1.00E-47	-0.015	0.017	3.93E-01	0.009	0.022	6.91E-01	-0.050	0.035	1.52E-01
rs2079795	17	59496649	C	T	0.671	-0.045	0.003	2.00E-46	-0.033	0.018	6.58E-02	-0.059	0.023	1.09E-02	0.008	0.036	8.36E-01
rs552707	7	28205303	T	C	0.292	0.046	0.003	9.00E-46	0.014	0.019	4.48E-01	0.028	0.024	2.40E-01	-0.018	0.038	6.42E-01
rs1155939	6	126866133	A	C	0.495	0.042	0.003	1.00E-45	-0.011	0.017	5.08E-01	-0.004	0.022	8.66E-01	0.032	0.035	3.63E-01
rs314263	6	105392745	T	C	0.685	-0.043	0.003	1.00E-42	-0.027	0.018	1.34E-01	-0.027	0.023	2.41E-01	-0.004	0.036	9.21E-01
rs4733724	8	130723728	A	G	0.801	0.05	0.004	1.00E-41	-0.009	0.021	6.89E-01	-0.024	0.027	3.91E-01	-0.005	0.044	9.14E-01
rs3760318	17	29247715	A	G	0.367	-0.041	0.003	3.00E-41	-0.040	0.017	2.31E-02	-0.065	0.023	4.01E-03	-0.031	0.036	3.79E-01
rs2070776	17	62007498	G	A	0.666	0.042	0.003	6.00E-41	-0.043	0.018	1.36E-02	-0.021	0.023	3.53E-01	-0.101	0.036	4.65E-03
rs10958476	8	57095808	T	C	0.789	-0.051	0.004	2.00E-40	0.005	0.021	8.29E-01	-0.001	0.028	9.63E-01	-0.028	0.043	5.22E-01
rs7652177	3	171969077	C	G	0.497	-0.038	0.003	3.00E-39	0.019	0.018	2.88E-01	0.005	0.023	8.19E-01	0.030	0.036	4.03E-01
rs9392918	6	7708631	T	C	0.520	-0.038	0.003	4.00E-38	0.000	0.017	9.98E-01	0.004	0.022	8.62E-01	0.020	0.035	5.66E-01
rs1074683	20	32304653	C	G	0.773	0.044	0.003	8.00E-38	-0.020	0.020	3.14E-01	-0.021	0.025	4.19E-01	-0.042	0.040	2.90E-01
rs9428104	1	118855587	G	A	0.763	0.043	0.003	3.00E-36	-0.032	0.020	1.03E-01	-0.029	0.025	2.45E-01	-0.038	0.040	3.35E-01
rs2093210	14	60957279	T	C	0.609	-0.039	0.003	3.00E-35	-0.019	0.017	2.64E-01	0.003	0.022	9.03E-01	-0.048	0.035	1.68E-01
rs6694089	1	172083881	A	G	0.288	0.039	0.003	4.00E-33	0.009	0.019	6.47E-01	0.006	0.024	7.98E-01	0.035	0.038	3.53E-01
rs2278483	2	25040082	T	C	0.256	0.041	0.003	8.00E-33	0.016	0.020	4.35E-01	0.002	0.026	9.48E-01	0.003	0.041	9.32E-01
rs11049611	12	28600244	T	C	0.292	-0.038	0.003	3.00E-32	0.024	0.019	1.91E-01	0.009	0.024	6.97E-01	0.056	0.038	1.38E-01
rs1036821	8	135650483	A	G	0.297	-0.037	0.003	1.00E-30	0.006	0.019	7.49E-01	0.004	0.024	8.79E-01	0.007	0.037	8.56E-01
rs4448343	9	98266370	G	A	0.344	0.035	0.003	5.00E-30	0.011	0.018	5.41E-01	0.038	0.023	9.30E-02	-0.004	0.036	9.08E-01
rs4735677	8	78148191	A	T	0.712	-0.037	0.003	6.00E-30	0.026	0.019	1.61E-01	-0.001	0.024	9.51E-01	0.044	0.038	2.54E-01
rs720390	3	185548683	A	G	0.373	0.035	0.003	1.00E-29	-0.039	0.024	9.83E-02	-0.052	0.030	8.44E-02	-0.015	0.046	7.53E-01
rs7849585	9	139111870	T	G	0.333	0.036	0.003	1.00E-29	0.036	0.018	5.04E-02	0.037	0.023	1.16E-01	0.053	0.038	1.56E-01
rs17721822	20	6469596	A	G	0.353	-0.035	0.003	3.00E-29	0.014	0.018	4.43E-01	0.019	0.023	4.06E-01	0.019	0.036	6.02E-01
rs10748128	12	69827658	T	G	0.346	0.038	0.003	4.00E-29	-0.019	0.019	3.16E-01	-0.014	0.024	5.56E-01	-0.039	0.038	2.99E-01
rs11880992	19	2176403	A	G	0.420	0.033	0.003	7.00E-28	0.012	0.017	5.03E-01	0.052	0.022	2.04E-02	-0.053	0.035	1.28E-01
rs7466269	9	133464084	A	G	0.648	0.033	0.003	1.00E-27	0.024	0.019	1.99E-01	0.041	0.024	9.33E-02	0.005	0.038	8.89E-01
rs4986172	17	43216281	T	C	0.346	-0.034	0.003	8.00E-27	0.010	0.018	5.68E-01	0.034	0.023	1.39E-01	-0.023	0.036	5.33E-01
rs2687950	13	50718468	T	C	0.262	0.036	0.003	9.00E-27	0.008	0.020	7.05E-01	0.005	0.026	8.47E-01	0.011	0.040	7.91E-01
rs749052	2	232796610	T	C	0.947	0.067	0.006	2.00E-26	-0.004	0.050	9.31E-01	0.006	0.065	9.22E-01	-0.017	0.098	8.65E-01
rs991967	1	218615451	A	C	0.714	-0.034	0.003	2.00E-26	-0.015	0.019	4.28E-01	-0.006	0.024	7.94E-01	-0.037	0.037	3.17E-01
rs648831	6	80956208	T	C	0.527	0.031	0.003	3.00E-26	0.033	0.017	5.62E-02	0.059	0.022	7.76E-03	0.001	0.035	9.84E-01
rs12538407	7	23521316	A	G	0.605	0.032	0.003	4.00E-26	0.033	0.017	5.57E-02	0.045	0.022	4.60E-02	-0.001	0.035	9.77E-01
rs12209223	6	76164589	A	C	0.097	0.051	0.005	5.00E-25	-0.046	0.030	1.32E-01	-0.050	0.039	1.96E-01	-0.086	0.063	1.70E-01
rs6439168	3	129050943	A	G	0.213	-0.037	0.004	8.00E-25	0.034	0.028	2.13E-01	0.018	0.036	6.22E-01	0.043	0.054	4.28E-01
rs11684404	2	88924622	T	C	0.652	-0.032	0.003	9.00E-25	-0.024	0.018	1.89E-01	-0.005	0.023	8.36E-01	-0.051	0.036	1.55E-01
rs2974438	5	168250903	A	G	0.202	-0.037	0.004	4.00E-24	-0.023	0.022	3.07E-01	-0.002	0.028	9.33E-01	-0.056	0.045	2.12E-01
rs9993613	4	73476014	T	G	0.476	0.03	0.003	5.00E-24	0.014	0.023	5.29E-01	0.066	0.030	2.55E-02	-0.071	0.045	1.13E-01
rs11144688	9	78542286	A	G	0.125	-0.063	0.006	6.00E-24	-0.083	0.035	1.64E-02	-0.059	0.044	1.86E-01	-0.145	0.069	3.59E-02
rs6894139	5	88327782	T	G	0.544	0.03	0.003	6.00E-24	-0.008	0.017	6.41E-01	0.019	0.022	3.90E-01	-0.041	0.035	2.40E-01
rs2856321	12	11855773	A	G	0.650	-0.031	0.003	8.00E-24	-0.005	0.018	8.03E-01	0.015	0.024	5.20E-01	-0.023	0.038	5.36E-01
rs1401795	17	54839652	A	G	0.495	0.03	0.003	1.00E-23	-0.054	0.024	2.37E-02	-0.036	0.031	2.43E-01	-0.109	0.047	2.07E-02
rs7043114	9	95387983	T	C	0.560	-0.029	0.003	2.00E-22	-0.020	0.023	3.88E-01	-0.013	0.030	6.61E-01	-0.012	0.045	7.86E-01
rs12330322	3	72455355	T	C	0.224	-0.034	0.004	3.00E-22	0.005	0.021	7.96E-01	0.026	0.027	3.32E-01	-0.056	0.043	1.92E-01
rs1950500	14	24830850	T	C	0.291	0.031	0.003	3.00E-22	-0.004	0.020	8.37E-01	-0.007	0.025	7.87E-01	0.035	0.039	3.69E-01
rs7716219	5	54955071	C	T	0.701	-0.03	0.003	7.00E-22	0.006	0.018	7.42E-01	-0.012	0.024	6.24E-01	0.023	0.038	5.48E-01
rs10770705	12	20857467	A	C	0.324	0.03	0.003	2.00E-21	0.007	0.025	7.86E-01	-0.028	0.032	3.86E-01	0.050	0.047	2.94E-01
rs4803468	19	41922352	A	G	0.396	0.03	0.003	2.00E-21	0.004	0.018	8.27E-01	-0.014	0.024	5.54E-01	0.040	0.036	2.75E-01
rs10948222	6	45244415	T	C	0.596	-0.031	0.003	1.00E-20	-0.005	0.017	7.59E-01	-0.002	0.022	9.11E-01	0.004	0.035	8.98E-01
rs1681630	11	47969152	C	T	0.659	-0.029	0.003	2.00E-20	-0.018	0.024	4.46E-01	-0.009	0.031	7.59E-01	0.030	0.047	5.23E-01
rs2806561	1	23504795	A	G	0.565	0.027	0.003	2.00E-20	0.033	0.023	1.53E-01	0.042	0.029	1.50E-01	-0.026	0.045	5.60E-01
rs422421	5	176517326	T	C	0.216	-0.034	0.004	2.00E-20	0.035	0.021	9.25E-02	0.035	0.026	1.89E-01	0.018	0.042	6.70E-01
rs6600365	1	41556253	T	C	0.563	-0.027	0.003	2.00E-20	-0.040	0.017	1.81E-02	-0.062	0.022	4.61E-03	0.004	0.035	8.97E-01
rs7154721	14	92427348	T	C	0.571	0.027	0.003	5.00E-20	-0.013	0.017	4.60E-01	-0.020	0.022	3.56E-01	-0.028	0.035	4.12E-01
rs9217	17	7363088	C	T	0.354	0.028	0.003	5.00E-20	-0.004	0.018	8.10E-01	0.018	0.023	4.21E-01	-0.004	0.036	9.07E-01
rs2573625	15	100513158	T	C	0.675	0.029	0.003	6.00E-20	0.004	0.019	8.51E-01	0.012	0.025	6.29E-01	0.028	0.039	4.80E-01
rs7551732	1	89139041	A	T	0.594	0.027	0.003	6.00E-20	-0.013	0.018	4.55E-01	0.002	0.023	9.28E-01	-0.018	0.036	6.21E-01
rs862034	14	74990746	A	G	0.367	-0.028	0.003	6.00E-20	-0.022	0.018	2.33E-01	-0.002	0.023	9.24E-01	-0.071	0.037	5.44E-02
rs3767627	1	149938898	T	C	0.850	0.036	0.004	4.00E-19	0.016	0.024	5.01E-01	0.016	0.031	6.19E-01	-0.017	0.047	7.16E-01
rs4072910	19	8644031	C	G	0.483	-0.032	0.004	1.00E-18	0.005	0.023	8.41E-01	-0.031	0.030	3.02E-01	0.100	0.045	2.46E-02
rs4620037	5	170875097	A	C	0.792	0.032	0.004	1.00E-18	-0.032	0.022	1.40E-01	-0.052	0.028	6.59E-02	-0.030	0.044	5.03E-01
rs6974574	7	38110073	A	T	0.336	-0.03	0.003	1.00E-18	-0.029	0.018	1.20E-01	-0.029	0.024	2.22E-01	-0.053	0.037	1.52E-01
rs6714546	2	33361425	A	G	0.281	-0.03	0.003	2.00E-18	-0.016	0.019	3.96E-01	-0.026	0.024	2.75E-01	-0.024	0.038	5.31E-01
rs822531	7	148629759	T	C	0.794	0.036	0.004	2.00E-18	-0.019	0.028	4.96E-01	-0.012	0.036	7.40E-01	-0.068	0.054	2.09E-01
rs7731703	5	32694942	T	C	0.316	-0.03	0.003	4.00E-18	-0.002	0.019	9.12E-01	0.007	0.024	7.79E-01	-0.031	0.038	4.18E-01
rs2237886	11	2810731	T	C	0.102	0.043	0.005	5.00E-18	-0.013	0.029	6.52E-01	-0.014	0.037	6.97E-01	0.043	0.059	4.66E-01
rs13088462	3	51071713	C	T	0.055	0.059	0.007	8.00E-18	-0.019	0.038	6.26E-01	-0.027	0.049	5.86E-01	-0.031	0.076	6.80E-01
rs975210	15	70364352	A	G	0.172	0.035	0.004	1.00E-17	0.017	0.022	4.56E-01	0.022	0.029	4.50E-01	0.023	0.044	5.99E-01
rs994533	2	218284278	C	G	0.333	-0.027	0.003	1.00E-17	-0.003	0.018	8.81E-01	0.004	0.023	8.63E-01	-0.021	0.037	5.73E-01
rs6920372	6	109723939	A	G	0.407	-0.025	0.003	2.00E-17	0.020	0.017	2.32E-01	0.047	0.022	3.44E-02	-0.048	0.035	1.65E-01
rs318095	17	46974734	T	C	0.467	0.024	0.003	2.00E-16	0.003	0.018	8.67E-01	0.004	0.023	8.71E-01	-0.025	0.035	4.81E-01
rs897080	2	44774202	T	C	0.755	-0.028	0.003	2.00E-16	-0.022	0.020	2.61E-01	0.008	0.026	7.54E-01	-0.088	0.039	2.44E-02
rs17391694	1	78623626	T	C	0.116	0.043	0.005	4.00E-16	-0.004	0.036	9.07E-01	-0.040	0.047	3.94E-01	0.073	0.069	2.93E-01
rs16964211	15	51530495	A	G	0.055	-0.057	0.007	1.00E-15	0.008	0.038	8.34E-01	0.038	0.048	4.30E-01	-0.024	0.078	7.60E-01
rs1832871	6	158722034	A	G	0.337	0.025	0.003	2.00E-15	-0.022	0.019	2.26E-01	-0.011	0.024	6.31E-01	-0.058	0.038	1.23E-01
rs1562975	4	109408608	A	G	0.295	0.025	0.003	6.00E-15	-0.020	0.019	3.14E-01	-0.031	0.025	2.10E-01	0.024	0.039	5.44E-01
rs7112925	11	66826160	T	C	0.356	-0.024	0.003	6.00E-15	-0.005	0.018	7.56E-01	-0.003	0.023	8.87E-01	-0.073	0.036	4.20E-02
rs7319045	13	92024574	G	A	0.620	-0.024	0.003	8.00E-15	-0.025	0.018	1.57E-01	-0.047	0.023	3.83E-02	0.030	0.036	4.06E-01
rs1815314	10	80928793	A	G	0.411	-0.022	0.003	5.00E-14	0.017	0.017	3.17E-01	0.044	0.022	4.48E-02	0.017	0.035	6.14E-01
rs10401193	19	19591066	A	G	0.819	0.028	0.004	9.00E-14	-0.015	0.022	4.86E-01	0.014	0.029	6.31E-01	-0.022	0.044	6.18E-01
rs11835818	12	122494809	T	C	0.529	-0.022	0.003	2.00E-13	0.003	0.023	9.04E-01	-0.025	0.029	3.94E-01	0.081	0.045	6.87E-02
rs13177718	5	108113344	T	C	0.073	-0.043	0.006	3.00E-13	-0.021	0.033	5.22E-01	-0.034	0.043	4.33E-01	0.019	0.067	7.78E-01
rs10997979	10	69937192	G	A	0.497	0.021	0.003	4.00E-13	0.007	0.017	6.99E-01	0.015	0.022	5.04E-01	-0.026	0.034	4.44E-01
rs17807185	7	77308295	A	G	0.620	-0.022	0.003	4.00E-13	-0.019	0.023	4.25E-01	-0.044	0.030	1.40E-01	-0.007	0.046	8.71E-01
rs11152213	18	57852948	C	A	0.240	0.025	0.004	7.00E-13	0.050	0.020	1.19E-02	0.049	0.026	5.69E-02	0.088	0.040	2.64E-02
rs2238300	15	89851580	A	G	0.401	-0.021	0.003	2.00E-12	-0.030	0.017	8.53E-02	-0.032	0.022	1.58E-01	-0.065	0.035	6.63E-02
rs3739707	9	113792706	A	C	0.248	-0.024	0.004	4.00E-12	0.007	0.020	7.45E-01	0.031	0.026	2.33E-01	-0.090	0.042	3.17E-02
rs780094	2	27741237	T	C	0.375	-0.021	0.003	6.00E-12	0.014	0.017	4.15E-01	0.013	0.022	5.46E-01	0.038	0.035	2.87E-01
rs6446315	4	5035587	A	G	0.828	-0.028	0.004	4.00E-11	0.033	0.030	2.73E-01	0.053	0.039	1.75E-01	-0.008	0.058	8.97E-01
rs11750568	5	178535713	G	A	0.666	-0.02	0.003	6.00E-11	-0.050	0.019	8.85E-03	-0.035	0.025	1.59E-01	-0.091	0.038	1.82E-02
rs2378870	17	59638623	C	T	0.617	-0.02	0.003	4.00E-10	0.017	0.018	3.50E-01	0.015	0.023	4.97E-01	0.011	0.036	7.70E-01
rs6137287	20	21180259	T	C	0.279	0.02	0.003	4.00E-10	0.024	0.019	2.03E-01	0.033	0.025	1.84E-01	0.064	0.039	9.62E-02
rs2326458	16	84987679	A	C	0.732	-0.022	0.004	5.00E-10	0.015	0.019	4.33E-01	0.008	0.025	7.42E-01	0.060	0.039	1.23E-01
rs2120335	2	68495002	A	G	0.420	-0.019	0.003	8.00E-10	0.031	0.018	8.83E-02	0.048	0.024	4.29E-02	0.026	0.037	4.93E-01
rs4344931	2	241818527	C	A	0.714	0.02	0.003	8.00E-10	-0.002	0.020	9.09E-01	-0.019	0.025	4.54E-01	0.021	0.040	5.97E-01
rs1007358	7	46201355	A	G	0.781	-0.021	0.004	9.00E-10	-0.026	0.027	3.37E-01	-0.021	0.035	5.47E-01	-0.006	0.054	9.15E-01
rs6887276	5	127378294	C	G	0.542	-0.018	0.003	1.00E-09	0.008	0.018	6.66E-01	-0.003	0.023	8.97E-01	0.027	0.036	4.63E-01
rs17181956	2	218146080	T	C	0.107	-0.028	0.005	4.00E-09	0.027	0.027	3.16E-01	0.046	0.035	1.92E-01	0.060	0.056	2.84E-01
rs13113518	4	56399648	T	C	0.637	-0.018	0.003	8.00E-09	0.022	0.018	2.16E-01	0.032	0.023	1.61E-01	0.031	0.035	3.75E-01
rs6563199	13	81550449	T	C	0.345	0.018	0.003	1.00E-08	-0.038	0.018	3.88E-02	-0.030	0.023	1.96E-01	-0.050	0.037	1.73E-01

* Total number of SNPs after harmonization and filtering from originally 173 SNPs

SNP: Single Nucleotide Polymorphism
Chr: Chromosome
Pos: Position
EAF: Effect Allele Frequency
SE: Standard Error

Table S4. Mammographic density

						Mammograpic density GWAS			Breast cancer-specific: All			Breast cancer-specific: ER-positive			Breast cancer-specific: ER-negative		
**SNP***	**Chr**	**Pos**	**Effect allele**	**Other allele**	**EAF**	**Beta**	**SE**	**P-value**	**Beta**	**SE**	**P-value**	**Beta**	**SE**	**P-value**	**Beta**	**SE**	**P-value**
rs10995190	10	64278682	A	G	0.147	-0.240	0.031	1.00E-16	-0.005	0.024	8.44E-01	0.039	0.031	2.16E-01	-0.093	0.050	6.12E-02
rs10034692	4	75419787	A	G	0.719	-0.160	0.031	2.00E-10	-0.017	0.019	3.83E-01	-0.010	0.024	6.78E-01	-0.019	0.038	6.22E-01
rs703556	12	103011894	A	G	0.979	-0.410	0.082	4.00E-10	0.154	0.055	5.37E-03	0.097	0.071	1.70E-01	0.189	0.108	8.15E-02
rs12665607	6	151946629	A	T	0.085	-0.170	0.040	2.00E-08	0.080	0.029	6.31E-03	0.078	0.038	3.98E-02	0.037	0.057	5.11E-01
rs7289126	22	38628306	A	C	0.458	-0.110	0.020	3.00E-08	-0.022	0.017	1.97E-01	-0.018	0.022	4.19E-01	-0.065	0.034	5.79E-02

* Total number of SNPs after harmonization and filtering from originally 12 SNPs

SNP: Single Nucleotide Polymorphism
Chr: Chromosome
Pos: Position
EAF: Effect Allele Frequency
SE: Standard Error

Table S5. Menarche

						Menarche GWAS			Breast cancer-specific: All			Breast cancer-specific: ER-positive			Breast cancer-specific: ER-negative		
**SNP***	**Chr**	**Pos**	**Effect allele**	**Other allele**	**EAF**	**Beta**	**SE**	**P-value**	**Beta**	**SE**	**P-value**	**Beta**	**SE**	**P-value**	**Beta**	**SE**	**P-value**
rs7759938	6	105378954	C	T	0.316	0.120	0.005	8.00E-110	0.028	0.018	1.26E-01	0.028	0.023	2.32E-01	0.006	0.036	8.67E-01
rs6747380	2	56587749	A	G	0.179	0.070	0.007	6.00E-28	0.010	0.022	6.40E-01	0.010	0.029	7.38E-01	-0.078	0.046	8.82E-02
rs11715566	3	117562436	T	C	0.497	0.050	0.005	2.00E-27	0.009	0.017	5.84E-01	-0.012	0.022	5.71E-01	0.053	0.034	1.24E-01
rs466639	1	165394882	C	T	0.875	0.080	0.007	2.00E-24	-0.004	0.025	8.64E-01	0.019	0.033	5.61E-01	-0.056	0.050	2.71E-01
rs9635759	17	49613785	A	G	0.305	0.050	0.005	2.00E-24	-0.009	0.018	6.08E-01	-0.004	0.024	8.62E-01	0.030	0.037	4.17E-01
rs1079866	7	41470093	G	C	0.140	0.070	0.007	9.00E-24	0.007	0.025	7.84E-01	-0.019	0.032	5.56E-01	0.028	0.049	5.73E-01
rs1364063	16	69588572	C	T	0.429	0.050	0.005	6.00E-21	-0.007	0.017	6.94E-01	-0.013	0.022	5.58E-01	0.009	0.035	8.04E-01
rs13179411	5	133900513	T	G	0.158	0.060	0.007	3.00E-20	0.025	0.023	2.65E-01	0.008	0.029	7.92E-01	0.071	0.046	1.21E-01
rs1400974	2	199638690	A	G	0.643	0.050	0.005	8.00E-20	-0.012	0.018	4.89E-01	-0.008	0.023	7.18E-01	0.014	0.036	6.98E-01
rs2137289	18	44752125	A	G	0.589	0.050	0.005	8.00E-20	-0.020	0.017	2.40E-01	-0.028	0.022	2.01E-01	0.004	0.035	9.04E-01
rs8050136	16	53816275	C	A	0.605	0.040	0.005	2.00E-17	0.010	0.017	5.66E-01	0.007	0.022	7.45E-01	0.010	0.035	7.71E-01
rs9560113	13	112183348	G	A	0.281	0.050	0.006	2.00E-17	0.019	0.019	3.15E-01	0.003	0.024	9.00E-01	-0.008	0.038	8.31E-01
rs7821178	8	78093837	A	C	0.341	-0.040	0.005	7.00E-17	-0.023	0.018	1.92E-01	0.008	0.023	7.42E-01	-0.046	0.037	2.10E-01
rs7647973	3	49510931	A	G	0.273	0.050	0.006	1.00E-16	-0.004	0.019	8.24E-01	0.012	0.025	6.20E-01	0.031	0.038	4.19E-01
rs1254337	14	60920525	T	A	0.296	0.040	0.005	2.00E-16	0.023	0.018	2.18E-01	0.010	0.024	6.65E-01	0.049	0.037	1.87E-01
rs7642134	3	86916882	G	A	0.615	0.040	0.005	3.00E-16	0.011	0.017	5.27E-01	0.012	0.022	6.03E-01	-0.018	0.035	6.03E-01
rs9321659	6	100116092	A	G	0.124	0.060	0.008	3.00E-16	-0.016	0.026	5.43E-01	0.011	0.033	7.44E-01	-0.059	0.052	2.53E-01
rs246185	16	14395432	C	T	0.332	0.040	0.006	7.00E-16	0.006	0.018	7.23E-01	0.024	0.023	3.08E-01	-0.039	0.036	2.84E-01
rs543874	1	177889480	A	G	0.813	0.050	0.006	1.00E-15	-0.031	0.021	1.52E-01	-0.042	0.027	1.30E-01	0.028	0.044	5.22E-01
rs10144321	14	100882405	A	G	0.758	0.040	0.006	9.00E-15	0.002	0.020	9.36E-01	-0.005	0.025	8.47E-01	0.023	0.040	5.70E-01
rs10895140	11	101436721	G	A	0.656	0.040	0.005	7.00E-14	0.011	0.018	5.50E-01	0.028	0.023	2.15E-01	-0.021	0.036	5.56E-01
rs17171818	5	137725003	C	T	0.787	0.040	0.006	9.00E-14	0.000	0.020	9.85E-01	0.018	0.026	4.90E-01	-0.018	0.041	6.62E-01
rs7828501	8	4560081	G	A	0.446	0.040	0.005	1.00E-13	0.014	0.017	4.24E-01	-0.009	0.022	6.65E-01	0.025	0.034	4.59E-01
rs852069	20	17122593	G	A	0.639	0.040	0.005	1.00E-13	0.001	0.018	9.35E-01	0.009	0.023	6.97E-01	-0.014	0.036	6.94E-01
rs3743266	15	60781513	T	C	0.700	0.040	0.005	2.00E-13	-0.009	0.019	6.38E-01	-0.018	0.024	4.62E-01	0.071	0.038	6.39E-02
rs10938397	4	45182527	A	G	0.570	0.040	0.005	4.00E-13	-0.027	0.017	1.15E-01	-0.018	0.022	4.02E-01	-0.095	0.035	6.30E-03
rs3733631	4	104641103	C	G	0.154	0.050	0.007	5.00E-13	-0.008	0.023	7.44E-01	-0.015	0.030	6.19E-01	-0.065	0.047	1.67E-01
rs4756059	11	46150619	T	C	0.924	0.070	0.010	5.00E-13	-0.063	0.032	4.79E-02	-0.076	0.041	6.38E-02	-0.015	0.066	8.18E-01
rs4895808	6	126781434	T	C	0.460	-0.030	0.005	5.00E-13	-0.008	0.017	6.22E-01	-0.004	0.022	8.36E-01	0.041	0.035	2.33E-01
rs6758290	2	105864826	T	C	0.527	0.040	0.005	7.00E-13	0.009	0.017	5.95E-01	0.019	0.022	3.98E-01	0.005	0.034	8.79E-01
rs2479724	6	41890982	T	C	0.457	0.030	0.005	1.00E-12	0.021	0.017	2.13E-01	-0.004	0.022	8.61E-01	0.081	0.035	1.97E-02
rs988913	6	54756308	C	T	0.657	0.040	0.005	1.00E-12	0.030	0.018	8.93E-02	0.008	0.023	7.23E-01	0.086	0.036	1.72E-02
rs1874984	10	1731871	C	G	0.476	0.040	0.005	2.00E-12	-0.006	0.017	7.29E-01	0.020	0.022	3.73E-01	-0.049	0.035	1.63E-01
rs2063730	11	78048524	C	A	0.195	0.050	0.007	2.00E-12	0.032	0.022	1.37E-01	0.031	0.028	2.66E-01	0.003	0.044	9.37E-01
rs6770162	3	24711013	A	G	0.504	0.040	0.005	2.00E-12	-0.006	0.017	7.43E-01	0.022	0.022	3.31E-01	-0.052	0.036	1.41E-01
rs12915845	15	89042467	C	T	0.586	0.030	0.005	3.00E-12	-0.029	0.017	8.96E-02	-0.037	0.022	9.08E-02	0.005	0.035	8.81E-01
rs939317	3	184045799	G	A	0.749	0.040	0.006	3.00E-12	0.008	0.020	6.80E-01	0.007	0.025	7.97E-01	0.016	0.040	6.86E-01
rs1469039	8	140651779	A	G	0.175	0.050	0.007	4.00E-12	-0.015	0.022	5.08E-01	-0.008	0.029	7.85E-01	-0.071	0.045	1.20E-01
rs6964833	7	74101909	T	C	0.741	0.040	0.006	5.00E-12	0.008	0.019	6.66E-01	0.015	0.025	5.46E-01	0.013	0.039	7.41E-01
rs9475752	6	56780741	C	T	0.806	0.040	0.006	8.00E-12	0.009	0.021	6.67E-01	0.024	0.027	3.80E-01	-0.009	0.043	8.30E-01
rs9647570	5	167370263	G	T	0.139	0.050	0.007	1.00E-11	-0.003	0.025	9.20E-01	-0.023	0.033	4.91E-01	0.004	0.050	9.41E-01
rs1038903	4	28752054	T	C	0.728	0.040	0.006	2.00E-11	0.002	0.019	8.98E-01	-0.004	0.025	8.66E-01	-0.016	0.039	6.75E-01
rs11792861	9	111809295	A	C	0.707	0.040	0.005	2.00E-11	-0.019	0.019	3.18E-01	-0.034	0.024	1.57E-01	0.006	0.038	8.84E-01
rs6563739	13	40239785	G	T	0.349	0.030	0.005	2.00E-11	0.020	0.018	2.65E-01	0.033	0.023	1.58E-01	-0.005	0.036	8.80E-01
rs900400	3	156798775	T	C	0.582	0.030	0.005	2.00E-11	0.008	0.017	6.44E-01	-0.014	0.022	5.44E-01	0.071	0.035	4.39E-02
rs7103411	11	27700125	C	T	0.221	0.040	0.006	3.00E-11	0.000	0.021	9.85E-01	-0.010	0.027	7.11E-01	0.025	0.041	5.52E-01
rs11767400	7	122160742	A	C	0.295	0.040	0.006	4.00E-11	0.005	0.019	8.05E-01	0.041	0.024	8.91E-02	-0.021	0.038	5.90E-01
rs244293	17	53230722	G	A	0.601	0.030	0.005	4.00E-11	0.007	0.017	6.67E-01	-0.007	0.022	7.61E-01	0.005	0.035	8.96E-01
rs12148769	15	24152094	G	A	0.910	0.050	0.008	5.00E-11	0.004	0.029	8.85E-01	-0.015	0.037	6.78E-01	0.011	0.059	8.56E-01
rs11215400	11	115052635	C	A	0.248	0.040	0.006	7.00E-11	-0.024	0.020	2.33E-01	-0.012	0.026	6.47E-01	-0.022	0.041	5.92E-01
rs13135934	4	95207688	C	G	0.406	0.030	0.005	1.00E-10	-0.023	0.017	1.77E-01	-0.047	0.022	3.43E-02	0.008	0.035	8.17E-01
rs2687729	3	127895226	G	A	0.269	0.040	0.006	1.00E-10	0.010	0.019	5.99E-01	0.012	0.025	6.31E-01	-0.015	0.038	6.96E-01
rs12571664	10	121708929	T	C	0.797	0.040	0.006	3.00E-10	0.000	0.022	9.89E-01	-0.013	0.028	6.29E-01	0.054	0.044	2.17E-01
rs1324913	13	74635588	G	T	0.663	0.030	0.005	3.00E-10	0.034	0.018	6.20E-02	0.046	0.023	4.60E-02	0.021	0.037	5.70E-01
rs16896742	6	29922740	G	A	0.375	0.040	0.006	3.00E-10	0.024	0.017	1.67E-01	0.039	0.022	7.79E-02	-0.008	0.035	8.25E-01
rs251130	5	110859797	G	A	0.728	0.040	0.006	3.00E-10	0.009	0.020	6.38E-01	0.007	0.025	7.69E-01	0.018	0.040	6.49E-01
rs12472911	2	142228509	T	C	0.775	-0.040	0.006	7.00E-10	0.025	0.021	2.39E-01	0.020	0.027	4.60E-01	-0.014	0.042	7.43E-01
rs13067731	3	136989991	T	C	0.172	0.040	0.007	1.00E-09	-0.018	0.023	4.24E-01	-0.042	0.030	1.61E-01	0.049	0.045	2.82E-01
rs6933660	6	151803754	C	A	0.690	0.030	0.005	1.00E-09	0.008	0.018	6.50E-01	0.008	0.023	7.24E-01	-0.013	0.037	7.26E-01
rs11165924	1	98375448	A	G	0.674	0.030	0.006	2.00E-09	-0.028	0.018	1.14E-01	-0.023	0.023	3.24E-01	-0.036	0.036	3.24E-01
rs2274465	1	44121557	C	G	0.649	0.030	0.005	2.00E-09	0.041	0.018	2.08E-02	0.047	0.023	4.00E-02	-0.011	0.036	7.53E-01
rs2688325	8	3767210	T	C	0.291	0.030	0.006	2.00E-09	-0.008	0.019	6.58E-01	-0.012	0.024	6.28E-01	0.034	0.037	3.59E-01
rs6555855	5	168749737	G	A	0.220	0.040	0.006	2.00E-09	-0.031	0.021	1.38E-01	-0.035	0.027	1.86E-01	0.006	0.042	8.92E-01
rs7853970	9	86715566	T	C	0.475	0.030	0.005	2.00E-09	0.005	0.017	7.68E-01	0.004	0.022	8.61E-01	0.011	0.035	7.60E-01
rs17236969	2	156752459	T	C	0.145	0.050	0.008	3.00E-09	0.004	0.024	8.70E-01	0.003	0.031	9.31E-01	-0.076	0.051	1.36E-01
rs1532331	5	43116830	G	T	0.306	0.030	0.005	4.00E-09	-0.009	0.018	6.08E-01	0.002	0.024	9.22E-01	-0.014	0.038	7.10E-01
rs9447700	6	77168086	C	T	0.712	0.030	0.005	6.00E-09	0.043	0.019	2.29E-02	0.050	0.024	3.90E-02	0.074	0.038	5.07E-02
rs12446632	16	19935389	A	G	0.145	0.040	0.007	1.00E-08	0.002	0.024	9.51E-01	-0.027	0.032	3.97E-01	0.080	0.048	9.59E-02
rs16918254	8	53769213	A	G	0.930	0.050	0.009	1.00E-08	0.044	0.033	1.82E-01	0.050	0.042	2.36E-01	0.024	0.066	7.20E-01
rs4875053	8	144872411	G	C	0.440	0.030	0.006	1.00E-08	0.010	0.018	5.92E-01	-0.003	0.024	9.07E-01	0.003	0.037	9.29E-01
rs652260	19	7900562	T	C	0.532	0.030	0.005	1.00E-08	-0.004	0.023	8.60E-01	0.015	0.029	5.95E-01	-0.046	0.045	3.05E-01
rs7955374	12	47880149	T	C	0.123	0.040	0.008	1.00E-08	0.024	0.026	3.57E-01	0.017	0.033	6.05E-01	0.079	0.052	1.27E-01
rs10816359	9	108757670	T	G	0.833	0.040	0.008	2.00E-08	0.041	0.024	8.33E-02	0.054	0.030	7.41E-02	0.035	0.048	4.66E-01
rs951366	1	205685352	T	C	0.619	0.030	0.005	2.00E-08	-0.010	0.017	5.77E-01	0.005	0.022	8.36E-01	-0.016	0.035	6.58E-01
rs13053505	22	39245618	T	G	0.177	-0.040	0.007	3.00E-08	0.008	0.022	7.22E-01	-0.030	0.029	2.96E-01	0.012	0.045	7.97E-01
rs16918636	11	29124182	T	C	0.777	0.030	0.006	3.00E-08	0.031	0.021	1.38E-01	0.035	0.027	1.97E-01	0.066	0.043	1.18E-01
rs10789181	1	65816567	A	G	0.391	0.030	0.005	4.00E-08	0.005	0.018	7.86E-01	0.010	0.023	6.59E-01	-0.035	0.036	3.37E-01
rs17086188	5	95845854	A	G	0.973	0.070	0.013	4.00E-08	-0.031	0.045	4.95E-01	-0.009	0.057	8.81E-01	-0.044	0.093	6.36E-01
rs1958560	14	66036795	A	G	0.592	0.030	0.005	4.00E-08	-0.012	0.017	4.99E-01	-0.007	0.022	7.40E-01	-0.023	0.035	5.10E-01
rs6009583	22	49677646	T	C	0.273	-0.030	0.006	5.00E-08	0.012	0.020	5.65E-01	-0.018	0.026	5.02E-01	0.079	0.041	5.38E-02
rs6427782	1	199798339	A	G	0.521	0.030	0.005	5.00E-08	-0.001	0.017	9.31E-01	-0.005	0.022	8.26E-01	0.024	0.035	4.92E-01
rs7701886	5	153547409	A	G	0.559	0.030	0.005	5.00E-08	-0.008	0.017	6.63E-01	-0.002	0.022	9.34E-01	-0.017	0.035	6.23E-01

* Total number of SNPs after harmonization and filtering from originally 122 SNPs

SNP: Single Nucleotide Polymorphism
Chr: Chromosome
Pos: Position
EAF: Effect Allele Frequency
SE: Standard Error

Table S6. Menopause

						Menopause GWAS			Breast cancer-specific: All			Breast cancer-specific: ER-positive			Breast cancer-specific: ER-negative		
**SNP***	**Chr**	**Pos**	**Effect allele**	**Other allele**	**EAF**	**Beta**	**SE**	**P-value**	**Beta**	**SE**	**P-value**	**Beta**	**SE**	**P-value**	**Beta**	**SE**	**P-value**
rs11668344	19	55833664	G	A	0.349	-0.410	0.020	6.00E-85	0.012	0.018	4.89E-01	0.002	0.023	9.47E-01	-0.006	0.035	8.56E-01
rs4693089	4	84373622	A	G	0.508	-0.200	0.020	9.00E-23	0.000	0.017	9.78E-01	-0.001	0.022	9.57E-01	0.000	0.034	9.90E-01
rs2720044	8	37980587	A	C	0.838	-0.290	0.031	7.00E-22	-0.014	0.023	5.32E-01	0.016	0.029	5.91E-01	-0.052	0.046	2.56E-01
rs2277339	12	57146069	G	T	0.097	-0.310	0.031	2.00E-19	-0.025	0.028	3.81E-01	-0.036	0.037	3.28E-01	-0.046	0.058	4.29E-01
rs930036	2	171941018	A	G	0.361	-0.190	0.020	3.00E-19	0.011	0.017	5.36E-01	0.006	0.022	7.85E-01	0.032	0.035	3.71E-01
rs6856693	4	185748806	A	G	0.574	-0.160	0.020	1.00E-16	-0.008	0.017	6.56E-01	0.002	0.022	9.31E-01	-0.012	0.035	7.36E-01
rs4886238	13	61113739	A	G	0.341	0.180	0.020	3.00E-16	0.004	0.018	8.41E-01	0.017	0.023	4.49E-01	-0.015	0.036	6.74E-01
rs704795	2	27716494	A	G	0.405	-0.160	0.020	2.00E-15	-0.019	0.017	2.86E-01	-0.016	0.022	4.70E-01	-0.042	0.036	2.39E-01
rs707938	6	31729359	G	A	0.311	-0.170	0.020	7.00E-15	0.021	0.018	2.43E-01	0.028	0.023	2.34E-01	0.013	0.036	7.18E-01
rs2236918	1	242017826	C	G	0.441	-0.150	0.020	8.00E-14	-0.018	0.017	2.86E-01	-0.014	0.022	5.19E-01	-0.029	0.035	4.13E-01
rs11031006	11	30226528	G	A	0.858	-0.220	0.031	9.00E-14	0.018	0.024	4.52E-01	0.004	0.031	8.90E-01	0.043	0.049	3.87E-01
rs763121	22	38879940	G	A	0.344	-0.160	0.020	2.00E-13	-0.033	0.018	6.57E-02	-0.050	0.023	3.00E-02	-0.016	0.036	6.56E-01
rs2241584	5	175956177	A	G	0.385	-0.140	0.020	2.00E-11	0.010	0.017	5.58E-01	-0.013	0.022	5.76E-01	0.057	0.035	1.06E-01
rs1800932	2	48018081	A	G	0.829	-0.170	0.031	3.00E-11	-0.014	0.022	5.24E-01	0.005	0.028	8.61E-01	-0.050	0.043	2.44E-01
rs1799949	17	41245466	G	A	0.674	-0.140	0.020	8.00E-11	0.012	0.018	4.87E-01	0.030	0.023	2.05E-01	-0.030	0.036	3.99E-01
rs1411478	1	180962282	A	G	0.411	-0.130	0.020	1.00E-10	-0.006	0.017	7.20E-01	-0.023	0.022	3.10E-01	0.028	0.035	4.20E-01
rs9796	15	41271447	T	A	0.439	-0.130	0.020	1.00E-10	-0.032	0.017	6.86E-02	-0.036	0.022	1.09E-01	-0.043	0.035	2.21E-01
rs1713460	14	20933615	G	A	0.303	-0.140	0.020	2.00E-10	0.005	0.018	7.98E-01	-0.007	0.024	7.76E-01	0.033	0.037	3.83E-01
rs2236553	20	61289743	C	T	0.227	-0.160	0.031	6.00E-10	0.034	0.031	2.79E-01	0.041	0.040	3.06E-01	0.062	0.068	3.58E-01
rs2230365	6	31525448	C	T	0.849	-0.170	0.031	8.00E-10	-0.063	0.023	5.80E-03	-0.076	0.029	9.79E-03	-0.065	0.046	1.59E-01
rs2941505	17	37832704	A	G	0.320	-0.130	0.020	2.00E-09	-0.011	0.018	5.60E-01	0.002	0.034	9.52E-01	0.079	0.055	1.50E-01
rs8070740	17	5331896	A	G	0.754	-0.150	0.020	2.00E-09	-0.010	0.019	5.99E-01	-0.004	0.023	8.75E-01	0.012	0.036	7.47E-01
rs16858210	3	183624010	G	A	0.752	-0.140	0.020	3.00E-09	-0.028	0.019	1.50E-01	-0.032	0.025	1.94E-01	0.007	0.040	8.56E-01
rs10957156	8	61629401	A	G	0.762	-0.140	0.020	5.00E-09	0.028	0.020	1.55E-01	0.019	0.026	4.55E-01	0.031	0.041	4.52E-01
rs451417	20	5941999	A	C	0.124	-0.200	0.031	5.00E-09	-0.006	0.026	8.07E-01	-0.023	0.033	4.96E-01	0.012	0.052	8.13E-01
rs5762534	22	28633571	T	C	0.840	-0.160	0.031	6.00E-09	-0.015	0.023	5.08E-01	-0.011	0.030	7.21E-01	-0.054	0.047	2.46E-01
rs11738223	5	171934492	A	G	0.674	-0.120	0.020	2.00E-08	0.022	0.018	2.19E-01	0.025	0.023	2.84E-01	0.018	0.037	6.23E-01
rs4879656	9	33012382	A	C	0.366	-0.120	0.020	2.00E-08	0.010	0.018	5.71E-01	0.006	0.023	7.78E-01	-0.003	0.035	9.23E-01
rs12599106	16	34498025	A	T	0.496	-0.120	0.020	3.00E-08	-0.018	0.025	4.74E-01	0.006	0.031	8.35E-01	0.006	0.048	8.98E-01
rs9039	16	9205363	C	T	0.279	-0.120	0.020	3.00E-08	-0.004	0.019	8.47E-01	-0.006	0.032	8.43E-01	-0.044	0.049	3.61E-01
rs12196873	6	111598058	A	C	0.848	-0.160	0.031	3.00E-08	-0.005	0.024	8.24E-01	-0.018	0.024	4.55E-01	0.029	0.039	4.56E-01
rs10905065	10	5769827	A	G	0.606	-0.110	0.020	4.00E-08	0.012	0.017	4.88E-01	0.018	0.022	4.22E-01	0.011	0.035	7.51E-01
rs7259376	19	22507705	G	A	0.539	0.110	0.020	4.00E-08	-0.012	0.017	4.85E-01	0.014	0.024	5.61E-01	-0.039	0.038	3.02E-01
rs551087	12	121209193	A	G	0.715	0.130	0.020	4.00E-08	-0.018	0.019	3.35E-01	-0.016	0.022	4.81E-01	-0.022	0.035	5.37E-01

* Total number of SNPs after harmonization and filtering from originally 51 SNPs

SNP: Single Nucleotide Polymorphism
Chr: Chromosome
Pos: Position
EAF: Effect Allele Frequency
SE: Standard Error

Table S7. Physical activity

						Physical activity GWAS			Breast cancer-specific: All			Breast cancer-specific: ER-positive			Breast cancer-specific: ER-negative		
**SNP***	**Chr**	**Pos**	**Effect allele**	**Other allele**	**EAF**	**Beta**	**SE**	**P-value**	**Beta**	**SE**	**P-value**	**Beta**	**SE**	**P-value**	**Beta**	**SE**	**P-value**
rs55657917	17	43844560	T	G	0.787	-0.037	0.005	8.00E-12	-0.015	0.021	4.79E-01	-0.044	0.027	0.1028055	0.069	0.042	1.03E-01
rs59499656	18	40768309	A	T	0.655	-0.028	0.005	2.00E-09	-0.009	0.018	6.19E-01	-0.024	0.023	0.3041047	0.027	0.037	4.60E-01
rs6775319	3	18758501	A	T	0.276	0.027	0.005	4.00E-08	-0.021	0.019	2.74E-01	-0.025	0.025	0.299366	0.006	0.039	4.60E-01

* Total number of SNPs after harmonization and filtering from originally 7 SNPs

SNP: Single Nucleotide Polymorphism
Chr: Chromosome
Pos: Position
EAF: Effect Allele Frequency
SE: Standard Error

Table S8. Smoking behaviour

						Smoking GWAS			Breast cancer-specific: All			Breast cancer-specific: ER-positive			Breast cancer-specific: ER-negative		
SNP*	Chr	Pos	Effect allele	Other allele	EAF	Beta	SE	P-value	Beta	SE	P-value	Beta	SE	P-value	Beta	SE	P-value
rs240955	6	111660643	A	G	0.815	-0.026	0.003	4.00E-23	0.008	0.022	7.02E-01	0.023	0.028	4.19E-01	-0.021	0.044	6.33E-01
rs11783093	8	27425349	T	C	0.156	-0.026	0.003	7.00E-22	-0.002	0.024	9.49E-01	0.028	0.031	3.57E-01	-0.029	0.048	5.51E-01
rs3911063	3	85906928	T	C	0.684	0.019	0.002	5.00E-19	-0.025	0.018	1.60E-01	-0.015	0.023	5.05E-01	-0.066	0.037	7.33E-02
rs6265	11	27679916	T	C	0.198	-0.022	0.003	4.00E-18	-0.003	0.021	9.00E-01	-0.023	0.028	4.12E-01	0.051	0.042	2.24E-01
rs4664442	2	162828001	A	G	0.424	0.016	0.002	2.00E-15	0.016	0.017	3.65E-01	0.039	0.022	7.68E-02	-0.013	0.035	7.05E-01
rs17417989	11	112713857	T	G	0.295	0.018	0.002	1.00E-13	-0.001	0.026	9.68E-01	-0.043	0.034	1.97E-01	0.022	0.050	6.56E-01
rs2310752	1	66392405	A	G	0.426	-0.015	0.002	2.00E-13	0.020	0.018	2.64E-01	0.040	0.023	8.71E-02	0.021	0.037	5.78E-01
rs13258512	8	92777433	A	G	0.577	0.015	0.002	3.00E-13	-0.012	0.018	5.15E-01	-0.040	0.023	8.46E-02	0.031	0.036	3.92E-01
rs10914684	1	33795572	A	G	0.333	-0.015	0.002	1.00E-12	0.004	0.018	8.26E-01	0.023	0.024	3.35E-01	-0.027	0.037	4.67E-01
rs74697736	2	145412271	A	G	0.280	0.017	0.002	3.00E-12	-0.010	0.020	6.16E-01	0.009	0.026	7.18E-01	-0.030	0.041	4.64E-01
rs12764388	10	104413385	A	G	0.116	0.022	0.003	6.00E-12	0.042	0.026	1.05E-01	0.008	0.034	8.08E-01	0.084	0.052	1.09E-01
rs1469908	16	69764412	T	C	0.595	-0.014	0.002	6.00E-12	-0.004	0.017	8.32E-01	0.004	0.022	8.72E-01	-0.023	0.035	5.08E-01
rs4650277	1	74993721	A	G	0.426	0.015	0.002	6.00E-12	0.007	0.017	7.06E-01	0.002	0.022	9.39E-01	0.028	0.034	4.25E-01
rs6852117	4	173076888	C	G	0.570	0.014	0.002	6.00E-12	0.000	0.017	9.98E-01	-0.004	0.022	8.63E-01	0.006	0.035	8.63E-01
rs10994943	10	63591413	T	G	0.598	0.015	0.002	1.00E-11	0.026	0.017	1.38E-01	0.027	0.022	2.22E-01	0.051	0.035	1.48E-01
rs9423279	10	125680419	C	G	0.348	0.015	0.002	1.00E-11	0.025	0.026	3.35E-01	0.014	0.033	6.73E-01	0.016	0.050	7.50E-01
rs17151637	8	10153082	T	C	0.287	-0.015	0.002	2.00E-11	0.028	0.019	1.31E-01	0.029	0.024	2.33E-01	0.007	0.038	8.48E-01
rs2340403	1	73835777	T	C	0.616	-0.014	0.002	2.00E-11	-0.027	0.017	1.19E-01	-0.033	0.022	1.39E-01	-0.021	0.035	5.48E-01
rs597808	12	111973358	A	G	0.488	0.015	0.002	2.00E-11	-0.011	0.017	5.30E-01	-0.025	0.022	2.66E-01	-0.048	0.035	1.66E-01
rs35761479	1	154154194	A	G	0.110	-0.022	0.003	3.00E-11	0.005	0.027	8.46E-01	0.052	0.034	1.25E-01	-0.062	0.056	2.65E-01
rs42417	5	94198290	T	C	0.695	0.016	0.002	3.00E-11	0.008	0.019	6.69E-01	-0.006	0.024	8.18E-01	0.066	0.039	8.92E-02
rs77878475	16	18058548	A	T	0.073	-0.026	0.004	3.00E-11	0.028	0.048	5.56E-01	0.033	0.063	6.02E-01	0.020	0.093	8.26E-01
rs4737525	8	59799781	A	G	0.531	-0.013	0.002	4.00E-11	-0.001	0.023	9.70E-01	-0.011	0.030	7.02E-01	-0.023	0.045	6.10E-01
rs12042107	1	91196176	T	C	0.468	0.014	0.002	5.00E-11	-0.001	0.023	9.55E-01	0.004	0.029	9.00E-01	0.029	0.045	5.23E-01
rs2202237	17	50208784	T	C	0.546	-0.013	0.002	6.00E-11	0.028	0.017	9.90E-02	0.048	0.022	2.93E-02	0.030	0.035	3.86E-01
rs12022778	1	50603995	A	C	0.808	-0.016	0.003	1.00E-10	0.033	0.022	1.23E-01	0.064	0.028	2.30E-02	-0.049	0.043	2.58E-01
rs26251	5	106837450	T	G	0.643	0.013	0.002	1.00E-10	0.004	0.018	8.41E-01	0.004	0.023	8.48E-01	0.014	0.036	6.92E-01
rs1430065	2	103857074	T	C	0.257	0.014	0.002	2.00E-10	-0.064	0.020	1.13E-03	-0.067	0.025	7.69E-03	-0.057	0.040	1.46E-01
rs17733784	18	53719925	T	C	0.626	0.013	0.002	2.00E-10	0.025	0.017	1.58E-01	0.035	0.023	1.16E-01	0.005	0.036	8.89E-01
rs11134672	5	170395820	A	G	0.683	0.014	0.002	3.00E-10	-0.003	0.018	8.90E-01	-0.003	0.024	8.94E-01	0.002	0.037	9.59E-01
rs1373129	9	102153698	C	A	0.509	0.013	0.002	3.00E-10	-0.001	0.017	9.37E-01	0.002	0.023	9.29E-01	-0.028	0.035	4.34E-01
rs2240294	7	96624257	A	T	0.461	-0.013	0.002	3.00E-10	-0.006	0.026	8.15E-01	-0.032	0.033	3.35E-01	0.042	0.050	4.07E-01
rs3818987	6	37484606	T	C	0.482	-0.014	0.002	3.00E-10	0.000	0.018	9.79E-01	0.024	0.023	2.93E-01	-0.067	0.036	6.15E-02
rs4790870	17	1970201	A	C	0.602	0.013	0.002	3.00E-10	-0.040	0.023	8.75E-02	-0.039	0.030	1.93E-01	0.002	0.045	9.65E-01
rs72678864	4	112422145	A	G	0.162	-0.018	0.003	3.00E-10	-0.010	0.025	6.74E-01	0.028	0.032	3.76E-01	-0.055	0.051	2.86E-01
rs883403	7	99047978	C	T	0.153	-0.017	0.003	3.00E-10	-0.010	0.023	6.63E-01	0.026	0.030	3.86E-01	-0.093	0.048	5.47E-02
rs28647734	9	137977033	A	G	0.208	0.017	0.003	4.00E-10	-0.014	0.022	5.35E-01	-0.023	0.029	4.17E-01	0.017	0.044	7.02E-01
rs4949465	1	32178489	T	C	0.860	-0.018	0.003	4.00E-10	0.005	0.033	8.75E-01	-0.008	0.042	8.59E-01	-0.060	0.065	3.56E-01
rs17049905	2	59301572	A	G	0.894	-0.020	0.003	5.00E-10	-0.014	0.029	6.22E-01	0.008	0.038	8.37E-01	-0.008	0.059	8.95E-01
rs10952199	7	1665339	T	C	0.415	-0.014	0.002	6.00E-10	0.035	0.023	1.27E-01	0.042	0.030	1.61E-01	-0.044	0.046	3.39E-01
rs13187930	5	60384171	T	C	0.256	0.014	0.002	6.00E-10	-0.040	0.020	4.30E-02	-0.027	0.025	2.85E-01	-0.046	0.040	2.46E-01
rs4814884	20	19663293	T	C	0.512	0.012	0.002	6.00E-10	0.001	0.017	9.39E-01	0.011	0.022	6.24E-01	-0.038	0.034	2.65E-01
rs479971	12	69672471	A	G	0.286	0.015	0.003	7.00E-10	0.025	0.025	3.24E-01	0.068	0.032	3.59E-02	-0.022	0.049	6.53E-01
rs4856463	3	83638568	T	C	0.230	-0.016	0.003	7.00E-10	-0.022	0.021	2.97E-01	-0.033	0.027	2.12E-01	-0.036	0.042	3.94E-01
rs1109480	12	121083279	A	G	0.380	-0.014	0.002	9.00E-10	0.015	0.018	3.92E-01	0.028	0.023	2.14E-01	-0.003	0.036	9.37E-01
rs1891196	1	208718672	G	A	0.444	0.012	0.002	9.00E-10	0.015	0.018	4.02E-01	0.009	0.023	6.95E-01	0.019	0.036	6.08E-01
rs12211126	6	67549451	T	C	0.610	-0.012	0.002	1.00E-09	-0.008	0.018	6.41E-01	-0.019	0.023	4.06E-01	0.019	0.037	6.12E-01
rs12690535	2	226259373	A	G	0.831	0.016	0.003	1.00E-09	-0.008	0.031	8.02E-01	0.002	0.039	9.54E-01	-0.017	0.060	7.74E-01
rs13065007	3	117522712	A	T	0.272	0.014	0.002	1.00E-09	0.009	0.019	6.28E-01	-0.015	0.025	5.33E-01	0.086	0.039	2.62E-02
rs10780649	9	86703561	T	G	0.489	-0.013	0.002	2.00E-09	0.004	0.017	8.13E-01	-0.006	0.022	7.81E-01	0.027	0.035	4.37E-01
rs10970595	9	31910764	T	C	0.105	-0.019	0.003	2.00E-09	-0.024	0.029	4.13E-01	-0.048	0.038	2.05E-01	0.017	0.059	7.75E-01
rs11872397	18	72535282	A	G	0.267	-0.015	0.003	2.00E-09	-0.002	0.027	9.29E-01	-0.061	0.035	7.95E-02	0.043	0.051	4.00E-01
rs12535004	7	114947112	T	C	0.740	-0.015	0.003	2.00E-09	0.000	0.026	9.97E-01	-0.009	0.033	7.90E-01	-0.004	0.049	9.36E-01
rs12787182	11	59190811	A	G	0.715	0.013	0.002	2.00E-09	0.003	0.025	9.14E-01	0.003	0.032	9.19E-01	-0.046	0.049	3.51E-01
rs13357015	5	80263403	A	G	0.637	0.014	0.002	2.00E-09	-0.002	0.018	8.99E-01	0.023	0.023	3.04E-01	-0.026	0.036	4.64E-01
rs17373738	4	140935264	C	G	0.623	0.012	0.002	2.00E-09	-0.025	0.018	1.61E-01	-0.061	0.023	7.94E-03	0.017	0.036	6.40E-01
rs1775370	1	72992856	T	C	0.248	-0.014	0.002	2.00E-09	-0.005	0.020	8.07E-01	-0.033	0.026	1.97E-01	0.058	0.040	1.48E-01
rs1863161	2	60139524	A	G	0.583	0.012	0.002	2.00E-09	0.001	0.017	9.69E-01	0.010	0.022	6.56E-01	-0.035	0.035	3.21E-01
rs1486900	15	47642886	T	G	0.172	0.016	0.003	3.00E-09	0.002	0.023	9.19E-01	0.006	0.029	8.38E-01	-0.024	0.045	5.95E-01
rs16951001	15	67854241	T	G	0.416	0.012	0.002	3.00E-09	0.026	0.017	1.29E-01	0.017	0.022	4.34E-01	0.024	0.035	4.87E-01
rs176644	15	89913632	T	G	0.420	-0.012	0.002	3.00E-09	-0.031	0.017	7.85E-02	-0.013	0.022	5.65E-01	-0.087	0.036	1.40E-02
rs2291256	12	133393323	T	C	0.085	0.021	0.004	3.00E-09	-0.018	0.042	6.71E-01	0.047	0.054	3.78E-01	-0.100	0.084	2.35E-01
rs2866724	16	13760152	A	G	0.722	-0.014	0.002	3.00E-09	0.001	0.020	9.72E-01	0.008	0.025	7.64E-01	0.018	0.040	6.45E-01
rs2939756	11	41436297	A	G	0.475	-0.012	0.002	3.00E-09	-0.006	0.018	7.16E-01	-0.007	0.023	7.64E-01	-0.003	0.036	9.36E-01
rs11166986	8	141656155	A	G	0.491	0.012	0.002	4.00E-09	-0.007	0.017	6.88E-01	-0.029	0.022	1.93E-01	-0.043	0.035	2.20E-01
rs17348216	5	103924374	T	C	0.176	-0.016	0.003	4.00E-09	0.037	0.022	9.50E-02	0.023	0.029	4.16E-01	0.055	0.045	2.25E-01
rs4680392	3	157404329	C	T	0.692	0.014	0.002	4.00E-09	-0.001	0.019	9.49E-01	-0.004	0.024	8.83E-01	0.013	0.039	7.40E-01
rs6598539	15	99204483	C	T	0.516	0.013	0.002	4.00E-09	-0.019	0.018	2.89E-01	-0.049	0.023	3.16E-02	0.005	0.035	8.88E-01
rs7160389	14	98650988	T	C	0.533	-0.012	0.002	4.00E-09	0.019	0.017	2.85E-01	-0.001	0.022	9.54E-01	0.031	0.035	3.76E-01
rs772921	12	56403577	T	C	0.322	-0.012	0.002	4.00E-09	-0.035	0.018	5.04E-02	-0.025	0.023	2.82E-01	-0.024	0.037	5.14E-01
rs11771982	7	132302248	T	C	0.144	-0.016	0.003	5.00E-09	-0.038	0.025	1.21E-01	-0.043	0.032	1.80E-01	-0.019	0.051	7.08E-01
rs3800227	6	108994161	A	G	0.265	-0.013	0.002	5.00E-09	-0.005	0.019	7.87E-01	0.020	0.025	4.12E-01	-0.043	0.039	2.78E-01
rs11808210	1	227515049	A	G	0.075	0.022	0.004	6.00E-09	0.032	0.032	3.19E-01	0.016	0.042	7.05E-01	0.102	0.064	1.14E-01
rs11876432	18	42669425	C	G	0.756	0.013	0.002	7.00E-09	0.032	0.021	1.17E-01	0.026	0.027	3.37E-01	0.058	0.042	1.68E-01
rs12930834	16	72951627	A	C	0.772	0.014	0.002	7.00E-09	-0.009	0.020	6.52E-01	-0.020	0.026	4.30E-01	-0.001	0.041	9.90E-01
rs3851186	11	85931325	C	T	0.781	0.015	0.003	7.00E-09	-0.001	0.020	9.79E-01	-0.012	0.026	6.60E-01	0.031	0.041	4.56E-01
rs4837631	9	122061948	T	C	0.443	-0.013	0.002	7.00E-09	-0.005	0.023	8.44E-01	0.000	0.030	9.96E-01	-0.005	0.044	9.08E-01
rs2236941	3	50247824	T	C	0.333	0.012	0.002	8.00E-09	0.026	0.018	1.46E-01	0.024	0.023	3.13E-01	0.054	0.036	1.35E-01
rs55921136	1	210359333	T	C	0.788	0.016	0.003	8.00E-09	0.018	0.022	4.06E-01	0.042	0.028	1.34E-01	0.015	0.044	7.43E-01
rs78175438	21	40663255	T	C	0.883	-0.019	0.003	8.00E-09	-0.008	0.028	7.68E-01	0.000	0.036	9.95E-01	-0.018	0.055	7.45E-01
rs962472	10	56700597	T	C	0.489	-0.012	0.002	8.00E-09	0.000	0.017	9.93E-01	-0.007	0.022	7.38E-01	0.033	0.035	3.48E-01
rs10160101	10	13549462	C	G	0.385	-0.012	0.002	1.00E-08	0.024	0.018	1.73E-01	0.023	0.023	3.13E-01	0.009	0.036	7.97E-01
rs1174864	7	53127559	A	G	0.539	0.012	0.002	1.00E-08	0.010	0.017	5.47E-01	0.003	0.022	8.80E-01	0.042	0.035	2.34E-01
rs12662631	6	93842446	T	C	0.318	0.012	0.002	1.00E-08	0.016	0.019	4.05E-01	0.024	0.024	3.13E-01	-0.035	0.038	3.57E-01
rs2185913	10	115410234	T	C	0.705	0.012	0.002	1.00E-08	0.004	0.019	8.45E-01	0.016	0.024	5.19E-01	-0.016	0.038	6.61E-01
rs2222016	2	137143307	C	G	0.679	0.013	0.002	1.00E-08	0.026	0.020	1.79E-01	0.034	0.025	1.81E-01	0.049	0.040	2.15E-01
rs2783130	13	80170160	A	G	0.514	0.011	0.002	1.00E-08	0.002	0.017	8.90E-01	0.001	0.022	9.81E-01	0.030	0.035	3.92E-01
rs4479577	3	5723818	T	C	0.479	0.012	0.002	1.00E-08	0.013	0.017	4.35E-01	0.005	0.022	8.33E-01	0.024	0.034	4.86E-01
rs6061162	20	29893501	T	C	0.913	0.020	0.004	1.00E-08	-0.030	0.030	3.13E-01	-0.083	0.038	2.94E-02	0.100	0.061	9.99E-02
rs7130826	11	17037793	T	G	0.733	0.013	0.002	1.00E-08	-0.002	0.026	9.47E-01	0.007	0.033	8.34E-01	-0.055	0.050	2.77E-01
rs745570	17	77781725	A	G	0.503	0.011	0.002	1.00E-08	0.024	0.017	1.56E-01	0.008	0.022	7.07E-01	0.028	0.035	4.21E-01
rs9395644	6	50923637	G	A	0.103	-0.020	0.004	1.00E-08	-0.026	0.028	3.45E-01	-0.022	0.035	5.29E-01	-0.013	0.056	8.17E-01
rs10020574	4	28062966	C	G	0.246	0.013	0.002	2.00E-08	0.007	0.021	7.51E-01	0.015	0.027	5.87E-01	-0.021	0.042	6.21E-01
rs10461104	4	27490579	A	G	0.632	0.013	0.002	2.00E-08	0.034	0.018	4.95E-02	0.034	0.023	1.31E-01	0.007	0.036	8.38E-01
rs117898875	3	84272987	T	C	0.966	-0.032	0.006	2.00E-08	-0.013	0.048	7.83E-01	-0.026	0.062	6.77E-01	0.038	0.097	6.92E-01
rs13160204	5	157734762	A	G	0.299	0.012	0.002	2.00E-08	0.030	0.018	9.99E-02	0.035	0.024	1.35E-01	0.039	0.037	2.94E-01
rs1449435	11	132088917	A	G	0.419	0.012	0.002	2.00E-08	-0.022	0.018	2.23E-01	-0.030	0.023	1.89E-01	0.021	0.036	5.62E-01
rs56081685	13	59454140	T	G	0.702	0.013	0.002	2.00E-08	-0.027	0.019	1.57E-01	-0.038	0.025	1.21E-01	0.006	0.039	8.82E-01
rs969650	9	109378080	T	C	0.639	0.012	0.002	2.00E-08	0.006	0.018	7.34E-01	-0.003	0.023	8.85E-01	0.057	0.036	1.14E-01
rs10751226	11	73312252	T	C	0.826	-0.015	0.003	3.00E-08	-0.017	0.029	5.63E-01	-0.037	0.038	3.34E-01	0.002	0.058	9.71E-01
rs1150668	6	28129789	T	G	0.579	0.011	0.002	3.00E-08	0.000	0.017	9.84E-01	-0.024	0.022	2.83E-01	0.016	0.035	6.57E-01
rs1271272	2	207955477	A	G	0.316	-0.012	0.002	3.00E-08	0.007	0.018	7.01E-01	0.023	0.024	3.29E-01	0.024	0.037	5.12E-01
rs2652434	2	155742894	T	C	0.466	-0.011	0.002	3.00E-08	-0.014	0.017	4.11E-01	-0.024	0.022	2.66E-01	0.023	0.035	5.08E-01
rs3026629	22	38329285	A	G	0.350	-0.012	0.002	3.00E-08	0.007	0.018	6.86E-01	0.011	0.023	6.25E-01	0.017	0.036	6.31E-01
rs6971782	7	70526891	T	C	0.313	-0.012	0.002	3.00E-08	-0.007	0.020	7.38E-01	-0.019	0.025	4.63E-01	-0.008	0.040	8.49E-01
rs7645872	3	25209801	T	C	0.480	0.012	0.002	3.00E-08	0.016	0.018	3.86E-01	0.014	0.023	5.55E-01	0.043	0.036	2.33E-01
rs12476173	2	118247714	A	G	0.615	0.012	0.002	4.00E-08	0.012	0.018	4.90E-01	0.014	0.023	5.59E-01	-0.030	0.036	4.03E-01
rs12517438	5	30842054	T	G	0.469	-0.011	0.002	4.00E-08	-0.012	0.018	5.03E-01	-0.008	0.023	7.41E-01	-0.015	0.036	6.83E-01
rs12997449	2	179039572	A	G	0.877	-0.018	0.003	4.00E-08	0.007	0.026	7.86E-01	0.085	0.035	1.40E-02	-0.139	0.052	7.80E-03
rs146127221	10	87465134	T	C	0.021	0.042	0.008	4.00E-08	-0.067	0.061	2.73E-01	-0.185	0.082	2.31E-02	0.129	0.116	2.66E-01
rs28459916	4	80330743	A	C	0.142	-0.017	0.003	4.00E-08	0.010	0.025	6.91E-01	0.013	0.033	6.84E-01	0.050	0.051	3.25E-01
rs4312833	5	50982154	A	T	0.094	-0.019	0.004	4.00E-08	-0.020	0.029	4.88E-01	-0.037	0.038	3.38E-01	0.041	0.058	4.81E-01
rs58695218	7	39432330	A	G	0.811	0.015	0.003	4.00E-08	-0.023	0.022	2.91E-01	-0.029	0.028	3.07E-01	-0.036	0.044	4.15E-01
rs6438208	3	114170272	A	G	0.254	-0.013	0.002	4.00E-08	0.006	0.027	8.36E-01	-0.010	0.035	7.75E-01	0.044	0.052	3.94E-01
rs7295765	12	86459040	A	C	0.807	-0.015	0.003	4.00E-08	-0.022	0.022	3.07E-01	-0.024	0.028	3.82E-01	0.010	0.044	8.13E-01
rs7901883	10	103186838	A	G	0.225	-0.013	0.002	4.00E-08	0.013	0.020	5.04E-01	0.020	0.026	4.39E-01	0.010	0.041	8.00E-01
rs9388686	6	129425564	C	A	0.829	0.014	0.003	4.00E-08	-0.026	0.022	2.46E-01	-0.017	0.029	5.52E-01	-0.053	0.044	2.34E-01
rs12895462	14	77615441	T	C	0.815	0.014	0.003	5.00E-08	0.007	0.023	7.53E-01	-0.021	0.030	4.76E-01	0.054	0.047	2.49E-01
rs7575189	2	174015168	A	G	0.592	-0.011	0.002	5.00E-08	0.007	0.017	6.84E-01	-0.009	0.022	6.70E-01	0.026	0.035	4.49E-01

* Total number of SNPs after harmonization and filtering from originally 223 SNPs

SNP: Single Nucleotide Polymorphism
Chr: Chromosome
Pos: Position
EAF: Effect Allele Frequency
SE: Standard Error

Table S9. Type 2 diabetes mellitus

						T2DM GWAS			Breast cancer-specific: All			Breast cancer-specific: ER-positive			Breast cancer-specific: ER-negative		
**SNP***	**Chr**	**Pos**	**Effect allele**	**Other allele**	**EAF**	**Beta**	**SE**	**P-value**	**Beta**	**SE**	**P-value**	**Beta**	**SE**	**P-value**	**Beta**	**SE**	**P-value**
rs7903146	10	114758349	C	T	0.289	-0.306	0.008	1.00E-200	0.012	0.018	5.21E-01	-0.009	0.024	7.06E-01	0.002	0.037	9.56E-01
rs7756992	6	20679709	A	G	0.280	-0.130	0.008	6.00E-62	0.021	0.019	2.58E-01	0.016	0.024	5.09E-01	0.031	0.038	4.16E-01
rs7651090	3	185513392	G	A	0.691	0.120	0.008	4.00E-57	0.005	0.018	8.03E-01	0.024	0.024	3.17E-01	-0.037	0.037	3.22E-01
rs3802177	8	118185025	A	G	0.697	-0.122	0.008	2.00E-52	-0.008	0.018	6.59E-01	-0.019	0.024	4.32E-01	0.034	0.037	3.52E-01
rs7185735	16	53822651	A	G	0.395	-0.106	0.007	2.00E-47	-0.010	0.017	5.77E-01	-0.007	0.022	7.40E-01	-0.007	0.035	8.37E-01
rs849135	7	28196413	G	A	0.490	0.100	0.007	1.00E-43	0.022	0.017	1.88E-01	0.008	0.022	7.10E-01	0.042	0.034	2.21E-01
rs7923866	10	94482076	T	C	0.629	-0.097	0.007	9.00E-40	-0.001	0.018	9.51E-01	0.017	0.023	4.59E-01	0.016	0.036	6.65E-01
rs2972144	2	227101411	A	G	0.628	-0.091	0.007	3.00E-34	0.008	0.018	6.71E-01	-0.001	0.023	9.59E-01	0.038	0.036	2.83E-01
rs1801214	4	6303022	C	T	0.594	-0.090	0.007	6.00E-34	-0.002	0.017	9.10E-01	-0.007	0.022	7.47E-01	-0.039	0.035	2.71E-01
rs10830963	11	92708710	G	C	0.721	0.091	0.008	6.00E-30	0.003	0.020	8.77E-01	0.007	0.026	7.78E-01	-0.004	0.040	9.13E-01
rs11708067	3	123065778	G	A	0.771	-0.097	0.008	6.00E-29	-0.031	0.021	1.27E-01	-0.030	0.027	2.60E-01	-0.027	0.041	5.16E-01
rs17334919	2	43707385	T	C	0.908	-0.140	0.013	7.00E-28	-0.019	0.029	5.06E-01	-0.011	0.037	7.57E-01	-0.040	0.059	5.00E-01
rs1552224	11	72433098	C	A	0.841	-0.103	0.010	9.00E-25	-0.001	0.024	9.78E-01	0.002	0.030	9.48E-01	0.034	0.047	4.64E-01
rs1899951	3	12394840	T	C	0.872	-0.112	0.011	2.00E-24	0.017	0.026	5.03E-01	-0.030	0.034	3.75E-01	0.068	0.052	1.89E-01
rs1359790	13	80717156	A	G	0.722	-0.080	0.008	3.00E-23	-0.032	0.019	9.54E-02	-0.035	0.025	1.58E-01	-0.004	0.039	9.15E-01
rs13389219	2	165528876	T	C	0.591	-0.072	0.007	2.00E-22	-0.001	0.017	9.40E-01	-0.010	0.022	6.43E-01	-0.008	0.035	8.17E-01
rs2796441	9	84308948	A	G	0.593	-0.072	0.007	2.00E-22	0.014	0.023	5.56E-01	0.031	0.030	2.95E-01	0.005	0.045	9.17E-01
rs516946	8	41519248	T	C	0.755	-0.082	0.008	3.00E-22	-0.004	0.020	8.45E-01	-0.011	0.025	6.62E-01	0.008	0.041	8.37E-01
rs11651755	17	36099840	C	T	0.515	0.074	0.008	9.00E-22	0.018	0.017	2.94E-01	0.014	0.022	5.20E-01	0.024	0.035	4.82E-01
rs780094	2	27741237	T	C	0.626	-0.069	0.007	5.00E-21	0.014	0.017	4.15E-01	0.013	0.022	5.46E-01	0.038	0.035	2.87E-01
rs5215	11	17408630	C	T	0.636	0.068	0.007	2.00E-20	0.026	0.017	1.36E-01	0.037	0.022	9.57E-02	-0.019	0.035	5.82E-01
rs2191348	7	15064255	G	T	0.548	-0.065	0.007	3.00E-19	-0.001	0.017	9.75E-01	0.014	0.022	5.22E-01	0.019	0.034	5.76E-01
rs1063192	9	22003367	G	A	0.556	-0.063	0.007	3.00E-18	0.000	0.017	9.84E-01	0.024	0.022	2.81E-01	-0.004	0.035	9.04E-01
rs1496653	3	23454790	G	A	0.801	-0.077	0.009	3.00E-18	0.015	0.028	5.95E-01	0.041	0.036	2.57E-01	-0.011	0.054	8.35E-01
rs340874	1	214159256	T	C	0.545	-0.063	0.007	8.00E-18	-0.027	0.018	1.32E-01	-0.017	0.023	4.50E-01	-0.019	0.036	6.03E-01
rs459193	5	55806751	A	G	0.744	-0.071	0.008	9.00E-18	-0.004	0.020	8.53E-01	-0.009	0.026	7.35E-01	-0.049	0.041	2.35E-01
rs7729395	5	102100576	T	C	0.951	0.137	0.015	1.00E-17	0.093	0.059	1.14E-01	0.078	0.076	3.05E-01	0.079	0.113	4.84E-01
rs2261181	12	66212318	T	C	0.903	0.099	0.012	9.00E-17	0.036	0.028	2.04E-01	0.068	0.036	5.79E-02	-0.114	0.059	5.29E-02
rs7177055	15	77832762	G	A	0.712	-0.065	0.008	3.00E-16	0.012	0.019	5.36E-01	-0.012	0.025	6.33E-01	0.030	0.038	4.27E-01
rs8108269	19	46158513	G	T	0.702	0.064	0.008	3.00E-16	-0.046	0.019	1.42E-02	-0.018	0.024	4.45E-01	-0.077	0.038	4.27E-02
rs17168486	7	14898282	T	C	0.827	0.074	0.009	2.00E-15	0.017	0.023	4.56E-01	0.009	0.030	7.66E-01	0.042	0.046	3.57E-01
rs243019	2	60585806	C	T	0.535	0.057	0.007	2.00E-15	-0.015	0.017	3.92E-01	-0.015	0.022	5.03E-01	-0.002	0.034	9.49E-01
rs10974438	9	4291928	C	A	0.636	0.059	0.008	3.00E-15	0.040	0.019	3.21E-02	0.049	0.024	4.21E-02	0.015	0.037	6.88E-01
rs61953351	12	121456616	T	G	0.752	-0.070	0.009	2.00E-14	0.000	0.019	9.99E-01	0.004	0.025	8.72E-01	-0.023	0.039	5.51E-01
rs6795735	3	64705365	C	T	0.414	0.056	0.007	2.00E-14	-0.018	0.017	3.03E-01	-0.008	0.022	7.14E-01	-0.016	0.035	6.54E-01
rs8068804	17	3985864	A	G	0.679	0.059	0.008	4.00E-14	0.004	0.018	8.16E-01	0.004	0.024	8.81E-01	0.011	0.037	7.66E-01
rs2857605	6	31524851	C	T	0.796	-0.067	0.009	6.00E-14	-0.022	0.021	2.95E-01	-0.011	0.027	6.90E-01	-0.036	0.043	4.08E-01
rs13234269	7	130429186	A	T	0.519	-0.058	0.008	7.00E-14	-0.063	0.023	5.86E-03	-0.080	0.029	6.35E-03	-0.040	0.045	3.78E-01
rs9894220	17	46989154	G	A	0.578	-0.059	0.008	2.00E-13	0.004	0.018	8.20E-01	0.004	0.023	8.49E-01	-0.011	0.036	7.63E-01
rs825476	12	124568456	C	T	0.578	-0.052	0.007	7.00E-13	0.014	0.017	4.16E-01	0.021	0.022	3.49E-01	-0.008	0.035	8.14E-01
rs2820426	1	219660535	A	G	0.631	-0.052	0.007	1.00E-12	-0.013	0.018	4.69E-01	-0.024	0.023	3.00E-01	-0.028	0.036	4.30E-01
rs17791483	9	81898980	G	A	0.937	-0.102	0.015	3.00E-12	0.041	0.034	2.23E-01	0.036	0.044	4.04E-01	0.056	0.069	4.15E-01
rs10401969	19	19407718	C	T	0.921	0.092	0.013	4.00E-12	0.007	0.031	8.36E-01	-0.026	0.041	5.21E-01	0.011	0.063	8.67E-01
rs12970134	18	57884750	A	G	0.734	0.056	0.008	5.00E-12	0.040	0.019	3.81E-02	0.026	0.025	2.88E-01	0.091	0.039	1.90E-02
rs853974	6	127068983	T	C	0.740	0.060	0.009	8.00E-12	0.016	0.020	4.08E-01	0.020	0.025	4.26E-01	0.072	0.040	7.21E-02
rs2925979	16	81534790	T	C	0.702	0.053	0.008	9.00E-12	-0.011	0.019	5.45E-01	0.014	0.024	5.60E-01	-0.019	0.038	6.21E-01
rs12088739	1	51506886	G	A	0.910	-0.088	0.013	1.00E-11	0.075	0.029	9.52E-03	0.064	0.037	8.29E-02	0.093	0.059	1.12E-01
rs4865796	5	53272664	G	A	0.695	-0.053	0.008	1.00E-11	0.005	0.018	7.89E-01	-0.002	0.024	9.20E-01	-0.011	0.037	7.60E-01
rs7572970	2	161136656	A	G	0.743	-0.059	0.009	1.00E-11	-0.037	0.019	4.86E-02	-0.031	0.024	2.06E-01	-0.055	0.039	1.59E-01
rs576674	13	33554302	G	A	0.832	0.065	0.010	2.00E-11	0.030	0.023	1.90E-01	0.019	0.030	5.29E-01	-0.004	0.047	9.25E-01
rs735949	4	185716232	C	T	0.856	-0.071	0.011	2.00E-11	-0.029	0.026	2.55E-01	-0.013	0.033	6.92E-01	-0.056	0.053	2.90E-01
rs1050226	6	7281654	G	A	0.597	-0.049	0.007	3.00E-11	-0.009	0.023	7.12E-01	-0.031	0.030	2.93E-01	0.040	0.045	3.67E-01
rs4932143	15	90372067	G	C	0.726	0.057	0.009	6.00E-11	0.023	0.025	3.60E-01	0.043	0.033	1.89E-01	0.028	0.049	5.73E-01
rs72892910	6	50816887	T	G	0.818	0.065	0.010	6.00E-11	0.000	0.022	9.87E-01	0.008	0.029	7.83E-01	0.005	0.045	9.07E-01
rs2071479	6	32781112	T	C	0.967	0.147	0.023	7.00E-11	0.100	0.045	2.44E-02	0.163	0.056	3.58E-03	0.050	0.094	5.94E-01
rs2493394	1	120471224	G	A	0.891	0.073	0.011	1.00E-10	0.026	0.027	3.40E-01	0.018	0.035	6.10E-01	0.073	0.054	1.78E-01
rs9369425	6	43810974	G	A	0.711	0.055	0.008	1.00E-10	-0.005	0.019	7.86E-01	-0.021	0.024	3.77E-01	-0.005	0.038	8.99E-01
rs7685296	4	153254121	T	C	0.732	-0.051	0.008	2.00E-10	0.015	0.020	4.61E-01	-0.022	0.026	3.90E-01	0.029	0.041	4.70E-01
rs13239186	7	117510621	T	C	0.679	0.054	0.009	3.00E-10	0.021	0.018	2.59E-01	0.030	0.024	2.04E-01	0.011	0.038	7.73E-01
rs4823182	22	44377442	G	A	0.641	0.048	0.008	3.00E-10	0.061	0.019	9.26E-04	0.065	0.024	6.34E-03	0.061	0.038	1.03E-01
rs622217	6	160766770	T	C	0.498	0.049	0.008	3.00E-10	0.003	0.017	8.41E-01	-0.003	0.022	8.81E-01	-0.022	0.035	5.25E-01
rs993380	4	83584496	A	G	0.655	0.051	0.008	5.00E-10	0.021	0.018	2.31E-01	-0.002	0.023	9.16E-01	0.049	0.036	1.65E-01
rs7138300	12	71439589	C	T	0.550	0.044	0.007	6.00E-10	0.003	0.018	8.80E-01	0.004	0.023	8.75E-01	0.022	0.036	5.31E-01
rs7674212	4	103988899	G	T	0.420	0.047	0.007	6.00E-10	-0.004	0.017	8.10E-01	0.012	0.022	5.91E-01	-0.055	0.035	1.16E-01
rs1758632	9	34025640	C	G	0.617	-0.049	0.008	1.00E-09	-0.009	0.018	6.22E-01	-0.009	0.023	6.86E-01	0.013	0.036	7.16E-01
rs12945601	17	17653411	T	C	0.598	0.048	0.008	2.00E-09	-0.006	0.017	7.15E-01	0.005	0.022	8.33E-01	-0.006	0.035	8.60E-01
rs17405722	17	40542501	A	G	0.931	0.087	0.015	2.00E-09	0.013	0.033	6.90E-01	0.021	0.043	6.15E-01	0.025	0.067	7.03E-01
rs348330	1	229672955	G	A	0.646	0.049	0.008	2.00E-09	0.011	0.019	5.59E-01	0.023	0.025	3.54E-01	0.038	0.039	3.23E-01
rs3887925	3	186665645	C	T	0.557	-0.047	0.008	2.00E-09	-0.014	0.017	4.22E-01	-0.010	0.022	6.51E-01	-0.016	0.035	6.37E-01
rs6066138	20	45594711	G	A	0.255	0.049	0.008	2.00E-09	-0.031	0.026	2.33E-01	-0.052	0.033	1.18E-01	0.004	0.051	9.40E-01
rs7929543	11	49351026	C	A	0.919	0.083	0.014	2.00E-09	-0.022	0.031	4.83E-01	-0.034	0.041	3.95E-01	-0.013	0.064	8.33E-01
rs10087241	8	30863722	G	A	0.581	0.048	0.008	3.00E-09	0.000	0.017	9.91E-01	-0.023	0.022	3.05E-01	0.025	0.036	4.78E-01
rs11048456	12	26463082	C	T	0.748	0.049	0.008	3.00E-09	-0.022	0.020	2.63E-01	-0.030	0.026	2.36E-01	0.000	0.040	9.91E-01
rs244415	16	69666683	A	G	0.569	-0.047	0.008	4.00E-09	-0.003	0.017	8.46E-01	-0.007	0.022	7.46E-01	0.009	0.035	7.86E-01
rs2908282	7	44248828	G	A	0.167	-0.055	0.009	4.00E-09	0.001	0.030	9.65E-01	-0.002	0.039	9.68E-01	-0.072	0.060	2.28E-01
rs7845219	8	95937502	C	T	0.523	-0.042	0.007	5.00E-09	-0.033	0.017	5.39E-02	-0.025	0.022	2.51E-01	-0.046	0.034	1.80E-01
rs2616132	10	71469514	A	G	0.534	0.046	0.008	7.00E-09	-0.012	0.017	4.72E-01	0.000	0.022	9.87E-01	-0.056	0.034	1.03E-01
rs7931302	11	128236058	C	A	0.721	0.046	0.008	8.00E-09	0.035	0.026	1.75E-01	0.040	0.033	2.27E-01	0.069	0.049	1.60E-01
rs16988333	22	30552813	G	A	0.922	-0.075	0.013	9.00E-09	-0.002	0.031	9.43E-01	0.038	0.040	3.36E-01	-0.133	0.065	4.13E-02
rs10114341	9	96919182	C	T	0.551	-0.041	0.007	1.00E-08	-0.024	0.017	1.56E-01	-0.019	0.022	3.86E-01	-0.023	0.035	5.07E-01
rs2292662	3	63897215	T	C	0.848	-0.063	0.011	1.00E-08	0.006	0.023	7.97E-01	0.010	0.030	7.25E-01	-0.109	0.048	2.28E-02
rs2299383	7	103418846	T	C	0.568	0.041	0.007	1.00E-08	-0.005	0.018	7.66E-01	-0.019	0.023	4.11E-01	0.022	0.036	5.47E-01
rs7786095	7	156983847	G	A	0.898	-0.074	0.013	1.00E-08	-0.009	0.029	7.50E-01	0.011	0.037	7.64E-01	-0.050	0.059	3.94E-01
rs11098676	4	123833154	T	C	0.783	-0.054	0.010	2.00E-08	-0.043	0.020	3.52E-02	-0.028	0.026	2.82E-01	-0.066	0.041	1.12E-01
rs1127655	1	117530507	C	T	0.534	0.044	0.008	2.00E-08	-0.005	0.017	7.57E-01	0.027	0.022	2.26E-01	-0.057	0.035	1.08E-01
rs11925227	3	170766618	A	G	0.818	-0.053	0.009	2.00E-08	-0.004	0.031	9.02E-01	-0.014	0.040	7.24E-01	0.017	0.058	7.71E-01
rs11926707	3	46925539	T	C	0.626	-0.046	0.008	2.00E-08	-0.024	0.018	1.76E-01	-0.036	0.023	1.17E-01	-0.014	0.036	7.08E-01
rs17086692	4	53134293	T	G	0.675	-0.047	0.008	2.00E-08	-0.015	0.024	5.25E-01	0.008	0.031	8.01E-01	-0.082	0.048	8.58E-02
rs2294120	8	146003567	G	A	0.531	-0.044	0.008	2.00E-08	0.010	0.024	6.91E-01	0.040	0.031	1.94E-01	-0.065	0.047	1.60E-01
rs2633310	10	75594050	G	T	0.428	0.044	0.008	2.00E-08	0.003	0.018	8.74E-01	0.014	0.023	5.31E-01	-0.002	0.036	9.65E-01
rs4502156	15	62383155	C	T	0.551	-0.041	0.007	2.00E-08	-0.019	0.018	2.87E-01	-0.015	0.024	5.29E-01	-0.027	0.037	4.66E-01
rs4810426	20	43001721	T	C	0.876	0.073	0.013	2.00E-08	0.009	0.027	7.55E-01	-0.036	0.036	3.07E-01	0.070	0.055	2.04E-01
rs6059662	20	32675727	A	G	0.702	-0.045	0.008	2.00E-08	0.011	0.018	5.54E-01	0.023	0.024	3.45E-01	0.022	0.037	5.64E-01
rs7144011	14	79940383	T	G	0.782	0.048	0.008	2.00E-08	0.005	0.021	8.11E-01	-0.016	0.027	5.65E-01	0.024	0.042	5.71E-01
rs963740	13	51096095	T	A	0.705	-0.048	0.009	2.00E-08	-0.009	0.019	6.36E-01	-0.020	0.024	4.08E-01	0.017	0.038	6.58E-01
rs17411031	8	19852310	G	C	0.736	-0.045	0.008	3.00E-08	-0.011	0.019	5.57E-01	-0.021	0.025	3.88E-01	0.049	0.039	2.07E-01
rs2307111	5	75003678	T	C	0.404	0.041	0.007	3.00E-08	-0.013	0.017	4.42E-01	-0.028	0.022	2.11E-01	0.024	0.035	4.93E-01
rs6515236	20	22435749	C	A	0.745	-0.050	0.009	3.00E-08	0.018	0.026	4.92E-01	0.026	0.033	4.32E-01	-0.005	0.051	9.21E-01
rs7561798	2	228973660	G	A	0.526	0.040	0.007	3.00E-08	0.026	0.023	2.47E-01	0.001	0.029	9.64E-01	0.063	0.044	1.59E-01
rs10169613	2	111934977	T	C	0.528	-0.043	0.008	4.00E-08	0.013	0.017	4.39E-01	-0.015	0.022	4.92E-01	0.018	0.035	6.00E-01
rs11107116	12	93978504	G	T	0.224	-0.047	0.008	4.00E-08	-0.040	0.021	5.06E-02	-0.026	0.027	3.21E-01	-0.032	0.041	4.43E-01
rs17631783	17	61687600	C	T	0.282	0.049	0.009	4.00E-08	0.002	0.020	9.07E-01	-0.016	0.026	5.34E-01	0.037	0.040	3.57E-01
rs10077431	5	112927686	A	C	0.792	-0.049	0.009	5.00E-08	-0.025	0.022	2.51E-01	-0.030	0.028	2.98E-01	0.027	0.044	5.31E-01

* Total number of SNPs after harmonization and filtering from originally 138 SNPs

SNPs removed. Overlapping between BMI and T2DM

SNPs removed. In linkage desequilibrium with SNPs in BMI

SNP: Single Nucleotide Polymorphism
Chr: Chromosome
Pos: Position
EAF: Effect Allele Frequency
SE: Standard Error

Table S10. Body mass index European-specific

						BMI-European GWAS			Breast cancer-specific: All		
**SNP**	**Chr**	**Pos**	**Effect allele**	**Other allele**	**EAF**	**Beta**	**SE**	**P-value**	**Beta**	**SE**	**P-value**
rs10132280	14	25928179	A	C	0.333	-0.023	0.003	1.14E-11	-0.012	0.019	5.23E-01
rs1016287	2	59305625	T	C	0.325	0.023	0.003	2.25E-11	0.002	0.019	9.16E-01
rs10182181	2	25150296	A	G	0.500	-0.031	0.003	8.78E-24	0.001	0.023	9.61E-01
rs10733682	9	129460914	A	G	0.425	0.017	0.003	1.83E-08	0.020	0.017	2.42E-01
rs10938397	4	45182527	A	G	0.567	-0.040	0.003	3.21E-38	-0.027	0.017	1.15E-01
rs10968576	9	28414339	G	A	0.292	0.025	0.003	6.61E-14	0.010	0.018	5.89E-01
rs11030104	11	27684517	A	G	0.800	0.041	0.004	5.56E-28	0.005	0.021	8.15E-01
rs11057405	12	122781897	A	G	0.092	-0.031	0.006	2.02E-08	0.006	0.039	8.82E-01
rs11165643	1	96924097	C	T	0.425	-0.022	0.003	2.07E-12	0.026	0.023	2.58E-01
rs1167827	7	75163169	A	G	0.458	-0.020	0.003	6.33E-10	0.014	0.017	4.17E-01
rs11727676	4	145659064	C	T	0.075	-0.036	0.006	2.55E-08	-0.060	0.039	1.26E-01
rs12286929	11	115022404	G	A	0.433	0.022	0.003	1.31E-12	0.012	0.018	4.84E-01
rs12429545	13	54102206	G	A	0.900	-0.033	0.005	1.09E-12	-0.036	0.033	2.88E-01
rs12940622	17	78615571	A	G	0.458	-0.018	0.003	2.49E-09	0.016	0.023	4.96E-01
rs12986742	2	58975143	C	T	0.500	0.021	0.004	1.01E-08	-0.009	0.017	6.12E-01
rs13078960	3	85807590	T	G	0.817	-0.030	0.004	1.74E-14	0.014	0.021	5.11E-01
rs13107325	4	103188709	C	T	0.883	-0.048	0.007	1.83E-12	0.047	0.036	1.85E-01
rs13191362	6	163033350	A	G	0.800	0.028	0.005	7.34E-09	0.050	0.036	1.67E-01
rs1516725	3	185824004	T	C	0.092	-0.045	0.005	1.89E-22	-0.004	0.026	8.73E-01
rs1528435	2	181550962	T	C	0.583	0.018	0.003	1.20E-08	-0.013	0.018	4.67E-01
rs1558902	16	53803574	A	T	0.450	0.082	0.003	7.51E-153	-0.013	0.017	4.42E-01
rs16851483	3	141275436	G	T	0.908	-0.048	0.008	3.55E-10	0.020	0.035	5.64E-01
rs16951275	15	68077168	C	T	0.225	-0.031	0.004	1.91E-17	0.037	0.020	6.54E-02
rs17001654	4	77129568	C	G	0.842	-0.031	0.005	7.76E-09	-0.048	0.024	4.65E-02
rs17066856	18	58049656	C	T	0.133	-0.040	0.006	6.23E-13	-0.019	0.029	5.22E-01
rs17094222	10	102395440	C	T	0.208	0.025	0.004	5.94E-11	-0.006	0.021	7.68E-01
rs17405819	8	76806584	C	T	0.367	-0.022	0.003	2.07E-11	-0.034	0.019	6.30E-02
rs17724992	19	18454825	A	G	0.692	0.019	0.004	3.42E-08	0.004	0.019	8.45E-01
rs1808579	18	21104888	T	C	0.475	-0.017	0.003	4.17E-08	-0.008	0.017	6.51E-01
rs1928295	9	120378483	C	T	0.425	-0.019	0.003	7.91E-10	0.007	0.017	6.87E-01
rs2033732	8	85079709	C	T	0.758	0.019	0.004	4.89E-08	0.002	0.020	9.12E-01
rs205262	6	34563164	A	G	0.733	-0.022	0.004	1.75E-10	-0.023	0.019	2.16E-01
rs2121279	2	143043285	T	C	0.117	0.025	0.004	2.31E-08	-0.013	0.026	6.06E-01
rs2176598	11	43864278	T	C	0.200	0.020	0.004	2.97E-08	0.001	0.019	9.76E-01
rs2207139	6	50845490	G	A	0.100	0.045	0.004	4.13E-29	0.010	0.023	6.67E-01
rs2245368	7	76608143	T	C	0.758	-0.032	0.006	3.19E-08	0.016	0.029	5.90E-01
rs2287019	19	46202172	C	T	0.850	0.036	0.004	4.59E-18	-0.004	0.022	8.63E-01
rs2303223	16	31075175	A	G	0.375	-0.018	0.003	3.67E-09	-0.017	0.017	3.28E-01
rs2365389	3	61236462	C	T	0.658	0.020	0.003	1.63E-10	-0.021	0.017	2.21E-01
rs2820292	1	201784287	A	C	0.492	-0.020	0.003	1.83E-10	-0.029	0.017	8.87E-02
rs29941	19	34309532	A	G	0.333	-0.018	0.003	2.41E-08	0.013	0.018	4.60E-01
rs3736485	15	51748610	A	G	0.425	0.018	0.003	7.41E-09	-0.003	0.017	8.46E-01
rs3817334	11	47650993	C	T	0.550	-0.026	0.003	5.15E-17	-0.035	0.023	1.35E-01
rs3849570	3	81792112	A	C	0.367	0.019	0.003	2.60E-08	0.020	0.018	2.64E-01
rs3888190	16	28889486	A	C	0.358	0.031	0.003	3.14E-23	0.028	0.017	1.12E-01
rs4740619	9	15634326	T	C	0.533	0.018	0.003	4.56E-09	0.020	0.017	2.54E-01
rs543874	1	177889480	G	A	0.267	0.048	0.004	2.62E-35	0.031	0.021	1.52E-01
rs6477694	9	111932342	C	T	0.358	0.017	0.003	2.67E-08	0.012	0.018	4.95E-01
rs6567160	18	57829135	C	T	0.283	0.056	0.004	3.93E-53	0.048	0.020	1.53E-02
rs657452	1	49589847	A	G	0.417	0.023	0.003	5.48E-13	-0.015	0.018	4.05E-01
rs6656785	1	75005776	G	A	0.383	0.022	0.003	3.83E-12	0.016	0.018	3.59E-01
rs6804842	3	25106437	A	G	0.425	-0.019	0.003	2.48E-09	-0.063	0.023	6.12E-03
rs7138803	12	50247468	G	A	0.558	-0.032	0.003	8.15E-24	0.015	0.018	3.99E-01
rs7141420	14	79899454	T	C	0.617	0.024	0.003	1.23E-14	0.000	0.023	9.90E-01
rs758747	16	3627358	C	T	0.733	-0.023	0.004	7.47E-10	0.015	0.020	4.61E-01
rs7599312	2	213413231	G	A	0.708	0.022	0.003	1.17E-10	0.022	0.019	2.63E-01
rs7899106	10	87410904	A	G	0.950	-0.040	0.007	2.96E-08	0.025	0.040	5.36E-01
rs879620	16	4015729	C	T	0.408	-0.024	0.004	1.06E-09	0.031	0.018	8.25E-02
rs9400239	6	108977663	C	T	0.700	0.019	0.003	1.61E-08	-0.010	0.019	5.95E-01
rs9579083	13	28017270	G	C	0.767	-0.030	0.005	3.46E-10	0.054	0.022	1.29E-02
rs9926784	16	19941968	T	C	0.792	0.027	0.004	1.85E-10	0.000	0.022	9.89E-01

Data source: https://portals.broadinstitute.org/collaboration/giant/index.php/GIANT_consortium_data_files#GWAS_Anthropometric_2015_BMI_Summary_Statistics

SNP: Single Nucleotide Polymorphism
Chr: Chromosome
Pos: Position
EAF: Effect Allele Frequency
SE: Standard Error

Table S11. Type 2 diabetes mellitus replicate

						T2DM GWAS			Breast cancer-specific: All		
**SNP**	**Chr**	**Pos**	**Effect allele**	**Other allele**	**EAF**	**Beta**	**SE**	**P-value**	**Beta**	**SE**	**P-value**
rs1005752	15	77818128	A	C	0.715	0.033	0.008	2.50E-29	-0.007	0.019	7.12E-01
rs10096633	8	19830921	C	T	0.877	0.029	0.010	1.10E-12	-0.024	0.025	8.74E-01
rs10097617	8	95961626	T	C	0.485	0.017	0.008	3.30E-11	0.014	0.017	4.90E-01
rs10193538	2	58981064	T	G	0.610	0.017	0.008	8.90E-09	-0.007	0.018	6.03E-01
rs10195252	2	165513091	T	C	0.586	0.029	0.005	6.00E-25	0.004	0.017	5.79E-01
rs10228066	7	15063569	T	C	0.537	0.029	0.008	1.10E-28	0.001	0.017	5.48E-01
rs10406327	19	33890838	C	G	0.523	0.017	0.008	3.80E-08	-0.006	0.017	5.26E-01
rs10406431	19	46157019	A	G	0.563	0.021	0.005	9.60E-14	-0.013	0.017	5.58E-01
rs1061810	11	43877934	A	C	0.288	0.021	0.008	6.00E-13	-0.003	0.019	2.84E-01
rs10750397	11	128234144	A	G	0.282	0.021	0.008	8.30E-13	-0.032	0.025	2.80E-01
rs10811660	9	22134068	G	A	0.828	0.104	0.013	1.40E-115	-0.021	0.022	8.25E-01
rs10830963	11	92708710	G	C	0.277	0.041	0.008	4.80E-43	0.003	0.020	2.79E-01
rs10842994	12	27965150	C	T	0.805	0.033	0.008	4.10E-20	0.036	0.022	7.94E-01
rs10882101	10	94462427	T	C	0.587	0.025	0.010	1.40E-08	0.003	0.017	5.92E-01
rs10908278	17	36099952	T	A	0.481	0.033	0.008	6.40E-36	0.018	0.017	4.84E-01
rs10938398	4	45186139	A	G	0.429	0.021	0.008	3.60E-12	0.029	0.017	4.30E-01
rs10954772	8	30863938	T	C	0.314	0.017	0.008	1.80E-09	0.000	0.019	3.13E-01
rs10974438	9	4291928	C	A	0.357	0.021	0.008	1.50E-14	0.040	0.019	3.64E-01
rs1127215	1	117532790	C	T	0.584	0.021	0.005	1.60E-13	-0.010	0.018	5.85E-01
rs11496066	7	102486254	T	C	0.818	0.033	0.015	1.10E-08	0.031	0.022	8.12E-01
rs115505614	5	102422968	T	C	0.050	0.076	0.018	1.30E-30	0.022	0.045	4.85E-02
rs11642430	16	30045789	G	C	0.399	0.017	0.005	2.20E-09	0.022	0.017	3.89E-01
rs11680058	2	16574669	A	G	0.863	0.025	0.010	1.40E-08	-0.059	0.043	8.74E-01
rs11688682	2	121347612	G	C	0.728	0.021	0.008	4.20E-09	-0.020	0.025	7.23E-01
rs11699802	20	48832135	C	T	0.536	0.017	0.008	1.80E-11	-0.020	0.017	5.22E-01
rs117001013	22	32348841	C	T	0.912	0.029	0.013	1.70E-08	0.061	0.033	9.26E-01
rs11708067	3	123065778	A	G	0.772	0.037	0.008	5.20E-32	0.031	0.021	7.71E-01
rs11759026	6	126792095	G	A	0.232	0.029	0.008	2.40E-18	0.024	0.021	2.29E-01
rs11842871	13	31042452	G	T	0.735	0.017	0.008	1.20E-08	-0.008	0.027	7.49E-01
rs12001437	9	34074476	C	T	0.372	0.017	0.008	2.80E-10	-0.005	0.018	3.66E-01
rs1260326	2	27730940	C	T	0.607	0.029	0.005	6.50E-25	-0.002	0.017	6.16E-01
rs12640250	4	17792869	C	A	0.715	0.017	0.005	3.70E-08	0.024	0.019	7.20E-01
rs12719778	8	145879883	T	C	0.538	0.017	0.005	5.00E-09	-0.005	0.023	5.25E-01
rs12811407	12	133069698	A	G	0.331	0.021	0.008	1.70E-12	-0.017	0.018	3.28E-01
rs12920022	16	89564055	A	T	0.158	0.021	0.008	3.40E-09	-0.002	0.025	1.49E-01
rs1296328	4	137083193	A	C	0.446	0.017	0.008	3.50E-08	0.010	0.018	4.49E-01
rs13041756	20	21466795	C	T	0.107	0.025	0.010	1.40E-08	0.005	0.029	9.95E-02
rs13262861	8	41508577	C	A	0.829	0.029	0.010	4.00E-12	0.006	0.023	8.15E-01
rs13426680	2	158339550	A	G	0.937	0.037	0.013	6.70E-10	0.058	0.036	9.34E-01
rs1359790	13	80717156	G	A	0.720	0.037	0.008	2.40E-31	0.032	0.019	7.22E-01
rs1377807	17	4045440	C	G	0.312	0.021	0.008	4.20E-13	-0.002	0.019	3.13E-01
rs1412234	9	28410683	C	T	0.323	0.017	0.008	1.90E-10	0.008	0.018	3.17E-01
rs141521721	11	14763828	A	C	0.024	0.053	0.023	2.70E-08	-0.059	0.062	2.15E-02
rs1421085	16	53800954	C	T	0.415	0.053	0.008	3.10E-84	-0.012	0.017	4.07E-01
rs1426371	12	108629780	G	A	0.739	0.021	0.008	8.20E-12	-0.021	0.029	7.37E-01
rs145678014	11	32927778	G	T	0.957	0.045	0.018	2.00E-10	-0.005	0.044	9.58E-01
rs145904381	1	151017991	T	C	0.987	0.076	0.036	2.60E-08	-0.044	0.108	9.87E-01
rs1493694	1	120526982	T	C	0.109	0.037	0.010	2.70E-16	0.021	0.027	1.08E-01
rs1561927	8	129568078	C	T	0.269	0.017	0.008	1.50E-09	-0.023	0.019	2.67E-01
rs1562396	7	130457914	G	A	0.319	0.025	0.008	9.90E-18	0.030	0.024	3.37E-01
rs1580278	4	104140848	C	A	0.473	0.017	0.005	2.20E-10	0.000	0.018	4.69E-01
rs1708302	7	28198677	C	T	0.512	0.041	0.008	1.10E-48	0.024	0.017	5.11E-01
rs17122772	14	23288935	G	C	0.228	0.017	0.008	1.60E-08	-0.003	0.021	2.22E-01
rs17168486	7	14898282	T	C	0.181	0.029	0.008	2.30E-17	0.017	0.023	1.73E-01
rs17250977	5	14753745	G	A	0.038	0.049	0.018	2.00E-11	-0.045	0.071	3.55E-02
rs17522122	14	33302882	T	G	0.474	0.017	0.005	3.20E-09	0.016	0.018	4.67E-01
rs17684074	18	54675384	G	C	0.740	0.017	0.008	2.90E-08	0.004	0.020	7.35E-01
rs17772814	8	128711742	G	A	0.915	0.033	0.015	5.40E-10	0.004	0.031	9.16E-01
rs17791513	9	81905590	A	G	0.932	0.041	0.013	3.10E-14	-0.032	0.034	9.35E-01
rs1783541	11	65294799	T	C	0.204	0.025	0.008	2.00E-14	-0.027	0.022	2.07E-01
rs17836088	14	79932041	C	G	0.217	0.025	0.010	6.70E-14	0.004	0.021	2.18E-01
rs1796330	12	71522953	G	C	0.571	0.021	0.005	2.20E-14	0.003	0.018	5.63E-01
rs1800961	20	43042364	T	C	0.035	0.072	0.020	2.30E-22	-0.038	0.048	3.60E-02
rs184509201	10	114740337	C	G	0.982	0.083	0.031	1.20E-13	0.084	0.064	9.81E-01
rs1903002	4	89740894	G	C	0.501	0.017	0.008	2.70E-08	0.006	0.017	5.17E-01
rs2102278	4	52818664	G	A	0.319	0.017	0.008	3.70E-08	0.009	0.019	3.12E-01
rs2197973	12	95928560	T	C	0.538	0.017	0.008	3.60E-08	0.002	0.018	5.33E-01
rs2258238	12	66221060	T	A	0.104	0.041	0.013	4.50E-21	0.041	0.027	1.06E-01
rs2268078	20	32596704	A	G	0.657	0.017	0.008	2.30E-10	-0.011	0.018	6.82E-01
rs2272163	3	77671721	C	A	0.618	0.017	0.008	9.60E-09	0.020	0.023	6.06E-01
rs2283220	11	2755548	A	G	0.690	0.021	0.008	1.40E-09	0.006	0.019	6.97E-01
rs243024	2	60583665	A	G	0.460	0.025	0.005	2.50E-20	-0.013	0.017	4.60E-01
rs2796441	9	84308948	G	A	0.592	0.029	0.008	4.40E-24	-0.014	0.023	5.93E-01
rs28505901	9	139241030	G	A	0.752	0.037	0.010	6.70E-26	0.035	0.027	7.43E-01
rs2925979	16	81534790	T	C	0.300	0.021	0.008	1.40E-14	0.011	0.019	2.98E-01
rs3111316	19	13038415	A	G	0.589	0.021	0.008	6.30E-13	0.012	0.023	5.94E-01
rs329122	5	133864599	A	G	0.429	0.017	0.005	3.60E-09	0.015	0.017	4.13E-01
rs340874	1	214159256	C	T	0.556	0.029	0.008	1.60E-22	0.027	0.018	5.45E-01
rs34584161	13	26776999	A	G	0.760	0.021	0.008	2.20E-10	0.027	0.020	7.61E-01
rs34715063	15	38873115	C	T	0.124	0.041	0.013	2.30E-19	0.045	0.040	1.18E-01
rs348330	1	229672955	G	A	0.361	0.021	0.008	2.70E-14	0.011	0.019	3.55E-01
rs34965774	12	118412373	A	G	0.144	0.025	0.010	2.00E-09	-0.019	0.033	1.33E-01
rs35352848	3	23455582	T	C	0.788	0.029	0.010	1.30E-17	-0.014	0.028	8.01E-01
rs35895680	17	47060322	C	A	0.678	0.025	0.008	2.50E-15	-0.012	0.024	6.74E-01
rs35999103	2	147861633	T	C	0.155	0.021	0.010	9.70E-09	-0.056	0.032	1.53E-01
rs3751837	16	3583173	T	C	0.220	0.017	0.008	1.40E-08	-0.011	0.022	2.26E-01
rs3768321	1	40035928	T	G	0.200	0.037	0.008	2.60E-26	0.000	0.029	2.03E-01
rs3798519	6	50788778	C	A	0.184	0.025	0.010	2.60E-12	0.002	0.022	1.89E-01
rs3802177	8	118185025	G	A	0.685	0.045	0.008	1.10E-55	0.008	0.018	6.97E-01
rs3845281	5	14610134	G	A	0.904	0.033	0.010	2.30E-11	-0.043	0.030	9.08E-01
rs3887925	3	186665645	T	C	0.547	0.029	0.008	3.10E-22	0.014	0.017	5.57E-01
rs4238013	12	4376089	C	T	0.209	0.025	0.008	3.20E-11	0.046	0.028	2.09E-01
rs429358	19	45411941	T	C	0.846	0.033	0.010	2.60E-18	0.012	0.025	8.61E-01
rs4457053	5	76424949	G	A	0.304	0.025	0.008	8.40E-18	-0.019	0.019	3.02E-01
rs4688760	3	49980596	T	C	0.684	0.017	0.008	3.50E-10	-0.005	0.019	6.77E-01
rs4709746	6	164133001	C	T	0.868	0.025	0.010	5.80E-09	0.009	0.034	8.65E-01
rs474513	6	160770312	A	G	0.517	0.017	0.005	8.10E-10	-0.002	0.017	5.08E-01

rs4804833	19	7970635	A	G	0.390	0.021	0.008	7.70E-13	0.019	0.017	3.96E-01
rs4925109	17	17661802	A	G	0.316	0.021	0.008	2.80E-12	-0.002	0.018	3.27E-01
rs4929965	11	2197286	A	G	0.383	0.029	0.008	4.00E-26	-0.017	0.017	3.86E-01
rs4932265	15	90423293	T	C	0.267	0.029	0.008	4.20E-20	0.021	0.026	2.57E-01
rs4946812	6	107431688	G	A	0.674	0.017	0.005	8.20E-09	-0.023	0.024	6.75E-01
rs4977213	8	145507304	C	T	0.375	0.021	0.008	9.10E-14	0.010	0.024	3.75E-01
rs505922	9	136149229	C	T	0.332	0.021	0.008	3.90E-12	-0.016	0.018	3.57E-01
rs5213	11	17408404	C	T	0.362	0.029	0.008	3.50E-27	0.028	0.018	3.53E-01
rs539515	1	177889025	C	A	0.198	0.021	0.008	1.60E-10	0.032	0.021	1.87E-01
rs55653563	9	97001682	A	C	0.732	0.017	0.008	2.20E-09	0.027	0.019	7.33E-01
rs56348580	12	121432117	G	C	0.689	0.021	0.008	2.30E-13	0.026	0.018	6.99E-01
rs58432198	1	51256091	C	T	0.881	0.029	0.010	2.10E-10	-0.091	0.027	8.85E-01
rs58730668	4	185717759	T	C	0.858	0.029	0.010	1.30E-13	0.029	0.026	8.56E-01
rs601945	6	32573415	G	A	0.178	0.025	0.010	4.70E-08	-0.004	0.031	1.29E-01
rs60276348	17	62203304	T	C	0.140	0.021	0.010	2.60E-08	-0.074	0.036	1.40E-01
rs6070625	20	57394628	G	C	0.517	0.021	0.005	5.30E-14	0.000	0.017	5.33E-01
rs61676547	17	65892507	C	G	0.192	0.025	0.008	2.90E-11	0.022	0.021	1.95E-01
rs62007683	14	103894071	G	T	0.653	0.017	0.008	3.10E-08	-0.022	0.023	6.33E-01
rs62080313	18	36278709	C	T	0.123	0.025	0.010	1.00E-08	-0.016	0.025	1.29E-01
rs62271373	3	150066540	A	T	0.055	0.037	0.015	1.00E-09	-0.021	0.052	5.20E-02
rs6458354	6	43814190	C	T	0.289	0.021	0.008	2.10E-12	-0.004	0.019	2.90E-01
rs6459733	7	156930550	G	C	0.673	0.025	0.005	2.40E-17	-0.011	0.019	6.67E-01
rs6518681	22	30609554	G	A	0.914	0.037	0.013	1.10E-12	0.016	0.031	9.22E-01
rs67232546	11	128398938	T	C	0.207	0.025	0.008	1.30E-11	0.015	0.022	2.01E-01
rs6780171	3	185503456	A	T	0.314	0.057	0.010	9.00E-56	0.005	0.018	3.08E-01
rs6821438	4	95091911	A	G	0.534	0.017	0.008	4.00E-11	0.020	0.017	5.32E-01
rs6976111	7	117495667	A	C	0.313	0.017	0.008	1.20E-08	0.022	0.018	3.21E-01
rs7022807	9	19067833	G	A	0.401	0.017	0.005	2.70E-10	-0.018	0.017	3.88E-01
rs702634	5	53271420	A	G	0.690	0.021	0.008	7.70E-14	-0.005	0.018	6.96E-01
rs703972	10	80952826	G	C	0.533	0.029	0.008	1.70E-29	0.019	0.017	5.38E-01
rs71372253	17	29413019	C	T	0.064	0.033	0.013	4.40E-08	-0.091	0.047	6.20E-02
rs7178762	15	63871292	C	T	0.460	0.017	0.005	5.40E-10	-0.013	0.018	4.64E-01
rs7222481	17	9785187	C	G	0.324	0.017	0.005	1.40E-08	0.002	0.025	2.97E-01
rs72802342	16	75234872	C	A	0.923	0.068	0.015	4.00E-32	0.051	0.044	9.25E-01
rs72926932	18	53050646	C	A	0.084	0.037	0.013	1.00E-14	0.000	0.031	8.20E-02
rs738408	22	44324730	T	C	0.226	0.021	0.010	1.40E-10	0.048	0.021	2.23E-01
rs7629630	3	168218841	A	T	0.857	0.021	0.010	2.50E-08	0.015	0.033	8.59E-01
rs7669833	4	153513369	T	A	0.705	0.025	0.008	1.20E-14	-0.013	0.025	7.13E-01
rs7719891	5	86577352	G	A	0.259	0.017	0.008	2.40E-08	0.024	0.020	2.66E-01
rs77464186	11	72460398	A	C	0.836	0.045	0.010	4.70E-33	0.003	0.024	8.40E-01
rs7756992	6	20679709	G	A	0.274	0.061	0.010	2.40E-88	0.021	0.019	2.80E-01
rs77864822	12	97848775	A	G	0.932	0.033	0.015	1.10E-08	-0.029	0.035	9.31E-01
rs7987740	13	109947213	T	C	0.609	0.017	0.008	4.00E-08	0.011	0.018	6.06E-01
rs8010382	14	91963722	G	A	0.421	0.017	0.005	6.50E-09	0.032	0.018	4.20E-01
rs80147536	2	43698028	A	T	0.904	0.053	0.013	2.70E-29	0.021	0.029	9.08E-01
rs8037894	15	62394264	G	C	0.566	0.021	0.008	2.60E-13	0.020	0.018	5.62E-01
rs8107974	19	19388500	T	A	0.077	0.041	0.013	3.30E-15	0.007	0.031	7.80E-02
rs878521	7	44255643	A	G	0.245	0.025	0.008	1.90E-13	-0.027	0.027	2.49E-01
rs9430095	1	206593900	C	G	0.494	0.017	0.008	1.90E-08	-0.019	0.023	4.95E-01
rs9494624	6	137300960	A	G	0.290	0.017	0.008	6.10E-09	0.010	0.026	2.90E-01
rs9563615	13	59077406	A	T	0.710	0.021	0.008	6.40E-11	0.011	0.019	7.13E-01
rs9687832	5	55861595	A	G	0.198	0.033	0.010	1.70E-20	-0.023	0.022	1.98E-01
rs9860730	3	64701146	A	G	0.704	0.025	0.008	4.90E-15	0.029	0.019	6.99E-01
rs9873618	3	170733076	G	A	0.710	0.029	0.008	4.80E-21	0.000	0.025	7.07E-01
rs9957145	18	56876228	G	A	0.829	0.021	0.010	8.10E-09	0.015	0.023	8.40E-01

Data source: http://diagram-consortium.org/

SNP: Single Nucleotide Polymorphism
Chr: Chromosome
Pos: Position
EAF: Effect Allele Frequency
SE: Standard Error
